# MaskGraphene: an advanced framework for interpretable joint representation for multi-slice, multi-condition spatial transcriptomics

**DOI:** 10.1186/s13059-025-03850-w

**Published:** 2025-11-05

**Authors:** Yunfei Hu, Zhenhan Lin, Manfei Xie, Weiman Yuan, Yikang Li, Mingxing Rao, Yichen Henry Liu, Wenjun Shen, Lu Zhang, Xin Maizie Zhou

**Affiliations:** 1 Department of Computer Science, Vanderbilt University, Nashville, USA; 2 Department of Biomedical Engineering, Vanderbilt University, Nashville, USA; 3 Department of Bioinformatics, Shantou University Medical College, Shantou, China; 4 Department of Computer Science, Hong Kong Baptist University, Kowloon Tong, Hong Kong

**Keywords:** Spatial Transcriptomics, Batch correction, Integration, Interpretable embeddings, Self-supervised learning, Contrastive learning

## Abstract

Recent advances in spatial transcriptomics (ST) highlight the need to integrate multiple slices for joint analysis. A key challenge is generating interpretable embeddings that preserve spatial geometry while correcting batch effects. We present MaskGraphene, a graph neural network that integrates ST data via masked self-supervised learning, triplet loss, and cluster-wise local alignment. By establishing indirect “soft-links” and direct “hard-links” across slices, MaskGraphene yields joint embeddings with high geometric fidelity. Benchmarks against eight methods demonstrate superior alignment and interpretability. MaskGraphene enhances downstream applications, including domain identification, trajectory reconstruction, biomarker discovery, and brain-layer mapping, enabling robust ST integration and biological insight.

**Supplementary Information:**

The online version contains supplementary material available at 10.1186/s13059-025-03850-w.

## Background

The intricate orchestration of biological processes within organisms hinges upon the diversity and specialization of individual cell types, each tailored to fulfill specific functions. To unravel the complexities of disease pathology and tissue functioning, understanding the connections and interactions of neighboring and distant cells is crucial, since the behavior of cells is profoundly influenced by their microenvironment [1]. Although single-cell RNA sequencing (scRNA-seq) has revolutionized our ability to profile gene expression at single-cell resolution, its lack of spatial context limits our understanding of cellular niches within their native microenvironments. This limitation impedes our understanding of key processes such as cell-cell interactions, tissue organization, and spatially regulated functional dynamics, all of which are vital for deciphering biological systems [2, 3].

Advancements in Spatial Transcriptomics (ST) have bridged the gap between transcriptomic profiling and spatial context by enabling simultaneous measurement of mRNA expression and spatial coordinates within tissue sections [4]. These techniques have significantly advanced our ability to explore the complex transcriptional landscapes of heterogeneous tissues [5]. Existing methods can be broadly categorized into imaging-based techniques, such as smFISH, STARmap, MERFISH, seqFISH, and seqFISH+ [6–10], which offer high spatial resolution, and sequencing-based techniques, including Slide-seq, Slide-seqV2, 10x Visium, HDST, and Stereo-seq, which provide scalable data with varying resolution [11–15]. Despite these advancements, the integrated analysis of ST datasets generated under different experimental conditions or technologies remains a significant challenge [16].

Conventional single-slice ST data analyses primarily focus on uncovering spatial domain distributions within individual tissue sections [17–19]. Recently, there has been growing recognition of the value of integrative and comparative analysis of ST datasets from diverse sources, including different samples, biological conditions, technological platforms, and developmental stages [20]. Integrative analysis provides a more comprehensive understanding of spatial tissue structures, enabling deeper insights into complex spatial organization and leveraging additional information for more robust analyses. Nevertheless, ST datasets are susceptible to batch effects, which can obscure true biological signals and complicate data interpretation. Therefore, developing a well-designed multi-slice ST data integration framework that jointly models and harmonizes signals across slices while mitigating batch effects is an urgent and critical need.

Existing integration strategies often focus on aggregating gene expression data across slices or applying batch correction techniques originally developed for scRNA-seq, such as Harmony [21] and Seurat [22]. These approaches often neglect the vital spatial coordinates of ST data. To address these limitations, advanced tools such as BASS [23], DeepST [20], PRECAST [24], GraphST [25], SPIRAL [26], STAligner [27], SpaDo [28], and SpaMask [29] have been developed. These methods employ a wide variety of strategies. BASS applies a hierarchical Bayesian model framework for multi-slice clustering, producing clustering labels as outputs. DeepST utilizes a variational graph autoencoder combined with data augmentation techniques to enable joint analysis of ST slices. PRECAST leverages a Gaussian mixture model and hidden Markov random fields to derive latent joint embeddings. GraphST, recognized for its strong performance on single-slice clustering tasks, extends its capabilities to multi-slice analysis by constructing positive and negative spot pairs for contrastive training using representations of both normal and corrupted graphs. SPIRAL employs a graph autoencoder with an optimal transport-based discriminator and a classifier to mitigate batch effects, align spatial coordinates, and refine gene expression. STAligner integrates a graph attention autoencoder with a triplet loss strategy to enhance spatial alignment and embedding quality. SpaDo calculates a unified Spatially Adjacent Cell Type Embedding (SPACE) for multiple slices. This is achieved by concatenating individual slice embeddings and employing Jensen-Shannon divergence and Manhattan distance for hierarchical clustering, ensuring cross-slice comparability through consistent cell type annotations. SpaMask leverages a masked self-supervised and contrastive learning framework by simultaneously masking edge structures and gene expression features, while relying on external alignment methods (e.g., ICP [30] or PASTE [31]) to pre-align tissue slices prior to embedding optimization.

With the exception of BASS, all of these methods focus on learning joint embeddings for integration analysis. While they have shown varying degrees of effectiveness in multi-slice analyses [16], they share a common limitation inherent to many graph-based deep learning models: the inability to generate interpretable joint embeddings that accurately capture geometric information. This shortfall hinders precise node-wise (or spot-wise) alignment across slices, ultimately limiting their effectiveness in ST data integration and other downstream analyses.

Besides integration methods, several state-of-the-art alignment approaches - PASTE [31], PASTE2 [32], SPACEL [33], STalign [34], and GPSA [35] - have been proposed to align adjacent or partially overlapping slices and reconstruct consistent 2D/3D tissue coordinates. PASTE frames alignment as fused Gromov-Wasserstein optimal transport over expression and spatial distances to preserve local spatial proximity across adjacent slices, while PASTE2 generalizes this approach to partial overlap for multi-slice stacks. STalign performs diffeomorphic metric mapping to handle nonlinear deformations and partial tissue matches. SPACEL uses a deep learning framework to infer cross-slice correspondences and recover 3D spatial architectures. GPSA models alignment with a two-layer deep Gaussian process that warps slices into a common coordinate system conditioned on phenotypic readouts. In contrast to integration methods, transformed coordinates, rather than joint embeddings are the main outputs.

To address the challenges of integrating ST data while producing interpretable, batch-corrected joint embeddings, we introduce MaskGraphene, a graph neural network that combines self-supervised and self-contrastive training strategies with a local alignment module. MaskGraphene integrates gene expression and spatial context data to generate joint embeddings suitable for integration and various downstream analyses. To preserve geometric interpretability, the model introduces two types of inter-slice connections: “hard-links”, which establish direct spot-to-spot mappings across slices through spot-wise local alignment using k-NN graphs, and “soft-links”, which create indirect connections through triplets constructed in the latent embedding space. Furthermore, its masked self-supervised loss enforces reconstruction in the embedding space while regularizing the node feature reconstruction, thereby enhancing both interpretability and robustness.

We benchmarked MaskGraphene against eight integration methods across a diverse range of spatial transcriptomics datasets, evaluating performance for various integration scenarios and quantitative metrics. MaskGraphene consistently achieved the highest alignment accuracy and matching performance among all tools. Notably, its joint embeddings, visualized in two-dimensional UMAP, are the first to effectively capture the overall slice shape, partially overlapping patterns, spatial structures, and geometric relationships. Taking advantage of its interpretable joint embeddings, MaskGraphene excelled in multiple integration scenarios and downstream analyses, including enhanced domain identification, reconstruction of spatial trajectories across slices, improved biomarker identification, generation of topographical maps of brain slices, identification of neuronal differentiation and activity gradients, simultaneous characterization of unique and shared tissue structures across horizontally consecutive slices, and alignment of tissue structures across developmental stages. In ablation studies, we observed that some model variants achieved slightly higher iLISI scores than our proposed model, indicating a greater degree of batch mixing. However, MaskGraphene consistently outperformed all variants in alignment accuracy and geometry preservation metrics. This points out that our model makes a deliberate trade-off - sacrificing a small amount of mixing-in order to better preserve biologically meaningful spatial structures and improve clustering fidelity.

## Results

### Overview of MaskGraphene

**Fig. 1:**
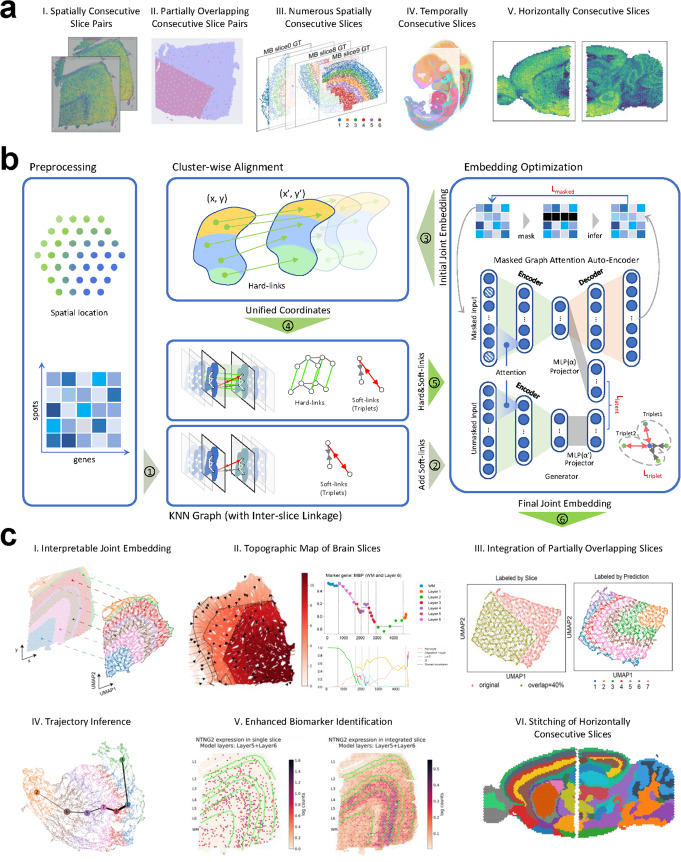
MaskGraphene workflow. **a** Illustration of spatial transcriptomics data integration scenarios addressed by MaskGraphene, including spatially consecutive slice pairs (I), simulated partially overlapping consecutive slice pairs (II), numerous spatially consecutive slices (III), temporally consecutive slices (IV), and horizontally consecutive slices (V). **b** Workflow of MaskGraphene: The preprocessing step organizes spatial coordinates and gene expression data. Inter-slice linkage is established through the construction of “hard-links” via cluster-wise local alignment and “soft-links” using contrastive learning with triplets. Embedding optimization leverages a masked graph autoencoder to generate batch-corrected joint embeddings by optimizing masked self-supervised loss ($L_{masked} + L_{latent}$) and triplet loss. Steps 1-5 of the main method are indicated with numbered circles. **c** Applications and evaluations (Step 6): (I) Interpretable joint embedding captures the original geometric structure. (II) Topographic map of brain slices with isodepth analysis reveals gene expression gradients across cortical layers. (III) Validation with simulated data demonstrates robust integration of partially overlapping slices. (IV) Trajectory inference reveals linearly connected developmental trends. (V) Alignment and integration of embryonic tissue structures enhance biomarker identification. (VI) Stitching of horizontally consecutive slices reconstructs spatially coherent regions

The overall workflow (Fig. 1) of MaskGraphene processes a spot-gene expression matrix and spatial coordinates from two or more tissue slices, tailored to various integration scenarios. In Steps 1-2, MaskGraphene begins with an initial integration step using a masked graph attention autoencoder backbone with an intra-slice k-NN graph, jointly optimized with a masked self-supervised reconstruction loss ($L_{masked} + L_{latent}$ in Fig. 1) [36–38] and a triplet loss to identify shared clusters (domains) across slices. Triplet losses serve as “soft-links” in the latent space to strengthen inter-slice connections. An alternating strategy is used to minimize triplet loss: every 100 training epochs, the triplets are updated using the latest low-dimensional embeddings, progressively refining triplets and enabling triplet loss, and reconstruction loss, to be minimized together. In Step 3, MaskGraphene performs cluster-wise local alignment for all pairs of consecutive slices to align spots across slices, establishing direct mapping (“hard-links”) that further strengthen inter-slice connections. Finally, in Steps 4-5, these hard-links are used to construct an inter-slice k-NN graph, which is input to the same masked graph attention autoencoder to learn final joint embeddings. More details of the algorithm are provided in the Methods section.

We benchmarked MaskGraphene against eight integration methods under various experimental scenarios: BASS, DeepST, PRECAST, GraphST, SPIRAL, STAligner, SpaDo, and SpaMask. The details for each tool are provided in Additional file 1: Table S1.

### Enhanced alignment and mapping accuracy achieved through joint embeddings in MaskGraphene

To assess whether MaskGraphene generates superior joint latent embeddings for integrating consecutive slices compared to existing integration methods such as DeepST, STAligner, GraphST, PRECAST, SPIRAL, SpaDo, and SpaMask, we conducted two evaluation experiments involving nine pairs of human dorsolateral prefrontal cortex (DLPFC) slices and four pairs of mouse hypothalamus (MHypo) slices. The DLPFC dataset, generated using 10x Visium, includes 12 human sections with annotations for cortical layers 1–6 and white matter (WM) from three samples [39]. Each sample has four consecutive slices (A, B, C, D), with AB and CD being 10$\mu$m apart and BC separated by 300$\mu$m. The MHypo dataset by MERFISH consists of five consecutive slices [23], labeled Bregma -0.04mm, -0.09mm, -0.14mm, -0.19mm, and -0.24mm, each with detailed cell annotations. Additional details about these datasets can be found in the Methods section.

**Fig. 2:**
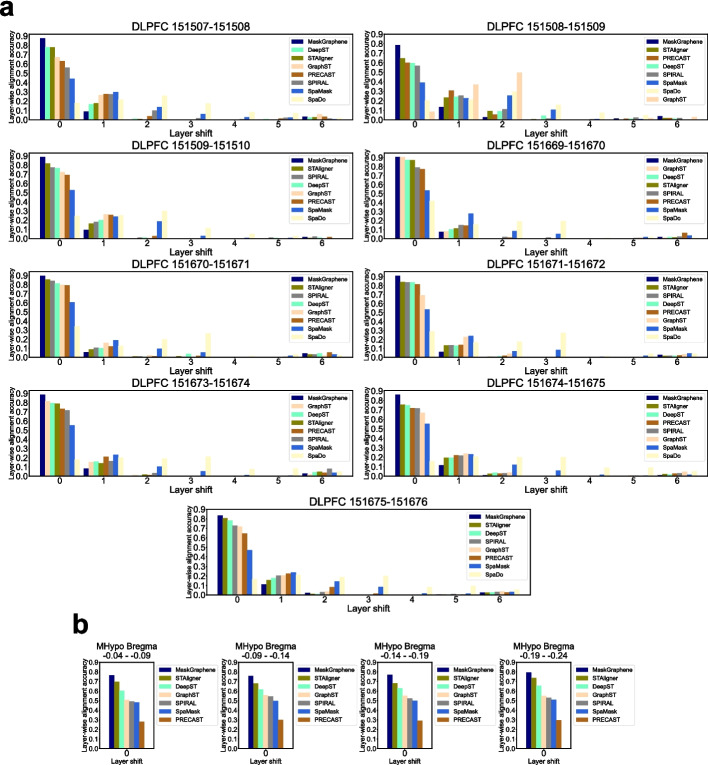
Bar plots for layer-wise alignment accuracy on DLPFC and MHypo datasets. **a** Bar plots illustrating the layer-wise alignment accuracy across layer shifts ranging from 0 to 6, comparing eight various methods on nine DLPFC slice pairs. **b** Bar plots illustrating the layer-wise alignment accuracy for a layer shift of 0, comparing eight various methods on four MHypo slice pairs. Tools are sorted in descending order based on the accuracy for layer shift of 0 in (**a**, **b**)

The first evaluation focused on layer-wise alignment accuracy [16], based on the critical hypothesis that aligned spots across adjacent consecutive slices are more likely to belong to the same spatial domain or cell type. We utilized joint embeddings learned from all tools, based on Euclidean distance, to align (anchor) spots from the first slice to the corresponding (aligned) spots on the second slice for each slice pair. In Fig. 2a, we compared the layer-wise alignment accuracy of eight methods across all nine DLPFC slice pairs. Leveraging the distinctive layered structure of the DLPFC data, this evaluation metric was designed to assess whether anchor and aligned spots belonged to the same layer (layer shift = 0) or to different layers (layer shift = 1 to 6). An effective integration tool would be expected to achieve high accuracy for anchor and aligned spots within the same layer, with accuracy progressively decreasing as the layer shift increased. In Fig. 2a, we plotted the layer-wise alignment accuracy and ranked the tools in descending order. MaskGraphene consistently exhibited the highest accuracy (for a layer shift of 0) across all nine DLPFC slice pairs, outperforming all other tools. For all tools, the majority of anchor and aligned spots across two consecutive slices were mapped to the same layer. However, distant DLPFC slice pairs (300$\mu$m apart), such as DLPFC 151508-151509, presented challenges for all methods except MaskGraphene. In these cases, layer-wise alignment accuracy (for a layer shift of 0) substantially decreased compared to nearby slice pairs (10$\mu$m apart), with a large proportion of anchor spots aligning to adjacent layers (1 or 2 layers away).

A similar experiment was performed on four pairs from the MHypo dataset (Fig. 2b). Here, layer-wise alignment accuracy was only assessed for a layer shift of 0, as the hypothalamus lacks a distinct layered structure. MaskGraphene outperformed all other methods, with STAligner and DeepST ranking second and third, respectively. We attribute MaskGraphene’s high alignment accuracy to its use of inter-slice “hard-links”, which effectively facilitate the connection of similar spots or regions across slices.

**Fig. 3:**
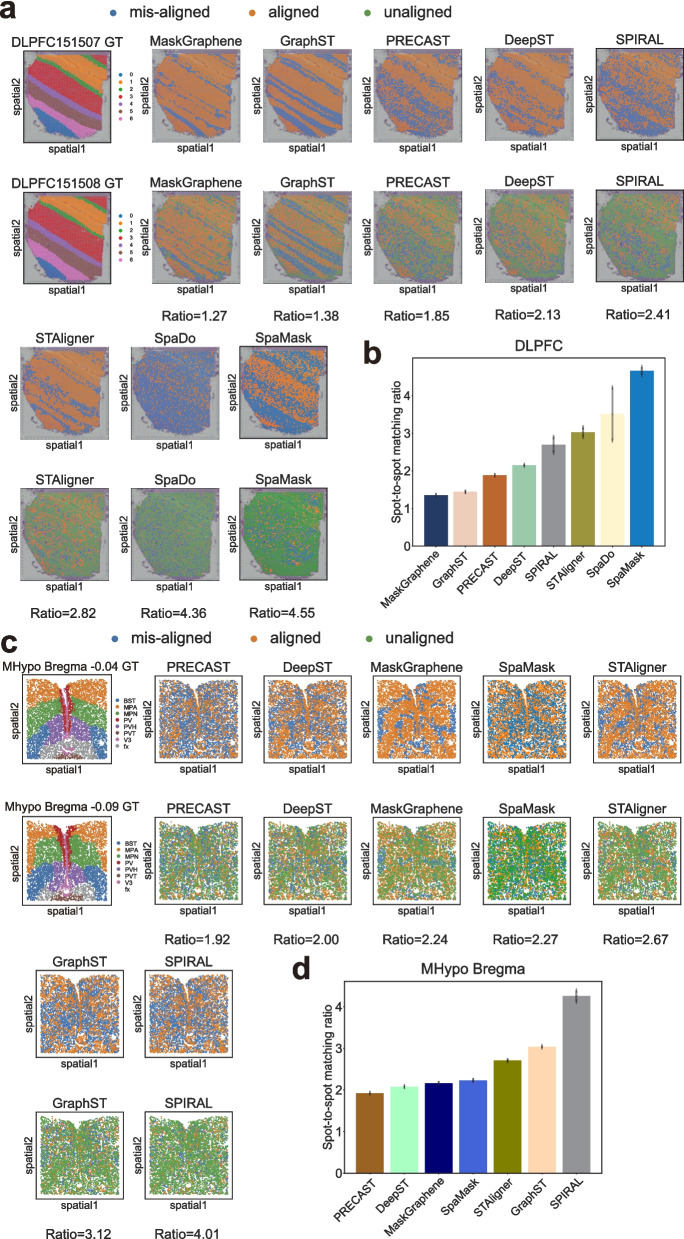
Visualization plots for alignment-misalignment-unalignment and spot-to-spot mapping ratio on DLPFC and MHypo datasets. **a** Visualization plots displaying aligned spots, misaligned spots, and unaligned spots for DLPFC 151507-151508 pair. The anchor spot from the first (top) slice is aligned to the corresponding spots on the second (bottom) slice. The first slice pair illustrating ground truth (GT) annotations. The values below each plot indicate the spot-to-spot matching ratio. **b** Bar plots representing the average spot-to-spot mapping ratio of each tool on the DLPFC dataset. **c** Visualization plots displaying aligned spots, misaligned spots, and unaligned spots for MHypo Bregma -0.04 - -0.09 pair (**d**). Bar plots representing the average spot-to-spot mapping ratio of each tool on the MHypo dataset

To further evaluate the quality of the joint embeddings, we calculate the spot-to-spot matching ratio [16], a metric that quantifies how well the spots in one slice align with their corresponding spots in the adjacent slice. For the DLPFC 151507-151508 pair (Fig. 3a), we designated spots on the first slice as “anchors” and identified their corresponding “aligned” spots on the second slice based on Euclidean distances computed from the joint spot embeddings. Using ground truth layer labels, we further categorized all spots into three groups: aligned (orange, anchor and aligned spots belong to the same layer), misaligned (blue, anchor and aligned spots are from different layers), and unaligned (green, spots on the second slice are not matched to any anchor spots). MaskGraphene (1.27), GraphST (1.38), and PRECAST (1.85) exhibited lower spot-to-spot matching ratios (below 2), indicating superior one-to-one alignment fidelity. In contrast, methods such as SpaMask (4.55), SpaDo (4.36), STAligner (2.82), SPRIAL (2.41), and DeepST (2.13) showed higher matching ratios, reflected in an increased number of unaligned spots on the second slice. This is likely due to a tendency to map multiple anchor spots (2-5) to a single aligned spot, resulting in a many-to-one integration pattern. While this may still achieve good layer-wise alignment, it leads to a higher spot-to-spot matching ratio. These trends were consistently observed on other DLPFC pairs of different samples by all tools, as shown in Additional file 1: Fig. S1. Averaging the spot-to-spot ratio across all nine DLPFC pairs (Fig. 3b) showed that MaskGraphene (1.31) consistently achieved the lowest ratio, indicating the best performance, followed by GraphST (1.38), PRECAST (1.85), and DeepST (2.13). In contrast, tools such as SPIRAL (2.41), STAligner (2.78), SpaDo (3.30), and SpaMask (4.32) exhibited higher ratios. Moreover, across all nine pairs, misaligned spots on both slices were observed to aggregate along layer boundaries as thin, tightly clustered layers in MaskGraphene and GraphST. Conversely, the other tools showed a more dispersed pattern of misaligned spots within the layers. These results indicate that while better performing tools primarily struggle with aligning spots near domain boundaries, worse performing tools misalign spots even within individual domains.

For the MERFISH MHypo dataset, unlike the DLPFC dataset, the transcriptomic spots exhibit an irregular spatial distribution, and the slice pairs are separated by a greater distance (0.05mm apart). Similar to the decreased layer-wise alignment accuracy (Fig. 2b vs. a), the performance of all methods either declined or remained poor in terms of spot-to-spot matching ratios compared to the DLPFC dataset, as shown in Fig. 3c-d and Additional file 1: Fig. S2. Specifically, PRECAST (1.92), DeepST (2.00), MaskGraphene (2.24), SpaMask (2.27) exhibited relatively lower ratios compared to STAligner (2.67), GraphST (3.12), and SPRIAL (4.01). The average ratio across all four pairs in Fig. 3d reflected a similar trend. Although PRECAST and DeepST achieved slightly lower matching ratio than MaskGraphene, their misaligned spots were scattered within the spatial domains (Fig. 3c and Additional file 1: Fig. S2), consistent with their pattern observed in the DLPFC dataset. In contrast, MaskGraphene exhibited thin, tightly aggregated lines of misaligned spots near the domain boundaries. For both the DLPFC and MHypo datasets, we conducted ablation studies evaluating layer-wise alignment accuracy and spot-to-spot matching ratios. Details of the ablation studies are provided in the Methods section. As shown in Additional file 1: Fig. S3, MaskGraphene consistently achieved the highest alignment accuracy across both datasets, demonstrating its strong ability to preserve local correspondences. In contrast, the MaskGraphene-MLP and MaskGraphene-GAE baselines showed substantially lower accuracy, highlighting the importance of incorporating both graph structure and masking strategies. Furthermore, removing either soft-link (MaskGraphene-HardlinkOnly) or hard-link information (MaskGraphene-SoftlinkOnly) led to reduced alignment accuracy relative to the full model, with the largest drops observed in distant pairs such as DLPFC 151508-151509 and 151670-151671. These findings highlight that the removal of either component compromises performance. Consistently, the full model achieved the highest average spot-to-spot matching ratio across both datasets, as shown in Additional file 1: Fig. S4.

Since both MaskGraphene and PASTE use optimal transport (OT) for alignment - MaskGraphene for cluster-wise local alignment and PASTE for global alignment - we sought to assess whether the local alignment strategy in MaskGraphene offers robust support for effective integration. Specifically, we implemented Graphene-PASTE, by replacing MaskGraphene’s local alignment module with PASTE, and computed layer-wise alignment accuracy and spot-to-spot matching ratios. We then visualized aligned, misaligned, and unaligned spots for PASTE and MaskGraphene-PASTE on the DLPFC and MHypo datasets. As shown in Additional file 1: Fig. S5a, both PASTE and MaskGraphene-PASTE exhibited low layer-wise alignment accuracy for distant DLPFC slice pairs (300$\mu$m apart), such as 151508-151509 and 151670-151671. The visualizations in Additional file 1: Fig. S6b further illustrate this layer shift misalignment, with misaligned spots (blue) distributed across multiple layers in DLPFC 151508-151509 for both PASTE and MaskGraphene-PASTE. These results suggest that our local alignment approach is more effective at handling distant, partially overlapping slices. For nearby slice pairs, such as DLPFC 151507-151508, PASTE and MaskGraphene-PASTE showed a misalignment pattern similar to that of MaskGraphene. Consistent with prior observations, misaligned spots on both slices predominantly clustered along cortical layer boundaries, forming thin, tightly packed bands (Additional file 1: Fig. S6a and c). This pattern likely reflects biological ambiguity at domain boundaries, where spots often capture mixed transcriptional signals from adjacent layers. Such transitional zones are known to exhibit gradual molecular gradients rather than abrupt shifts, making them more susceptible to alignment uncertainty. This pattern was also observed in the MHypo dataset (Additional file 1: Fig. S5b and Fig. S7). Notably, PASTE achieved a spot-to-spot matching ratio close to 1 for both datasets, as it directly aligns spots through mapping. Overall, MaskGraphene achieved better alignment and mapping accuracy in the latent space compared to MaskGraphene-PASTE.

In summary, MaskGraphene demonstrates the best alignment and mapping performance, achieved through its joint embeddings. For deep learning-based methods, it is common for spots in low-dimensional space to lose some geometric information from the original gene expression and spatial coordinate profile during optimization. As a result, these tools tend to exhibit poorer spot-to-spot alignment performance, but achieve better layer-wise alignment accuracy. By introducing “hard-links” to strengthen inter-slice connections, MaskGraphene imposes additional constraints on the optimization process, enabling its joint embeddings to better preserve the original geometric structure and improve spot-to-spot alignment.

### MaskGraphene enhances integration with interpretable joint embedding to preserve the original geometric structure and reconstruct spatial trajectory

**Fig. 4:**
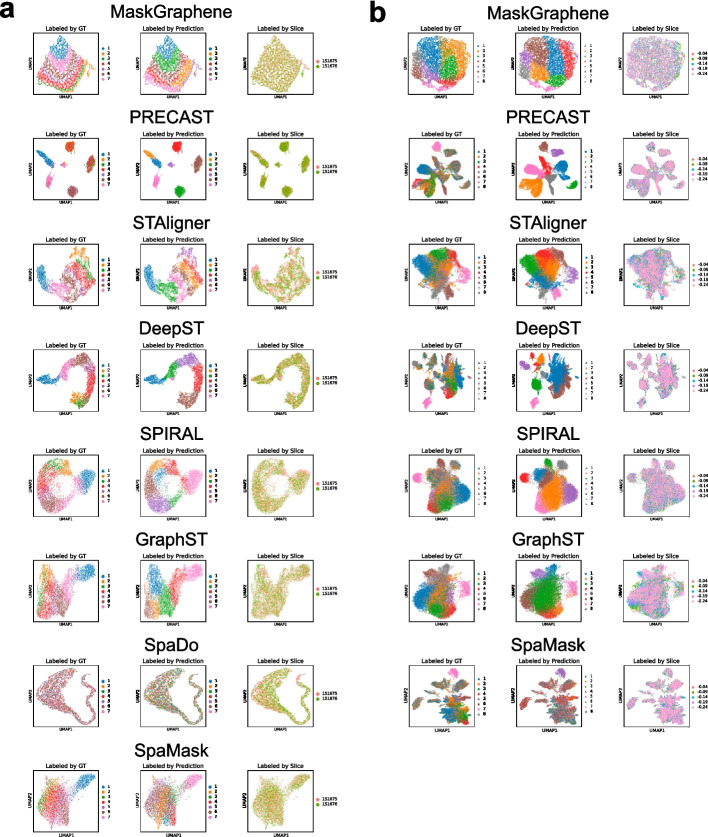
UMAP plots of low dimensional joint embedding on the DLPFC and MHypo datasets. **a** UMAP visualizations of joint embeddings generated by different methods for the DLPFC 151675-151676 pairwise integration. Spots are colored by ground truth (GT) labels, predicted domains, and slice identity. Each row corresponds to a method: MaskGraphene, PRECAST, STAligner, DeepST, SPIRAL, GraphST, SpaDo, and SpaMask. **b** UMAP visualizations of joint embeddings generated by different methods for the MHypo five-slice integration (Bregma -0.04 - -0.24). Each row corresponds to a method: MaskGraphene, PRECAST, STAligner, DeepST, SPIRAL, GraphST, and SpaMask

In the previous section, we evaluated the quality of joint embeddings through pairwise evaluations of slice alignment and spot mapping. Expanding on this analysis, we further evaluated the integration quality of the “batch-corrected” joint embeddings generated by MaskGraphene using uniform manifold approximation (UMAP) visualizations for both DLPFC and MHypo slices. These evaluations were conducted in both pairwise and multi-slice scenarios for each dataset, comparing MaskGraphene with benchmarked methods. Each UMAP visualization is color-coded based on ground truth (GT) annotations, predicted domains, and the source of origin, shown in left, middle, and right panels, respectively, for each tool (Fig. 4).

Starting with the DLPFC 151675-151676 pair, UMAP plots for all tools showed that spots from the two slices were evenly mixed (Fig. 4a, right panels), and their predicted domain clusters were generally well segregated (Fig. 4a, middle panels). However, the concordance with the ground truth varied across tools (Fig. 4a, middle panels vs. left panels). Specifically, PRECAST tended to generate embeddings with highly separated clusters, often sacrificing geometric information. This led to predicted clusters that encompassed spots from different domains, a pattern that did not align well with the ground truth. SpaDo displayed the worst integration pattern. In contrast, tools such as MaskGraphene, STAligner, DeepST, SPIRAL, GraphST, and SpaMask preserved the hierarchical connections of the seven layers in the latent embedding space to varying degrees. These tools occasionally predicted spatial domains that included a few spots from nearby domains or split a single spatial domain into two adjacent ones. Notably, MaskGraphene demonstrated a unique ability to recover the entire slice shape, layer-wise patterns, and spatial relationships in the UMAP visualization. Overall, MaskGraphene produced the most visually coherent UMAP results among all tools, effectively mitigating batch effects and demonstrating superior integration quality. For the four-slice integration scenario (DLPFC 151673-151674-151675-151676) shown in Additional file 1: Fig. S8), similar UMAP visualizations and trends were observed across all tools. MaskGraphene continued to produce the most visually coherent UMAP results among all methods, although the overall UMAP outline was slightly deformed compared to the pairwise integration case. Similar UMAP visualization patterns were also observed in other pairwise and multi-slice scenarios, as demonstrated in Additional file 1: Fig. S9.

We then performed similar UMAP analysis on the MHypo dataset for both pairwise and five-slice integration scenarios. As shown in Fig. 4b and Additional file 1: Fig. S10, MaskGraphene demonstrated exceptional integration with batch correction compared to the other six tools. Its predicted domain clusters were well segregated and highly concordant with the ground truth (middle panels vs. left panels). The joint embeddings generated by MaskGraphene retained some degree of the original geometric information, although this effect was less pronounced compared to the DLPFC data. In contrast, the other six tools displayed significantly poorer integration performance relative to the ground truth, particularly in the four-slice integration scenarios (Fig. 4b).

**Fig. 5:**
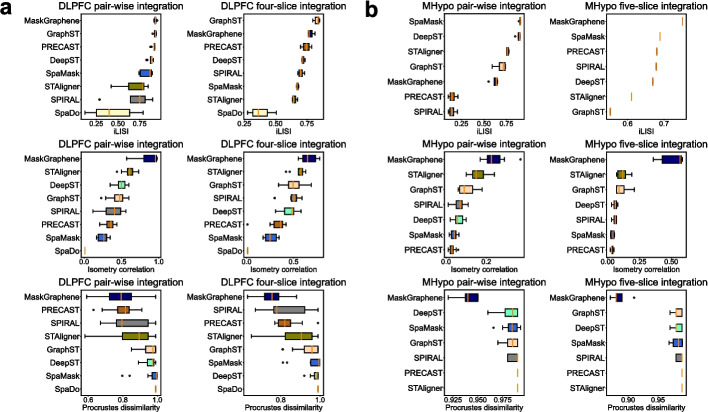
Box plots for iLISI, Isometry correlation, and Procrustes dissimilarity on the DLPFC and MHypo datasets. **a** Box plots representing iLISI, Isometry correlation, and Procrustes dissimilarity scores for different integration methods across all DLPFC pairwise and four-slice integrations. **b** Box plots representing iLISI, Isometry correlation, and Procrustes dissimilarity scores for different integration methods across all MHypo pairwise and five-slice integrations. BASS can not generate embeddings for UMAP visualizations, while SpaDo is incompatible with the MHypo dataset

To further quantitatively assess the batch effect removal and geometry preservation in the embeddings, we employed three metrics, as shown in Fig. 5. Detailed descriptions of these metrics are included in the Methods section. Batch effect removal across different methods was quantified using iLISI, where MaskGraphene achieved the highest scores in pairwise integration scenario and the second-highest in four-slice integration scenario for the DLPFC dataset, reflecting its ability to maintain well-mixed embedding across slices. Furthermore, MaskGraphene achieved the best performance in both pairwise and four-slice integration scenarios based on two geometry-preservation metrics: Isometry correlation and Procrustes dissimilarity. A higher Isometry correlation score indicates better geometry preservation, while a lower Procrustes dissimilarity score reflects superior geometry preservation. These findings align with the advanced alignment and mapping accuracy achieved by joint embeddings of MaskGraphene, further validating its capability to recover geometric shapes, spatial patterns, and layer-wise structures across various DLPFC slice integration scenarios. For the MHypo dataset, we found that MaskGraphene achieved the best scores across all integration scenarios, with the exception of the iLISI score in the pairwise integration scenario.

For both the DLPFC and MHypo datasets, we also evaluated the joint embeddings generated by MaskGraphene-PASTE. The results showed that while replacing MaskGraphene’s local alignment module with PASTE could still preserve the underlying geometric structure, the overall embedding-level performance was diminished compared to the original MaskGraphene (Additional file 1: Fig. S11). Furthermore, ablation studies across both datasets and all integration settings (pairwise and multi-slice) showed that MaskGraphene, the full model, achieved overall robust and top-tier performance in isometry correlation and Procrustes dissimilarity (Additional file 1: Fig. S12), indicating superior spatial fidelity and structural preservation. While the MaskGraphene-GAE variant performed relatively well in iLISI across integration scenarios, its performance in preserving geometric structure declined markedly. These results highlighted that the full model - combining hard-links, soft-links, and all loss functions - offered complementary advantages, resulting in improved local alignment and structural integrity.

**Fig. 6:**
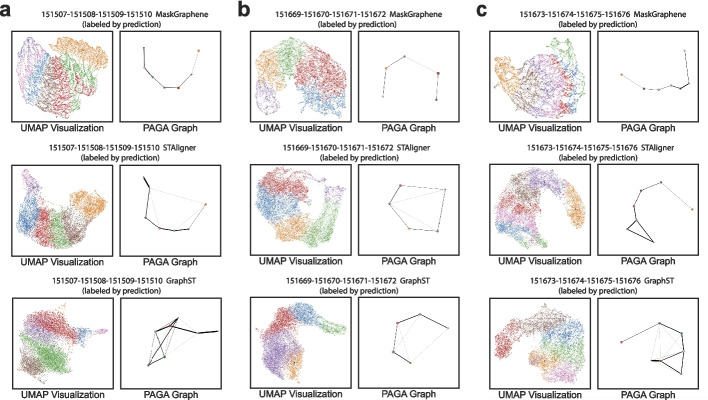
Visualization of UMAP paired with PAGA graphs on the DLPFC dataset. **a**-**c** Each panel shows UMAP visualizations paired with PAGA graphs, illustrating spatial trajectory results for three distinct DLPFC four-slice integration: (151507-151508-151509-151510) (**a**), (151669-151670-151671-151672) (**b**), and (151673-151674-151675-151676) (**c**). Spots are colored according to predicted domains. Each row corresponds to a method: MaskGraphene, STAligner, and GraphST

MaskGraphene effectively revealed spatial domains and boundaries by projecting joint embeddings onto a two-dimensional space using UMAP visualizations on the DLPFC dataset. We thus further inferred spatial trajectories with the trajectory inference tool PAGA to benchmark the quality of the joint embeddings. In Fig. 6, we present PAGA graphs illustrating connectivity patterns among spatial domains after four-slice integration using MaskGraphene, STAligner, and GraphST. We selected STAligner, and GraphST due to their relatively strong integration performance. Each set of two figure panels displays UMAP visualizations alongside PAGA graphs, with slices labeled by predicted clusters (Fig. 6). Across all three different four-slice integration scenarios, the PAGA graphs and UMAP plots generated by MaskGraphene’s embeddings showed that clusters corresponding to each layer were distributed accurately and exhibited the most consistent spatial trajectory from layer 1 to layer 6 and white matter (WM), indicating a linearly connected developmental trend. In contrast, STAligner demonstrated moderate connectivity, with some mixed connections in the middle layers, but struggled to maintain consistency. GraphST produced fragmented PAGA graphs, highlighting its limitations in preserving spatial domain continuity.

In summary, the results suggest that MaskGraphene’s batch-corrected joint embeddings, achieved through pairwise and multi-slice integration, effectively preserve the original geometric structure and accurately reconstruct spatial trajectory. Although other methods demonstrate competitive performance, they fall short of achieving the same level of robustness and precision. These findings highlight MaskGraphene’s capability to produce interpretable joint embedding in spatial transcriptomics, facilitating robust integration and efficient batch effect correction.

### MaskGraphene uncovers tissue topography through interpretable joint embeddings

Next, we evaluated whether MaskGraphene’s joint embeddings could reconstruct a topographic map of brain slices, capturing gradients of neuronal differentiation and activity. We employed GASTON [3] to analyze the topography of brain tissue slices. By integrating PCA-derived spot embeddings with spatial information, GASTON can characterize the topography of individual tissue slices. To assess whether joint embeddings from integration provide improved topographical representations and facilitate downstream gene expression gradient analyses compared to single-slice analysis, we utilized MaskGraphene’s joint embeddings as GASTON’s input. Additionally, to evaluate the extent to which two-dimensional UMAP coordinates preserve the original geometric structure, we replaced the actual X,Y coordinates with UMAP coordinates as input. To benchmark performance, we compared MaskGraphene against established tools such as STAligner and GraphST under various control settings, including GASTON’s default single-slice configuration.

**Fig. 7:**
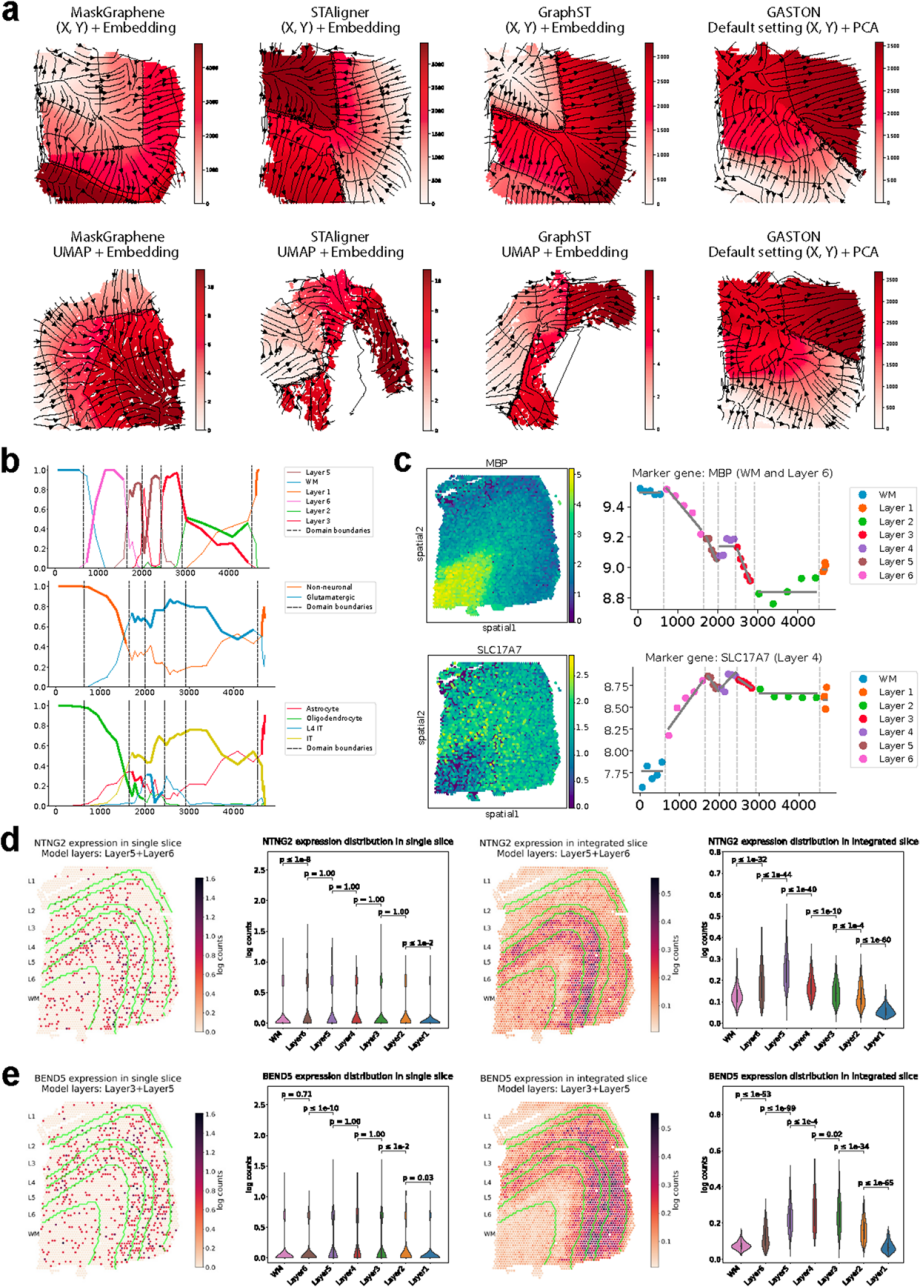
Biomarker and topography analysis after brain slice integration. **a** Top panels: Topographical maps generated using GASTON with four settings: original X,Y coordinates combined with joint embeddings after DLPFC 151673-151674 pairwise integration from MaskGraphene, STAligner, GraphST, and default PCA-derived embeddings from DLPFC slice 151673. Bottom panels: Maps generated with UMAP coordinates combined with embeddings from MaskGraphene, STAligner, GraphST, and default X,Y coordinates with PCA-derived embeddings from slice 151674. **b** Plots showing the proportions of cell types as a function of the isodepth, using three different types of annotations: layer-specific cell types (top panel), neuronal types (middle panel), and cell types (bottom panel). **c** Layer-specific marker gene analysis for MBP (white matter, layer 6) and SLC17A7 (layer 4). The left panels exhibit heatmaps of gene expression intensity, while the right panels depict the marker gene expression versus the isodepth. **d**-**e** Spatial expression patterns and layer-specific distributions of NTNG2 (**d**) and BEND5 (**e**) in single-slice (left) and integrated-slice (right) analyses. Green curves mark boundaries between cortical layers 1-6 (L1-L6) and white matter (WM). *P* values from two-sided Mann-Whitney U tests comparing distributions between adjacent layers are shown

As illustrated in Fig. 7a, the learned isodepth contour lines delineate a topographical map of the DLPFC slices, identifying the boundaries between distinct cortical layers. The spatial expression gradients, oriented perpendicular to these cortical layers (constant isodepth contours), indicate the directions of maximum gene expression change. By comparing all topographical maps by different settings, we found that GASTON, leveraging MaskGraphene’s joint embeddings from pairwise integration of DLFPC 151673-151674 pair along with X,Y coordinates, effectively segmented the tissue into several contiguous spatial domains that visually align with the layered structure of the DLPFC. Notably, this approach outperformed single-slice analysis with GASTON using default PCA-derived embeddings and X,Y coordinates. In contrast, when employing joint embeddings from STAligner or GraphST combined with X,Y coordinates, GASTON exhibited poor spatial organization of the DLPFC. Furthermore, when using two-dimensional UMAP coordinates derived from MaskGraphene’s embeddings in place of the original spatial context, we still observed a clear topographical map of the layered geometry of the DLPFC with well-defined continuous gradients. Conversely, GASTON failed to capture the topographical map when using the UMAP derived from STAligner and GraphST as input. These results further validates that MaskGraphene’s joint embeddings effectively capture the geometric structure, enabling robust downstream analyses.

We further compared the spatial domains (cortical layers) to the cell types reported in the original publication for this dataset. These cell types were obtained from the deconvolution module of SPACEL [33], which performs cell type deconvolution based on a reference scRNA-seq dataset. To conduct spatial domain-isodepth and cell type-isodepth analyses [3], we utilized the topographical map derived from integrating MaskGraphene’s joint embeddings with the original spatial context. Specifically, we computed isodepth coordinates to examine continuous variations in cell type composition across both integrated DLPFC slices and cortical layers. This analysis revealed significant variations in cell type proportions along the isodepth axis (Fig. 7b, middle and bottom panels), which aligned well with the reported cell type abundances and their functional roles within each cortical layer (Fig. 7b, top panel). For example, by aligning and analyzing the three panels together (Fig. 7b), we observed that a high abundance of oligodendrocytes across the isodepth range corresponding to white matter (WM) and the deeper cortical layers (layer 5 and 6). This finding is consistent with their critical role in myelinating the large, long-projecting axons of pyramidal neurons that connect the cortex to subcortical structures [40]. These oligodendrocytes also constitute a significant proportion of the non-neuronal cells in these layers. Furthermore, a sharp transition in cell-type proportions was observed at the isodepth value used as the boundary (second dashed line) between layers 5 and 6, indicating that the learned isodepth and spatial domains effectively separate non-neuronal cells from neurons. Notably, glutamatergic neurons, including intratelencephalic projection (IT) neurons, exhibited a large and nearly constant proportion across the isodepth range corresponding to layers 2-5, consistent with their role in excitatory signaling within the brain and their importance in various cognitive and motor functions [41]. These findings demonstrate that the topographical map generated using isodepth not only identifies more spatially coherent domains compared to existing methods but also preserves accurate cell type information.

Finally, we explored whether the learned topography based on integration, could facilitate the identification of biologically meaningful spatial patterns of gene expression. This analysis focused on capturing both continuous variations in expression within or across spatial domains and abrupt discontinuities in gene expression at the boundaries of adjacent spatial domains [3]. Details of the analysis are described in the Methods section. For example, in the DLPFC, the marker gene MBP (Myelin Basic Protein) is predominantly expressed in oligodendrocytes, the glial cells responsible for forming and maintaining myelin sheaths, essential for efficient nerve signal conduction [42]. Its expression peaked in the white matter (WM) and showed a continuous gradient across layers 5 and 6 along the isodepth axis (Fig. 7c, top panels). However, MBP expression exhibited sharp discontinuities at the boundaries of layer 2, reflecting its low abundance in the superficial layers. This gradient pattern aligned with results from the cell type-isodepth analysis. As another example, the layer 4 marker gene SLC17A7, also known as VGLUT1 (vesicular glutamate transporter 1), serves as a key marker for excitatory glutamatergic neurons due to its role in glutamate signaling. This gene showed peak expression in layer 4, indicating a high density of these neurons, despite reduced visibility on the heatmap due to sparse expression values (Fig. 7c, bottom panels). This peak extended into layers 3 and 5, reflecting the laminar organization and enrichment of excitatory neurons in these regions [43].

We performed similar spatial domain-isodepth, cell type-isodepth, and marker gene gradient analyses using joint embeddings generated by STAligner, GraphST, and default PCA-derived embeddings. However, these methods failed to produce biologically meaningful results comparable to those achieved with MaskGraphene (Additional file 1: Figs. S13-15). Additionally, we extended the analyses using joint embeddings from MaskGraphene’s four-slice integration, which yielded results consistent with those observed in pairwise integration (Additional file 1: Fig. S16), but notably improved the smoothness and continuity of gene expression patterns. For example, both layer marker genes MBP and SLC17A7 displayed smoother and more continuous expression profiles under four-slice integration (Additional file 1: Fig. S16c) compared to pairwise integration (Fig. 7c). In contrast, when MaskGraphene’s embeddings were derived from a single slice (Additional file 1: Fig. S17), the cell type patterns were less smooth, and the gene expression gradients appeared more fragmented and misaligned with the laminar structure, further underscoring the robustness of MaskGraphene’s integrative topography.

### MaskGraphene enhances biomarker discovery via integrated analysis

To assess whether integration improves biomarker identification, we compared layer marker genes identified from individual slices based on ground truth labels with biomarkers identified from each integrated layer across four slices. First, we matched MaskGraphene’s predicted domains with ground truth labels to annotate the layers (Additional file 1: Fig. S18a) and extracted spots corresponding to each layer from all four slices. Biomarkers for each integrated layer were then identified using Scanpy [44]. Next, to generate ground truth layer marker genes for comparison, we used Scanpy to identify biomarkers for each layer in individual slices based on ground truth labels, selecting the top 50 or 100 genes per layer per slice. The union of these top genes, denoted as $N$, across all four slices was compiled as the final set of ground truth layer marker genes for validation. Finally, we calculated the overlap ratio between the top $N$ biomarkers identified for each integrated layer and the $N$ ground truth layer marker genes. MaskGraphene’s performance was benchmarked against STAligner and GraphST. As shown in Fig. Additional file 1: Fig. S18a, MaskGraphene’s predictions closely matched the ground truth, accurately capturing the spatial organization of these brain regions. Furthermore, MaskGraphene achieved higher overlap ratio across nearly all layers when compared to the ground truth marker genes, defined by selecting the top 50/100 layer marker genes (Additional file 1: Fig. S18b). This performance exceeded that of STAligner and GraphST, particularly in layers 1 and 3, where MaskGraphene exhibited significantly higher overlap ratios. These results highlight MaskGraphene’s enhanced accuracy in identifying and localizing these domains after integration, thereby enhancing biomarker identification.

We also applied GASTON to identify spatial gene expression peaks under three conditions: (1) the integrated slices 151673 and 151674 processed by MaskGraphene, (2) single slice 151673, and (3) single slice 151674. Marker genes identified under each condition were cross-referenced with a curated list of canonical DLPFC layer markers and their annotated layer identities derived from prior studies [39, 45], to assess whether their spatial expression patterns aligned with known cortical laminar structures. Our results showed that MaskGraphene’s integrated output enabled the detection of 18 additional layer-consistent marker genes that were not identified in the union of the two individual slice analyses. This suggests that MaskGraphene effectively leverages integration to enhance biological signal recovery and improve sensitivity in detecting layer marker genes. To visualize these results, we adopted the spatial gene expression plotting approach described in Zeira et al.’s study [31], using MaskGraphene’s alignment. As illustrated in Fig. 7d-e, the additional markers identified through integration (e.g., NTNG2 and BEND5) exhibited distinct, layer-specific expression patterns that aligned with established cortical layers, while their expression in individual slices appeared sparse and lacked clear laminar separation. These results reinforce the biological validity of the integrated embedding and demonstrate that MaskGraphene enables more comprehensive and meaningful marker discovery across spatial slices.

We next assessed whether integration improves biomarker identification in a breast cancer dataset. Consistent with our observations in the DLPFC dataset, MaskGraphene’s predictions closely matched the ground truth, accurately delineating the spatial organization of healthy and tumor-associated regions. Among all methods, MaskGraphene achieved the highest ARI (0.56), followed by GraphST (0.54) and STAligner (0.50). Furthermore, MaskGraphene yielded higher overlap ratios across nearly all domains when compared with ground truth marker genes defined by the top 50/100 layer markers (Additional file 1: Fig. S19a-b), outperforming both STAligner and GraphST in nearly every domain. Importantly, integration uncovered genes ranked among the top 100 markers per cluster in the integrated dataset but absent from both individual slice analyses, highlighting MaskGraphene’s ability to recover unique markers detectable only through pairwise integration. For example, as shown in Additional file 1: Fig. S19c, KRT5 emerged as a top marker with strong, spatially coherent expression along tumor edge 3, whereas its expression appeared sparse in individual slices. KRT5 is a well-established basal/myoepithelial marker in breast cancer, enriched in basal-like and triple-negative subtypes, where its upregulation has been associated with aggressive phenotypes, chemoresistance, and poorer survival outcomes [46]. Other top marker genes, including CCL21, CARTPT, and SPP1, also displayed stronger and more localized expression patterns in their respective domains after integration compared to individual slice analyses.

### MaskGraphene successfully captures the partial-overlap slice phenomenon in simulated data

**Fig. 8:**
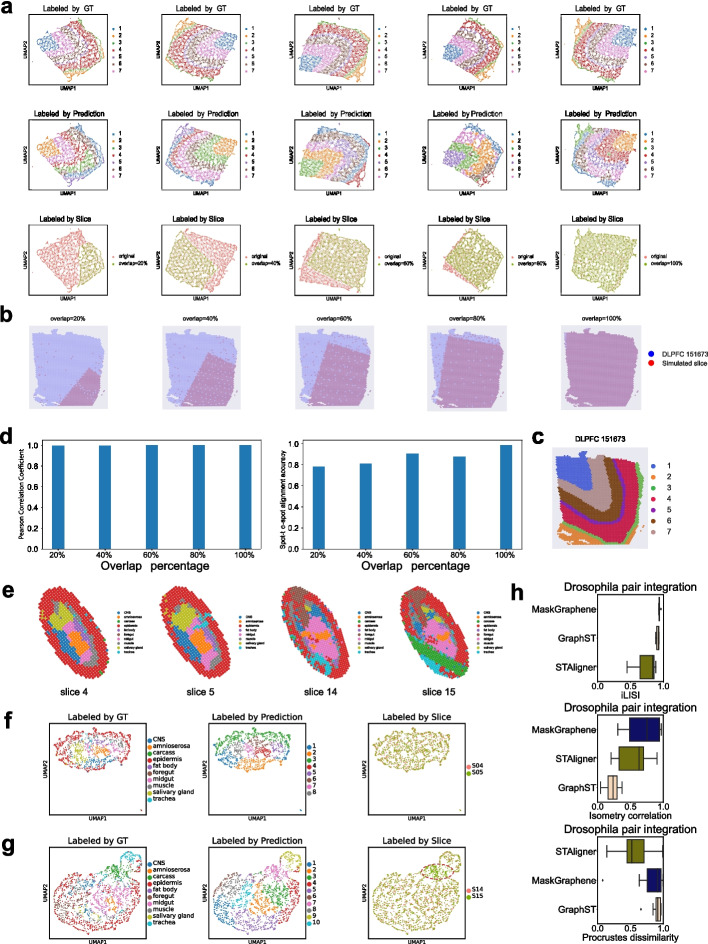
UMAP visualization and validation plots for simulated data integration with varying partial overlap percentages. **a** UMAP visualizations labeled by ground truth (GT) labels, predicted domains, and slice identity. Each column represents a specific overlap percentage scenario (20%, 40%, 60%, 80%, and 100%, from left to right). **b**-**c** DLPFC 151673 slice with seven layers, accompanied by simulated consecutive slices showing overlap ratios of 20%, 40%, 60%, 80%, and 100% relative to the reference slice. **d** The left panel represents the Pearson correlation between joint embeddings of aligned spots across the reference and simulated slices as the overlap ratio increases. The right panel indicates the spot-to-spot alignment accuracy as the overlap ratio increases. **e** Spatial visualization of annotated tissue domains for Stereo-seq Drosophila slices (S04, S05, S14, S15), colored by ground-truth labels. **f**-**g** UMAP visualizations of joint embeddings from pairwise integrations of S04-S05 (**f**) and S14-15 (**g**). Spots are colored by ground truth labels (left), model predictions (middle), and slice identity (right). The partially overlapping region in (**g**) is highlighted by a dashed red circle. **h** Quantitative integration metrics (iLISI, Isometry correlation, and Procrustes dissimilarity) for all Drosophila pairwise integrations, comparing MaskGraphene, GraphST, and STAligner

While real datasets provided some insight into alignment accuracy and integration, they lacked precise spot-to-spot alignment ground truth. To thoroughly investigate alignment accuracy and integration, we simulated datasets with a gold standard for different scenarios to demonstrate the robustness of MaskGraphene (Fig. 8). In order to explore capturing different overlap geometric structures in the latent space, we conducted simulations using one DLPFC slice as the reference and generated another slice with varying overlap ratios (20%, 40%, 60%, 80%, and 100%) compared to the reference slice (Fig. 8b-c). Further details about the data simulation are provided in the Methods section. This allowed us to examine how the joint embeddings by MaskGraphene could accurately represent these variations. The resulting joint embeddings, obtained by integrating the reference slice with each simulated slice, were visualized in the two-dimensional UMAP space. Spots in the visualization were color-coded based on ground truth (GT) annotations, predicted domains, and the source of origin (Fig. 8a).

We noticed that the spots from two different slices were evenly distributed while the predicted cortical layers were clearly separated, showing a strong agreement with the ground truth (GT) (Fig. 8a, top panels vs. middle panels). The UMAP visualizations preserved the geometric structure of the DLPFC slices, which aligns with our previous observations. Remarkably, the slices labeled by their source effectively depicted the gradually increasing patterns of overlap, showing varying proportions of red spots exclusively associated with the reference slice, as highlighted in the original reference-to-simulated slices (Fig. 8a, bottom panels vs. Fig. 8b). We computed the Pearson correlation coefficient between joint embeddings of aligned spots based on GT and the spot-to-spot alignment accuracy for all five overlap scenarios. As depicted in Fig. 8d, the Pearson correlation reached 1.0 for all scenarios, indicating robust embeddings for alignment and integration. The spot-to-spot alignment accuracy achieved 100% when two slices had 100% overlap, and this accuracy slightly decreased as the overlap ratio decreased. This analysis demonstrated that MaskGraphene is capable of integrating partial overlap slices while preserving the underlying geometric structure of the overlaps.

We further applied MaskGraphene to a Stereo-seq dataset [47] generated from late-stage Drosophila embryos (14-16 hours after egg laying, labeled E14-16). This dataset includes 16 slices, each with approximately 1,000 spots. Notably, slices from the same stage differ in size and cell type composition and do not fully overlap spatially, making this a representative real-world scenario for evaluating the robustness of integration methods under partial overlap. In the original study, cell types were assigned to each spot through unsupervised clustering of gene expression profiles, followed by marker gene-based annotation. As shown in Fig. 8f, for the pairwise integration of slices S04 and S05, MaskGraphene effectively preserved the overall shape of the embryo as well as the relative spatial arrangement of its internal tissues, while also achieving batch correction. These joint embeddings closely aligned with the GT labels and the known anatomical organization of the tissues (Fig. 8e vs. f). We further evaluated a challenging case involving slices S14 and S15, which exhibit clear differences in cell type composition, with carcass cells showing the most pronounced difference in abundance (Fig. 8e). As shown in Fig. 8g, MaskGraphene successfully distinguished the carcass cells from slice 15 (highlighted by the dashed red circle), while preserving the embryo’s geometric integrity. For comparison, we benchmarked STAligner and GraphST on all pairwise integrations in this dataset. While both methods were able to identify the carcass cells in slice 15, they failed to preserve the global geometric structure of the embryo and showed inferior qualitative performance (Fig. 8h and Additional file 1: Fig. S20). These results highlight MaskGraphene’s ability to integrate partially overlapping real-world ST slices with both biological accuracy and geometric fidelity.

### MaskGraphene enhances domain identification through joint embedding

By integrating data from multiple ST slices, we can estimate joint embeddings of expressions that represent variations between cell or domain types across slices. This approach has the potential to enhance the detection of spatial domains or cell types. To further quantitatively compare the effectiveness of MaskGraphene in capturing spatial domains via joint embeddings, we utilized joint embeddings from pairwise and multi-slice integration in the MHypo and DLPFC datasets to perform clustering using the clustering method mclust [48]. Subsequently, we calculated the Adjusted Rand Index (ARI) as an evaluation metric to compare the clustering results of MaskGraphene with the ground truth in each slice, with higher ARI scores indicating better domain identification. We benchmarked MaskGraphene against all seven integration methods. BASS was included in this analysis as it provides clustering labels after integration rather than joint embeddings [16, 23].

**Fig. 9:**
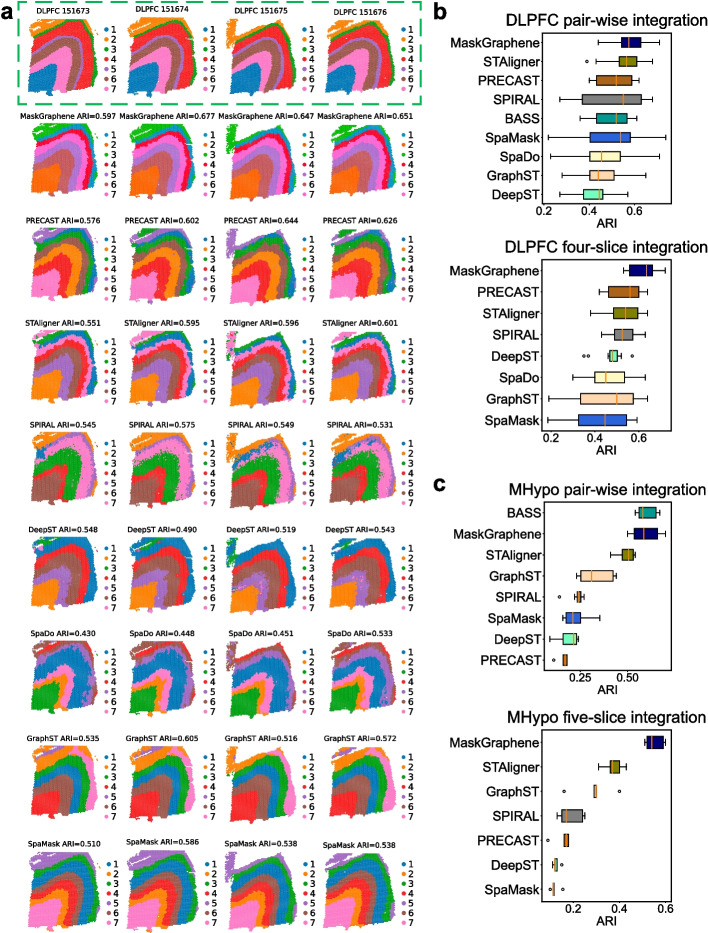
Spatial visualization of joint domain identification and ARI boxplots. **a** Spatial domain identification visualization with ARI value for four DLPFC slices (151673, 151674, 151675, and 151676) after integration using eight integration methods, including MaskGraphene, PRECAST, STAligner, SPIRAL, DeepST, SpaDo, GraphST, and SpaMask. The first row, outlined by a green dashed box, represents the ground truth labels. **b** Box plots showing ARI scores for clustering results after all DLPFC pairwise integration (top panel) and four-slice integration (bottom panel) across all methods. Tools are sorted by average ARI. **c** Box plot showing ARI scores for all MHypo pairwise integration (top panel) and the five-slice integration (bottom panel). BASS is not suitable for multi-slice integration, while SpaDo is incompatible with the MHypo dataset

In Fig. 9a, we visualized domain identification with the ARI score on each slice for the DLPFC four-slice integration (151673-151674-151675-151676). MaskGraphene demonstrated a distinct separation of the seven-layered regions and achieved the highest clustering accuracy across all four slices. In contrast, most other tools struggled to reveal the expected layer pattern consistently in all slices. Their clustering results often displayed chaotic cluster boundaries and numerous outliers within each cluster, which compromised both the overall clustering accuracy and the visual clarity of the results. Additional visualizations of domain identification with ARI scores for other integration scenarios are provided in Additional file 1: Figs. S21-24. To demonstrate the robustness of clustering performance, we plotted ARI scores across all pairwise and four-slice integration scenarios using box plots. As shown in Fig. 9b, MaskGraphene achieved the best overall clustering performance using joint embeddings from both pairwise and four-slice integration, followed by STAligner and PRECAST.

For the MHypo dataset, as shown in Fig. 9c, BASS and MaskGraphene achieved the highest ARI scores, highlighting their superior performance in domain identification after pairwise integration. In the five-slice integration scenario, MaskGraphene achieved the highest ARI score, followed by STAligner and GraphST, which showed moderate performance. Additional visualizations of spatial domain identification with ARI scores for this dataset are available in Additional file 1: Figs. S25-26.

As outlined in the Methods section, users can employ either a coordinate replacement or a coordinate transformation strategy for multi-slice integration (involving more than two slices) to unify the coordinate system when constructing the k-NN graph. For the DLPFC and MHypo datasets, we primarily demonstrated results obtained using MaskGraphene with coordinate replacement (the default strategy). Additionally, results generated using MaskGraphene with coordinate transformation are presented in Additional file 1: Figs. S27-28. These results demonstrate that MaskGraphene (coordinate transformation) performs comparably to MaskGraphene (coordinate replacement) across analyses such as UMAP visualization, spatial trajectory, biomarker identification, and spatial domain identification.

### MaskGraphene facilitates the integration of numerous adjacent consecutive ST slices

**Fig. 10:**
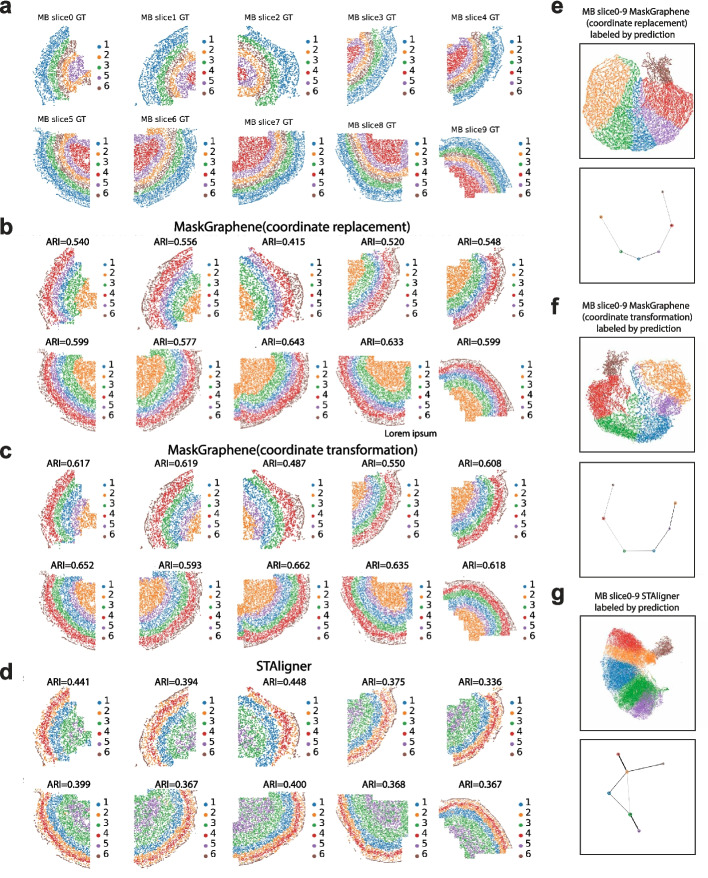
Spatial visualizations of joint domain identification and UMAP paired with PAGA plots for the integration of the MERFISH mouse brain (MB) dataset. **a** Spatial domain identification visualization for MB slices (0 - 10) by MaskGraphene and STAligner. Each pair of rows corresponds to a different method or ground truth (GT). **b** UMAP visualizations paired with PAGA graphs to assess spatial trajectory results, with UMAP spots colored by predicted domains or GT labels

In this section, we evaluated MaskGraphene’s ability to integrate a substantial number of adjacent consecutive ST slices for large-scale analysis. Specifically, we utilized ten consecutive tissue slices from the primary motor cortex of a mouse embryo (MB) MERFISH dataset [33, 49] to perform integration and generate joint embeddings for clustering performance and trajectory inference analysis. Region annotations, including the six layers (L1-L6) and white matter (WM), are provided for each slice for benchmarking. All the other methods, except for STAligner, encountered GPU memory constraints or other issues that prevented them from completing this analysis. In Fig. 10a-d, we present the visualization of domain identification, along with the ARI scores for each slice in the MB ten-slice integration. Both MaskGraphene (coordinate replacement) and MaskGraphene (coordinate transformation) consistently outperformed STAligner across nearly all slices, accurately identifying distinct cortical layers with well-defined boundaries as indicated by the ground truth. The high ARI scores achieved by MaskGraphene across all slices (ranging from 0.415 to 0.662) demonstrate its robust and reliable clustering performance, even as the number of integrated slices increased. Overall, MaskGraphene (coordinate transformation) achieved better clustering performance than MaskGraphene (coordinate replacement). In contrast, STAligner exhibited lower ARI scores (ranging from 0.336 to 0.448) and less consistent clustering patterns across all slices. This disparity became more pronounced with an increasing number of slices, indicating that STAligner may struggle to integrate large ST datasets while maintaining clustering accuracy.

The UMAP visualizations (Fig. 10e-f, top panels and Additional file 1: Fig. S29) revealed well-distributed clusters corresponding to each layer, displaying a clear hierarchical layer structure with spots from all slices evenly mixed. These results indicate that MaskGraphene effectively harmonized data across numerous slices, successfully mitigating potential batch effects and inter-slice variability that might otherwise compromise integration. The corresponding PAGA graphs further highlighted a consistent spatial trajectory from layer 1 to layer 6, based on the domains predicted by both MaskGraphene (coordinate replacement) and MaskGraphene (coordinate transformation) (Fig. 10e-f, bottom panels). In contrast, PAGA graphs derived from joint embeddings of STAligner showed misconnections across several predicted layers, failing to capture a linearly connected developmental progression. In summary, despite the challenges associated with integrating numerous adjacent consecutive ST slices, MaskGraphene successfully achieved effective integration. It generated joint embeddings that captured the geometric structure to a significant extent, revealed the spatial trajectory, and maintained strong clustering performance across individual slices.

### MaskGraphene stitches mouse brain anterior and posterior sections

**Fig. 11:**
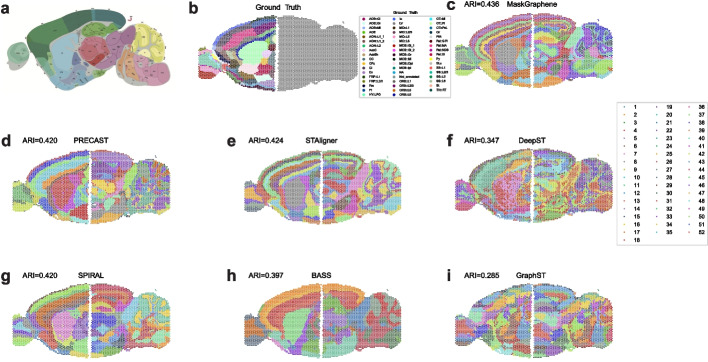
Spatial visualization plots for integration of the dataset of mouse brain sagittal sections. **a** The Allen brain atlas annotation for the mouse brain sagittal section. **b** Ground truth annotation of different regions for the anterior slice. **c**-**i** Domain identification using seven different methods on the mouse brain sagittal dataset, with ARI score evaluated for the anterior slice

Thus far, we have examined MaskGraphene’s capability to integrate adjacent consecutive tissue slices. In this section, we extended our investigation to evaluate its performance in integrating horizontally consecutive slices, focusing on two 10x Visium mouse brain sagittal sections divided into anterior and posterior portions (MB2SA&P) [13, 50]. To evaluate the integration effect, we employed the Allen Brain Atlas as reference (Fig. 11a), enabling visual comparisons of MaskGraphene’s clustering results against the other six methods (Fig. 11c-i). SpaMask is not applicable in this integration scenario. PRECAST, SPIRAL, DeepST, and GraphST were not able to detect and connect common spatial domains along the shared boundary of the anterior and posterior sections. In contrast, MaskGraphene, STAligner, and BASS successfully identified and linked common spatial domains along the shared boundary. Notably, MaskGraphene and STAligner excelled in the cerebral cortex (CTX) region, where they outperformed the other tools by accurately identifying and aligning six distinct layers across the anterior and posterior sections. Moreover, for unshared regions, both MaskGraphene and STAligner excelled in separating the caudal putamen (CP) and nucleus accumbens (ACB) and distinguishing the layers within the cerebellar cortex (CBX). Additionally, both methods effectively identified a coherent arc across two sections for CA1, CA2, and CA3. Using ground truth annotations from the anterior region (Fig. 11b), we further quantified the integration performance by calculating the ARI score based on joint embeddings. MaskGraphene achieved the highest ARI score of 0.436, closely followed by STAligner (0.424). These results highlight the performance of MaskGraphene and STAligner in capturing spatial structures within complex datasets and their proficiency in integrating non-consecutive slices with batch correction.

### MaskGraphene aligns tissues and organs across different developmental stages

**Fig. 12:**
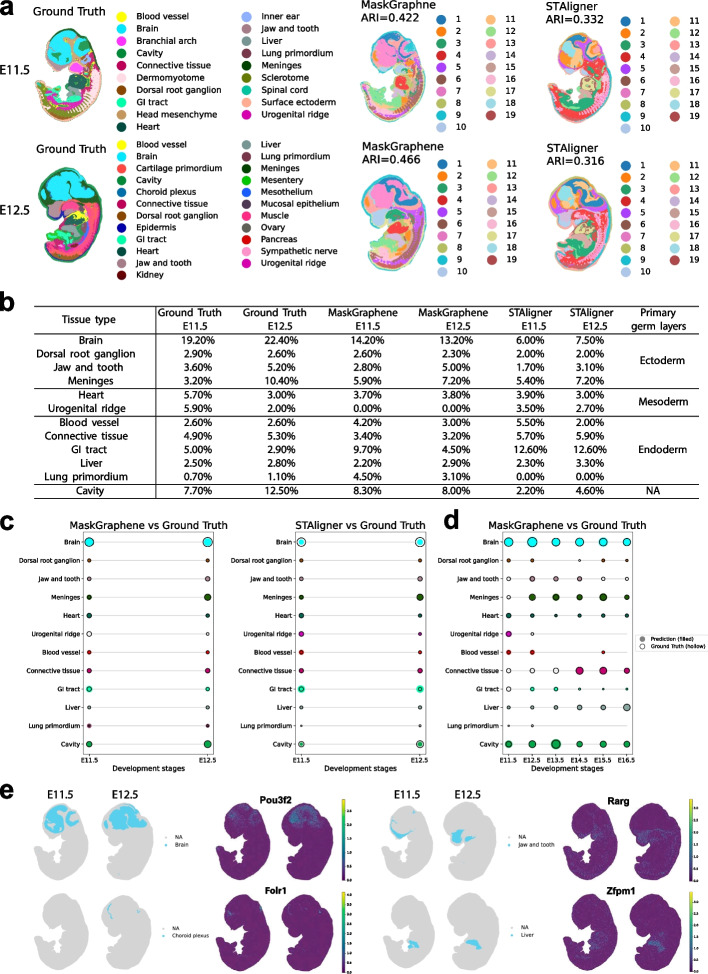
Developmental progression evaluation of spatial domain integration across embryonic developmental stages. **a** Spatial visualization of mouse embryos at stages E11.5 and E12.5, with ARI indicated. **b** Proportion of spots for each structure relative to the total spots at stages E11.5 and E12.5, based on the ground truth and the integration results from MaskGraphene and STAligner. **c** Visual comparison of the tissue proportions in (**b**) for MaskGraphene and STAligner versus ground truth at stages E11.5 and E12.5, with filled circles indicating predicted proportions and hollow circles indicating ground-truth proportions. **d** Visual comparison of tissue proportions for MaskGraphene and STAligner versus ground truth across six stages (E11.5-E16.5), with filled circles indicating predicted proportions and hollow circles indicating ground-truth proportions. **e** Spatial mapping of representative tissue predictions (ground truth) alongside expression patterns of four example top marker genes (Pou3f2, Folr1, Rarg, and Zfpm1) from the integrated analysis, illustrating the correspondence between predicted domains and gene expression enrichment in individual slices

In this section, we tested MaskGraphene’s ability to integrate multiple slices from different development stages for joint analysis, to study the spatiotemporal development in tissue structures during mouse organogenesis. Using Stereo-seq data from two mouse embryo slices acquired at different time points (E11.5 and E12.5) [15], we first performed a pairwise integration analysis, benchmarking MaskGraphene against STAligner. Other tools were excluded from the comparison due to memory limitations with this large dataset. As shown in Fig. 12a, despite differences in the sizes of the two slices and the presence of noticeable batch effects, both MaskGraphene and STAligner effectively harmonized the data by integrating them into a unified embedding space. They accurately detected both shared (labeled consistently across slices by the two tools and the ground truth) and developing structures unique to different time points. To evaluate the clustering performance of joint embeddings, we calculated ARI scores by comparing the detected clusters against annotated ground truth labels. MaskGraphene outperformed STAligner, achieving higher ARI scores of 0.422 and 0.462 for the two slices compared to STAligner’s scores of 0.332 and 0.316.

To assess the temporal development of each tissue structure during mouse organogenesis, we quantitatively analyzed the sizes of shared tissue structures across two time points by calculating the proportion of spots corresponding to each tissue structure relative to the total number of spots at each time point. For benchmarking purposes, we also computed these proportions using ground truth annotations. MaskGraphene demonstrated closer alignment with the ground truth proportions across both time points compared to STAligner. To illustrate these results, we grouped all shared tissue structures into three primary germ layers and displayed the proportions for each tool alongside the ground truth (Fig. 12b-c). Additionally, we calculated the proportional differences across these tissue structures between each tool and the ground truth. MaskGraphene achieved a smaller difference of 45.7% and 36.3%, compared to STAligner’s 62.8% and 57.6% for E11.5 and E12.5, respectively (Additional file 1: Table S2). Notably, the brain, which constitutes the largest proportion of the embryo at both time points, was largely undetected by STAligner, which also missed the lung primordium (Fig. 12b-c). In contrast, MaskGraphene successfully detected most of the brain but failed to identify the urogenital ridge (Fig. 12b-c). Furthermore, MaskGraphene captured the actual proportions and temporal changes from E11.5 to E12.5 more accurately for a broader range of tissue types, including the dorsal root ganglion, jaw and tooth, and liver (Fig. 12b-c). We further extended this analysis to include four more later developmental stages (E13.5, E14.5, E15.5, and E16.5). Due to the large size of the dataset (496,494 total spots), STAligner could not perform integration at this scale, so we included results from MaskGraphene only. As shown in Fig. 12d, MaskGraphene accurately captured tissue-type proportions and temporal dynamics from E11.5 to E16.5 across most tissue structures. Notably, it shows a consistently large brain size from E11.5 to E16.5 with a slight relative decline over time - consistent with the literature noting early brain growth followed by more proportional development of other systems [51, 52]. The liver shows a clearly increasing trend, in line with reports that it becomes one of the most enlarged organs during late organogenesis [53, 54]. MaskGraphene also correctly reflected the absence of certain tissues, such as blood vessels at E13.5, E14.5, and E16.5, consistent with the ground truth. This matches known patterns of blood vessels expanding early but stabilizing or shrinking in relative size as solid tissues grow [55]. However, some tissues were missed, including connective tissue at E11.5-E13.5 and jaw and tooth regions at E15.5-E16.5. These results facilitated the reconstruction of the developmental progression of each tissue structure throughout organogenesis.

Next, we explored whether integration analysis improves biomarker identification for these shared tissue structures. Six key structures were selected: blood vessel, brain, dorsal root ganglion, heart, jaw and tooth, and liver. To assess this, we compared the top $N$ marker genes from individual slices, based on ground truth labels, with biomarkers identified from the integrated tissue structures across two embryo slices (E11.5 and E12.5). Details of our approach are provided in the Methods section. MaskGraphene consistently achieved higher overlap ratios across nearly all tissue structures when compared to the ground truth marker genes, defined by selecting the top 50/100 tissue marker genes (Additional file 1: Fig. S30). Notably, its performance exceeded that of STAligner, especially in the brain, where MaskGraphene exhibited significantly greater overlap ratios. These results aligned with the temporal development analysis. We also examined genes that ranked among the top 100 markers per cluster in the integrated Embryo dataset but were absent from both individual time point analyses. This approach enabled the identification of uniquely recovered markers detectable only through integration, demonstrating how MaskGraphene leverages complementary spatial and transcriptional patterns across slices to enhance biological signal recovery. In brain regions, for example, we recovered canonical neurogenic regulators such as Pou3f2, Ascl1, and Neurog2 - genes not highly ranked in either single-slice analysis but well established for their roles in neuronal fate specification [56, 57]. In the choroid plexus, integration revealed Folr1, Cetn2, and Sp5, consistent with known ciliary functions and cerebrospinal fluid production in this epithelial niche [58]. Similarly, in craniofacial clusters associated with jaw and tooth development, markers such as Rarg and Atrx emerged [59, 60], while in the fetal liver, we observed enrichment of metabolic regulators like Zfpm1 and Ahsg, reflecting stage-specific hepatic functions [61]. As shown in Fig. 12e and Additional file 1: Fig. S31, these genes exhibited either sparse expression or lacked clear tissue-specific localization in individual slices. Integration effectively aggregated their signals, enabling their identification as spatially informative biomarkers. These examples illustrate how joint integration can amplify subtle, distributed expression signals that may fall below detection thresholds when slices are analyzed individually. We also note that some tissue regions - such as the cartilage primordium and surface ectoderm - showed few or no uniquely recovered markers for integration analysis. This may reflect either a lower benefit from integration in these domains - perhaps due to high inter-slice consistency.

In summary, the results confirm that MaskGraphene is superior in preserving spatial domains, maintaining stage-to-stage consistency, accurately representing tissue proportions across embryonic stages to some extent, and enhancing biomarker identification. These strengths are particularly valuable for developmental studies, where accurate tissue identification and cross-stage consistency are crucial.

### Runtime efficiency

Finally, we evaluated the runtime of all methods under two integration scenarios (pairwise and multi-slice) across multiple datasets. As shown in Additional file 1: Fig. S32, in the pairwise integration scenario (Fig. S32a), MaskGraphene consistently demonstrated low runtimes across diverse datasets (DLPFC, MHypo, MB2SA&P, and mouse embryo), often outperforming other methods by a substantial margin. DeepST and SPIRAL exhibited the highest runtimes, particularly for the MHypo dataset, where execution time exceeded $10^4$ seconds. In the multi-slice integration scenario (Fig. S32b), MaskGraphene maintained strong scalability, delivering favorable runtimes even for large datasets such as the mouse embryo with 500k spots. While some methods (e.g., GraphST, PRECAST) performed comparably on smaller datasets, MaskGraphene provided the most consistent balance of efficiency and scalability across all tested datasets. These results highlight the suitability of MaskGraphene for both small- and large-scale spatial transcriptomics integration tasks.

## Discussion

In this work, we introduce MaskGraphene, a graph neural network that integrates spatial transcriptomics data by jointly leveraging gene expression and spatial information. The workflow begins with an initial embedding learned by a masked graph attention autoencoder on intra-slice k-NN graphs, optimized with a masked self-supervised reconstruction loss and a triplet loss. The triplet loss, acting as indirect “soft-links”, progressively refines inter-slice connectivity and facilitates the identification of shared clusters. To further strengthen these connections, MaskGraphene performs cluster-wise local alignment between consecutive slices to establish direct spot-to-spot mappings (“hard-links”). These hard-links are then used to construct an inter-slice k-NN graph, which is processed again by the masked graph attention autoencoder to generate final joint embeddings that preserve geometric structure and enhance downstream analyses. We evaluated MaskGraphene against several state-of-the-art integration methods on diverse real and simulated datasets, using multiple downstream tasks and evaluation metrics. Across all benchmarks, MaskGraphene consistently achieved superior performance.

Existing clustering and integration tools face challenges in generating latent embeddings that effectively preserve the geometric information of original tissue slices. MaskGraphene addresses this gap, producing interpretable joint embeddings that faithfully capture and reflect the geometric structure of tissue slices. These embeddings integrate both gene expression and spatial context information to some extent, making them suitable replacements for these two modalities in various downstream analyses. For example, in our study, we utilized joint embeddings to construct a topographic map of brain slices, revealing gradients of neuronal differentiation and activity. However, the ability to generate interpretable joint embeddings is limited to certain application scenarios. For the DLPFC and Drosophila datasets, which exhibit layer structures across all adjacent consecutive slices, MaskGraphene’s joint embeddings effectively recover the overall slice shape, layer-wise patterns, and spatial relationships in two-dimensional UMAP space, capturing geometric information with near-perfect accuracy. In contrast, for more heterogeneous tissues, such as those in the MHypo dataset, capturing perfect geometric information becomes significantly more challenging. In the field of spatial transcriptomics, developing interpretable embeddings across all tissue slices remains a challenging yet important area of research. Such embeddings hold the potential to significantly enhance a broad spectrum of downstream analyses and provide valuable biological insights.

The core components of MaskGraphene - masked node feature reconstruction and cluster-wise local alignment - were first introduced in our bioRxiv preprint (February 2024) and presented at RECOMB-Seq (April 2024), marking one of the earliest applications of masked graph modeling to ST integration. A more recent tool, SpaMask, also adopts masked self-supervised learning but follows a different design strategy. SpaMask masks both graph edges and gene expression features during training and relies on external alignment tools (e.g., ICP [62] or PASTE) for preprocessing. In contrast, MaskGraphene integrates a cluster-wise OT-based local alignment module that directly learns cross-slice correspondences without pre-alignment and applies masking only in the feature space, supported by an auxiliary subnetwork on unmasked data to stabilize training and preserve topology. Although SpaMask performs competitively in certain scenarios (e.g., achieving higher iLISI scores for MHypo pairwise alignment), MaskGraphene delivers more consistent improvements in alignment accuracy, geometry preservation, and scalability. Together, these approaches underscore the growing role of masked self-supervised learning in advancing next-generation ST integration tools.

## Conclusions

MaskGraphene offers an interpretable and scalable framework for integrating spatial transcriptomics data across multiple slices and experimental conditions. By unifying optimal transport-based local alignment, masked self-supervised representation learning, and triplet-driven soft-link modeling, it generates joint embeddings that faithfully preserve spatial geometry while correcting batch effects. Extensive benchmarking demonstrates that MaskGraphene outperforms existing approaches in alignment accuracy, geometric fidelity, and domain identification, enabling robust reconstruction of spatial trajectories, topographical organization, and biomarker landscapes. Its ability to scale from pairwise to large multi-stage integrations highlights its broad applicability across diverse biological systems. Collectively, these advances establish MaskGraphene as a versatile foundation for next-generation spatial omics integration, empowering deeper insights into tissue architecture and cellular dynamics in both health and disease.

## Methods

### MaskGraphene workflow

The MaskGraphene workflow (Fig. 1) takes as input a spot-gene expression matrix and spatial coordinates from two or more tissue slices. It begins with an initial integration using a masked graph attention autoencoder backbone with an intra-slice k-NN graph, jointly optimized with a masked self-supervised loss [36–38] and a triplet loss [27, 63] to identify shared clusters across slices. Triplet loss serves as “soft-links” in the latent space to strengthen inter-slice connections for initial integration. Next, cluster-wise local alignment is performed to establish “hard-links” (mappings) between slices, enabling all spots to be represented within a unified coordinate system. Using these unified coordinates, an inter-slice k-NN graph is constructed to model spatial relationships across slices. Finally, leveraging the same masked graph attention autoencoder architecture with the updated k-NN graph, MaskGraphene conducts a final integration step to optimize low-dimensional joint embeddings. Further details are provided in the corresponding sections below. With the final joint embeddings, MaskGraphene seamlessly integrates ST slices, enabling the identification of similar spatial domains or cell types across different tissue slices. We demonstrate MaskGraphene’s effectiveness through various quantitative and qualitative downstream analyses.

### Initial integration (Steps 1-2)

As illustrated in Steps 1-2 of Fig. 1, MaskGraphene generates initial joint embeddings through an initial integration across spatial slices. Specifically, it employs a masked graph attention autoencoder to learn low-dimensional latent embeddings by jointly minimizing a masked self-supervised reconstruction loss and a triplet loss. Details of the graph model architecture, loss design, training strategy, and outputs of the initial integration are described in the subsections below.

### Graph input processing

MaskGraphene processes gene expression data and spatial coordinates from two or more ST slices. First, it normalizes the total count of raw gene expressions and log-transforms them using the Scanpy package [44]. It then identifies the highly variable genes (HVGs) from all slices, and focuses on their HVGs intersection to maintain the consistency of expression features. For large-panel datasets (e.g., DLPFC, Stereo-seq), we select the top 5,000 HVGs to retain informative features while reducing noise. For small-panel datasets (e.g., MERFISH), we retain all genes to avoid discarding potentially critical information in low-dimensional settings.

### Graph model backbone

In this section, we present the mathematical formulation and details of our backbone, the graph attention autoencoder. This model comprises five main components: the encoder, graph attention mechanism, decoder, projector, and generator.

**Encoder.** The encoder generates node (spot) embeddings by aggregating information from all neighbors. We denote $\textbf{h}_u^{(0)}$ as the feature of spot *u*. In the masked graph modeling framework used in MaskGraphene, a subset of these node features is intentionally masked to enable self-supervised reconstruction, encouraging the encoder to capture context-aware representations. The specific masking strategy is described in the following section. The $l^{\text {th}}$ encoder layer generates the embedding of spot *u* in layer *l* as follows:

1 $$\begin{aligned} \textbf{h}^{(l)}_u = \epsilon \left( \sum \limits _{v \in \mathcal {N}_u} \alpha ^{(l)}_{uv} (\textbf{W}^{(l)} \textbf{h}_v^{(l-1)})\right) \end{aligned}$$

where $\alpha ^{(l)}_{uv}$ is the attention coefficient, discussed further below, $\epsilon$ is the activation function, $\mathcal {N}_u$ denotes all the neighbors of node *u* including *u* itself, $\textbf{h}_{v}^{(l-1)}$ denotes the embedding of node *v* in the $l-1$ layer, and $\textbf{W}^{(l)}$ is the matrix of trainable parameters in $l^\text {th}$ layer.

**Graph attention mechanism.** The attention coefficient $\alpha _{uv}^{(l)}$ is calculated by (2) and (3) where $\oplus$ is the concatenation operation and $\sigma$ is a sigmoid activation function.

2 $$\begin{aligned} \alpha ^{(l)}_{uv}=\frac{\exp {(\sigma (\textbf{a}^{{(l)}^{T}}{\textbf{g}}_{uv}^{(l)}))})}{\sum \nolimits _{v^{'} \in \mathcal {N}_{u}}\exp {(\sigma (\textbf{a}^{{(l)}^{T}}{\textbf{g}}_{uv^{'}}^{(l)}))}} \end{aligned}$$

3 $$\begin{aligned} {\textbf{g}}^{l}_{uv} = \textbf{W}^{(l)}\textbf{h}_{u}^{(l-1)} \oplus \textbf{W}^{(l)}\textbf{h}_{v}^{(l-1)} \end{aligned}$$

where $\textbf{a}^{(l)}$ is another learnable parameter. The attention mechanism here is exploited to strengthen the connection between nodes that are represented by similar expression profiles. The attention coefficient $\alpha ^{(l)}_{uv}$ indicates the different contributions of each neighbor used in the aggregation process. The weights of edges are automatically calculated, based on node embedding.

**Decoder.** The decoder attempts to reconstruct the normalized expression profile for each spot *u* given the latent embeddings of the encoder. The $l^{\text {th}}$ layer of the decoder (from the perspective of spot *u*) defined as:

4 $$\begin{aligned} \hat{\textbf{h}}^{(l)}_u = \epsilon \left( \sum \limits _{v \in \mathcal {N}_u} \hat{\alpha }^{(l)}_{uv} (\hat{\textbf{W}}^{(l)} \hat{\textbf{h}}_v^{(l-1)})\right) \end{aligned}$$

where $\hat{\alpha }^{(l)}_{uv}$ is the decoder attention coefficient which is calculated similarly as $\alpha ^{(k)}_{uv}$ in the encoder, and $\hat{\textbf{W}}^{(l)}$ is the matrix of trainable parameters in $l^\text {th}$ layer.

The reconstruction loss function is introduced in the next Methods section in the context of masked self-supervised loss.

**Projector.** The projector is a multi-layer perceptron (MLP) serving as the decoder for input feature reconstruction. It consists of two layers that map the latent space to the representation space for prediction.

5 $$\begin{aligned} \bar{\textbf{Z}} = {MLP}(\textbf{H}) \end{aligned}$$

where $\textbf{H}$ denotes the latent embedding of encoder from either a masked or an unmasked graph in MaskGraphene, as illustrated in Fig. 1, and $\bar{\textbf{Z}}$ denotes the output of projector.

**Generator.** The generator has an identical structure to the aforementioned encoder and projector but with a different set of trainable parameters $\xi _{enc}$ and $\xi _{proj}$. It works on the unmasked graph input to produce the latent target representation $\bar{\textbf{X}}$, which is leveraged to train the encoder and projector network to match the output of the generator on masked nodes.

6 $$\begin{aligned} \bar{\textbf{X}} = Projector(Encoder(\textbf{A},\textbf{X}; \xi _{enc}); \xi _{proj}) \end{aligned}$$

where $\textbf{A}$ and $\textbf{X}$ denote the adjacency matrix and input node features of the unmasked graph.

We currently employ a symmetric encoder structure of 512, 32 (and 32, 512 for the decoder) in the MaskGraphene model. Detailed grid search results for the sensitivity analysis are provided in Additional file 1: Note S2 and Fig. S33.

### Masked self-supervised reconstruction loss

For our graph model backbone, we introduced a self-supervised loss to reconstruct the node features that are randomly masked from the input gene expression matrix.

Specifically, MaskGraphene perturbs the input graph by masking node features and then attempts to reconstruct the original input. A subset of input spots is randomly selected, and their expression profiles are set to zero. A re-mask decoding strategy is also adopted, in which the latent embeddings of masked nodes are stochastically re-masked prior to decoding. The masking and re-masking rates are empirically set to 0.1/0.1 or 0.5/0.5 for the two dataset types (see Additional file 1: Note S2 and Fig. S34 for details and sensitivity analysis). This strategy aims to enforce the reconstruction in the embedding space and regularize the node feature reconstruction.

For each node $i$, let $\textbf{z}_i$ represent its reconstructed gene expression via the decoder. The reconstruction loss function is formulated as:

7 $$\begin{aligned} \mathcal {L}_{\text {masked}} = \frac{1}{|\bar{V}|}\sum \limits _{v_i \in \bar{V}} \left( 1 - \frac{\textbf{x}_{\textbf{i}}^{T}\textbf{z}_{\textbf{i}}}{||\textbf{x}_{\textbf{i}}|| \cdot ||\textbf{z}_{\textbf{i}}||} \right) ^\mathbf {\gamma }, \mathbf {\gamma } \ge 1, \end{aligned}$$

where $\textbf{x}_i$ denotes the original normalized expression profile for spot *i*, $\bar{V}$ is the subset of spots with masked features in the graph, and $\gamma$ is a scaling factor for adjusting loss (empirically set to 1; see Additional file 1: Note S2 and Fig. S35 for sensitivity analysis). Using this scaled cosine error loss to measure the reconstruction error, the model learns node embeddings within a masked autoencoder framework.

To further leverage the feature information, a supporting network is utilized as the target generator to produce latent prediction targets from the unmasked graph. This generator, as previously described, shares the same structure as the aforementioned encoder and projector but operates with a different set of trainable parameters. MaskGraphene simultaneously optimizes the parameters of the encoder, projector, and generator by minimizing the following loss function:

8 $$\begin{aligned} \mathcal {L}_{\text {latent}} = \frac{1}{N}\sum \limits _{i}^{N} \left( 1 - \frac{\bar{\textbf{z}}_{\textbf{i}}^{T}\bar{\textbf{x}}_{\textbf{i}}}{||\bar{\textbf{x}}_{\textbf{i}}|| \cdot ||\bar{\textbf{z}}_{\textbf{i}}||} \right) ^\mathbf {\gamma }, \mathbf {\gamma } \ge 1, \end{aligned}$$

where *N* denotes all the spots in the unmasked graph, $\bar{\textbf{z}}_{i}$ represents the output of projector from the masked graph, and $\bar{\textbf{x}}_{i}$ denotes the output latent targets of generator from the unmasked graph.

MaskGraphene simultaneously optimizes two objective functions with a balancing coefficient $\lambda$ (empirically set to 1; see Additional file 1: Note S2 and Fig. S35 for sensitivity analysis):

9 $$\begin{aligned} \mathcal {L}_{\text {masked self-supervised}} = \mathcal {L}_{\text {masked}} + \lambda \mathcal {L}_{\text {latent}} \end{aligned}$$

The intuition behind this loss design is to prevent the network from oversmoothing singular yet biologically critical features in the gene expression profiles, while simultaneously suppressing noise from batch effects and other technical artifacts. Such masking could lead to ambiguity between real and artificially masked zeros. However, our framework incorporates an auxiliary network (the generator and projector components described in the network structure) that processes the unmasked expression data and its corresponding embeddings. This auxiliary branch contributes to the joint optimization through the latent loss and helps regularize the learning process, providing the model with access to the true data distribution. As a result, even though the main network operates on masked input (with zeros as placeholders), the auxiliary network helps guide the learning dynamics and mitigates potential confusion arising from the sparsity. This dual-network design offers a practical balance between effective masking and accurate optimization in sparse settings.

### Triplet loss

To learn the initial joint embeddings in the graph model, MaskGraphene leverages “soft-links” to enforce local geometric structure preservation among spots across slices and enhance inter-slice connections.

For “soft-links”, MaskGraphene employs mutual nearest neighbors (MNNs) [64] from each pair of consecutive slices to dynamically construct spot triplets based on their embeddings during training. The inter-slice connections, denoted as “soft-links”, differ from “hard-links” - introduced in Step 3 of the alignment and incorporated in Steps 4-5 of the final integration - in that they are not directly integrated into the k-NN graph. Instead, they are reinforced through the triplet loss, a method often used in single-cell RNA-seq batch correction [27, 63]. These “soft-links” are designed to emphasize differences between dissimilar spots across slices while clustering similar spots closer together. The objective is to bring similar spots nearer and separate contrasting ones [63].

In detail, triplet loss is based on the idea of triplets, which consist of an anchor spot $\textbf{a}$, a positive spot $\textbf{p}$, and a negative spot $\textbf{n}$. For each triplet $(\textbf{a}, \textbf{p}, \textbf{n})$, the loss is defined as follows:

10 $$\begin{aligned} \mathcal {L}_{\text {triplet}} = \max \left( {Distance}(\textbf{a}, \textbf{p}) - {Distance}(\textbf{a}, \textbf{n}) + \alpha , 0\right) \end{aligned}$$

where ${Distance}(\textbf{a}, \textbf{p})$ is the Euclidean distance between the anchor spot $\textbf{a}$ and the positive spot $\textbf{p}$, measured in the joint embedding space, ${Distance}(\textbf{a}, \textbf{n})$ is the distance between the anchor spot $\textbf{a}$ and the negative spot $\textbf{n}$, $\alpha$ is a margin that controls the minimum difference required between the distances of similar and dissimilar pairs. To construct triplets, we first determine MNNs across two slices. MNNs are constructed by taking the pairs of spots from distinct slices that are mutually k-nearest-neighbors [65] in the joint embedding space. After we identify MNNs matches between two slices, we define each MNN match as the $(\textbf{a}, \textbf{p})$ pair. We further take each spot from MNN matches of one slice as an anchor spot and randomly select any spot from the other slice as a negative spot to form the $(\textbf{a}, \textbf{n})$ pair. An alternating optimization strategy is adopted to optimize triplet loss. Specifically, every 100 training epochs (during which the model is primarily minimizing the masked self-supervised reconstruction loss), we recompute the triplets using the most recent low-dimensional embeddings. This updated triplet set better reflects the current embedding space and allows the triplet loss to be further minimized. This iterative process ensures that the triplet loss remains relevant throughout training and helps improve the alignment and integration of spatial slices. Triplet loss encourages the model to pull the anchor and positive spots (MNNs across slices) closer in the embedding space while pushing the anchor and negative spots (dissimilar spots across slices) farther apart.

### Training strategy

During training, MaskGraphene adopts a two-phase optimization strategy to ensure stable convergence and robust embedding learning. In the first phase, all components of the model, including the encoder, projector, generator, and decoder, are trained jointly using backpropagation and a shared optimizer (e.g., Adam). This joint training enables the different modules to co-adapt effectively. The model is optimized using a combination of masked reconstruction loss and latent loss. The masked loss encourages accurate recovery of missing gene expression values under partial feature masking, using a scaled cosine error to handle variations in feature magnitude. At the same time, the latent loss enforces consistency between the encoder and projector outputs by minimizing the difference between latent representations from raw and masked inputs. This helps stabilize the embedding space and prevents collapse. After the model learns a stable embedding, we enter the second phase and introduce a triplet loss over soft-links. This loss pulls similar spots closer and pushes dissimilar ones apart, refining the embedding space to better preserve spatial structure and improve cross-slice alignment. Delaying the triplet loss until after initial convergence avoids early-stage instability and enables more effective metric learning.

### Outputs of initial integration

After training the masked graph attention network with soft-links, we obtain joint spot embeddings across all slices. These embeddings are then used to perform joint clustering using mclust [48], yielding the initial clustering output. These initial embeddings, though sufficient for defining shared clusters across slices, are not yet fully optimized for spatial accuracy. They serve as a starting point for the next step - cluster-wise local alignment - which produces hard-links that refine inter-slice relationships and lead to a second round of embedding optimization.

### Cluster-wise local alignment to generate “hard-links” (Step 3)

After identifying shared clusters across slices - each representing a common tissue domain - as illustrated in Step 3 of Fig. 1, we perform cluster-wise local alignment to establish direct correspondences (hard-links) between spots across slices. Rather than conducting a global alignment across all spots, which can be computationally intensive for large-scale datasets, we restrict alignment to spots within matched clusters. This localized strategy enhances computational efficiency and reduces false positive mappings, particularly in cases where slices are only partially overlapping or not perfectly adjacent along the z-axis. Details of the local alignment method are described in the subsections below.

### Spot-to-spot mapping

To enhance inter-slice connections, we developed a cluster-wise optimal transport (OT)-based local alignment method to establish reliable spot-to-spot (node-to-node) mapping (alignment) across slices, enabling the unification of all spots into a shared coordinate system for constructing the inter-slice k-NN graph in MaskGraphene’s graph model during Steps 4-5 of the final integration. This cluster-wise alignment process begins with the previous initial integration step, which performs joint clustering to identify shared clusters across slices. Once shared clusters are identified across slices, cluster-wise OT-based local alignment is performed. Specifically, for each pair of shared clusters (representing the same domain across each pair of slices), following prior work on spatial slice alignment (e.g., PASTE [31]), we adopt a standard Gromov-Wasserstein optimal transport (GW-OT) formulation to compute probabilistic couplings between spots and derive spot-to-spot mappings. Unlike PASTE, which solves a single global fused GW problem per adjacent slice pair, MaskGraphene solves multiple cluster-wise GW problems over matched domains across all slices. Aggregating these local alignments provides global context while improving robustness to partial overlap and localized deformations. Each cluster-wise GW problem is optimized with an iterative conditional gradient method [66].

A detailed description of the problem is provided in Additional file 1: Methods S1. Given *H* slices that have $M$ shared clusters identified from the initial clustering, the complete mapping (alignment) $\Pi ^h$ between the two slices *h* and $h+1$ is obtained by combining the transport plans (weights) across all clusters, with spots ordered according to their cluster labels:

11 $$\begin{aligned} \Pi ^h = \left[ \begin{array}{ccccccc} \Pi ^{h_1} & 0 & 0 & \cdots & 0 & \cdots & 0 \\ 0 & \Pi ^{h_2} & 0 & \cdots & 0 & \cdots & 0 \\ 0 & 0 & \cdots & \Pi ^{h_m} & \cdots & 0 & 0 \\ \vdots & \vdots & \cdots & \vdots & \cdots & \vdots & \vdots \\ 0 & 0 & 0 & \cdots & 0 & \cdots & \Pi ^{h_M} \end{array}\right] , \end{aligned}$$

where $\Pi ^{h_m} = [\pi ^{h_m}_{ij}]$ is the transport plan (weight) between slices $h$ and $h+1$ in cluster $m$. By deriving shared clusters from an initial integration of all slices and assembling $\Pi$ from multiple small, cluster-wise GW problems (across all adjacent slice pairs), our local mapping is globally informed by the entire set of slices.

### Coordinate system unification

After obtaining the transport weights (spot-to-spot mapping) for all clusters, referred to as “hard-links”, MaskGraphene integrates these inter-slice connections into its final k-NN graph model by employing three distinct strategies, each designed for specific integration scenarios.

Firstly, when the two slices $(X, D, g)$ and $(X', D', g')$ are adjacent consecutive slices and are used for pairwise integration (e.g., pairwise DLPFC, MHypo, and breast cancer slices sampled along the z-axis; or mouse and Drosophila embryo slices at closely matched developmental stages, as in this study.), we project the second slice to the same coordinate system as the first slice after we find the mappings $\Pi$. The following generalized weighted Procrustes problem is solved to apply for coordinate transformation [31]:

12 $$\begin{aligned} \hat{R}, \hat{v} = \min _{R \in \mathbb {R}^{2 \times 2}, v \in \mathbb {R}^2, R^T R = I} \sum \limits _{i,j} \pi _{ij} \Vert z_{.i} - Rz_{.j}' - v \Vert ^2, \end{aligned}$$

where $\hat{R}$ is the rotation matrix and $\hat{v}$ is the translation vector.

The updated spatial coordinates of spot $j$ in slice $(X', D', g')$ are then given by:

13 $$\begin{aligned} \hat{z}_{.j}' = \hat{R} z_{.j}' + \hat{v}. \end{aligned}$$

Secondly, when multiple (>2) consecutive ST slices (e.g., DLPFC four-slice, MHypo five-slice, MB ten-slice, and mouse embryo six-slice integration in this study) are available for integration, achieving a robust unified coordinate system across all slices becomes increasingly challenging, particularly as the number of slices grows and geometric similarity between slices diminishes, with some adjacent slices exhibiting only partial overlap. MaskGraphene addresses this challenge by employing two alternative strategies: (1) coordinate replacement and (2) coordinate transformation, both designed to unify the coordinate system across slices. As shown in our results for the DLPFC, MHypo, and MB multi-slice integration, both strategies perform comparably well. Users may choose either strategy; however, for integration involving a large number of slices (e.g., >10), we recommend using coordinate transformation for greater robustness.

(1) Coordinate replacement via hard-links (by default): in this approach, we first compute spot-to-spot correspondences for all adjacent pairs of slices (1-2, 2-3, ...) using our cluster-wise local alignment, which outputs transport plans (weights) $\Pi$ reflecting both spatial proximity and transcriptional similarity. For each spot in slice $i+1$, we define its highest-confidence counterpart in slice *i* as the match with the largest normalized OT transport weight. After obtaining these highest-confidence mappings for all adjacent pairs, we perform a backward sweep from the last slice to the first. At each step, we replace every spot’s coordinates in slice *i* with those of its highest-confidence counterpart in slice $i-1$. Because earlier slices have already been updated in prior steps, these replacements are recursively composed, ensuring that, by the end of the sweep, all slices are expressed in the coordinate system of slice 1. This cascading update strategy preserves local geometric accuracy while reducing error accumulation, enabling efficient construction of a multi-slice k-NN graph that integrates both intraslice and inter-slice connections without requiring any additional global transformations.

(2) Coordinate transformation via optimization: building on the local shared clusters (domains) identified during initial integration, we perform spatial coordinate transformation across multiple slices by predicting their coordinate systems using a mutual nearest neighbor (MNN) graph constructed from the unified spatial coordinates of the spots. The process is framed as an optimization problem guided by an objective function [33]. To address partially overlapping slices during multi-slice integration, a penalty term is incorporated to account for the proportion of overlapping spots. Using the differential evolution optimization algorithm [67], we determine the optimal transformation parameters - including translation vectors and rotation angles - for each slice. These parameters are then used to transform the coordinates of each slice. A detailed description of the optimization problem is provided in Additional file 1: Methods S2.

In summary, for multi-slice integration, users can select either coordinate replacement or coordinate transformation to unify the coordinate system across slices. In our study, both methods exhibited comparable performance overall; however, coordinate transformation demonstrated superior results for MB ten-slice integration.

Thirdly, when horizontally consecutive slices are integrated (e.g. mouse brain sagittal sections divided into anterior and posterior portions in this study), “hard-links” are directly incorporated as edges in the k-NN graph to strengthen inter-slice connections. Coordinate transformation or replacement is unnecessary, as these slices are horizontally connected. Subsequently, the final k-NN graph, incorporating both intraslice and inter-slice connections, is constructed.

It is worth noting that MaskGraphenes allows users to also utilize other state-of-the-art alignment tools to generate transport weights (spot-to-spot mappings) and unify coordinates across slices.

### Final embedding optimization (Steps 4-5)

After performing cluster-wise local alignment, we generate hard-links between slices and unify their coordinate systems. These alignment-derived connections are considered hard-links because they are explicitly added as inter-slice edges in the k-NN graph. We then retrain the masked graph attention network using both the hard-links (from alignment) and soft-links (from the triplet loss) to carry out the final integration. This process yields the final joint embeddings across slices, as illustrated in Fig. 1. By incorporating alignment-based hard-links, we strengthen inter-slice connectivity and enable the model to better preserve the underlying geometric structure across slices, thereby enhancing the overall integration quality. These final joint embeddings can then be leveraged for a variety of downstream analyses.

In constructing the k-NN graph, we adopt an empirical strategy to determine the number of neighbors *k*. Specifically, we fix the within-slice *k* to 6 for all datasets, and set $k=15$ when performing coordinate unification across slices. To assess sensitivity, we conducted a grid search over a range of *k* values and observed that the model performance remained largely consistent, demonstrating its robustness to this hyperparameter choice.

The primary distinction between the initial and final joint embeddings lies in the incorporation of hard links into the k-NN graph during the latter stage. The initial embeddings are generated to facilitate shared cluster/domain identification across slices, serving as a foundation for cluster-wise local alignment. Thus, the main objective of the initial integration is to enhance domain detection through joint embedding. In contrast, the final integration step further refines the joint embeddings by explicitly incorporating spatial hard links, aiming to preserve the original geometric structure for improved interpretability.

As detailed in Additional file 1: Note S1 and Figs. S36-S37, we compared the initial and final embeddings, and further benchmarked the initial embeddings against those from STAligner. In MaskGraphene, the initial embeddings are generated using slice-specific k-NN graphs combined with a triplet loss, aligning with the foundational strategy adopted by ST integration methods such as STAligner. This evaluation underscores the added benefit of the masking mechanism in our graph model for cross-slice domain identification. Our results showed that the initial embeddings outperformed STAligner in clustering performance, as measured by ARI, across both pairwise and multi-slice integration tasks. This performance was further enhanced by the final embeddings. Overall, the final embeddings demonstrated the highest quality in terms of iLISI, Isometry correlation, and Procrustes dissimilarity, followed by the initial embeddings and then STAligner.

### Ablation study design and model variants

To assess the contribution of each component in MaskGraphene, we performed a comprehensive ablation study by systematically modifying the model’s architecture and loss functions. The full model, referred to as MaskGraphene-Full, integrates four key modules: a) a graph encoder utilizing hard inter-slice links to capture geometric priors, b) a masked reconstruction loss to support self-supervised feature learning, c) a triplet loss to enforce soft similarity constraints across slices, and d) a latent regularization loss to improve stability and generalization. This configuration served as the reference for all comparisons.

To isolate the role of each component, we constructed the following model variants (summarized in Additional file 1: Table S3): (1) MaskGraphene-MLP: A baseline that removes all graph-based operations and the masking strategy. It employs a simple multilayer perceptron (MLP) to process the input features and retains only the reconstruction and triplet losses. This variant evaluates the performance of a purely feature-based model without spatial priors or self-supervised regularization. (2) MaskGraphene-GAE: A graph autoencoder variant that retains the hard-link graph structure and the triplet loss but excludes the masked reconstruction mechanism. Since the latent loss is designed to work in conjunction with masked features, it is also removed. This variant isolates the contribution of geometric encoding without the self-supervised masking framework. (3) MaskGraphene-HardlinkOnly (NoTriplet): This variant removes the triplet loss, thereby eliminating soft similarity-based alignment across slices. It retains hard-links, masked reconstruction loss, and latent loss. This setup evaluates whether rigid structural priors alone are sufficient for achieving meaningful alignment. (4) MaskGraphene-MaskOnly (NoAuxLoss): This model retains the masked reconstruction and the graph encoder but omits both the triplet loss and latent loss. It serves to examine whether masked modeling alone, in the absence of auxiliary guidance, can support robust integration. For model variants (1) - (4), the initial integration and alignment steps were kept identical to those of the full model to ensure that the hard-links remained unchanged, even not used at all. (5) MaskGraphene-SoftlinkOnly: This configuration eliminates hard inter-slice links in the final embedding optimization steps, relying solely on soft similarity constraints introduced by the triplet loss. It retains the masked reconstruction and latent loss components. This variant tests whether soft-links, without explicit spatial priors, can guide effective integration across slices.

All models were trained using the same pipeline and hyperparameters to ensure fair comparison. We evaluated each variant using a comprehensive suite of quantitative and qualitative metrics, including layer-wise alignment accuracy, spot-to-spot matching ratio, iLISI, Isometry correlation, Procrustes dissimilarity, and UMAP visualizations. This ablation framework enables a fine-grained analysis of the role played by masking strategies, graph structure, and auxiliary objectives in achieving accurate, interpretable, and spatially coherent integration of multi-slice ST data.

### Random seed analysis

The graph model is non-deterministic and may exhibit variance, especially in clustering outcomes. To generate Adjusted Rand Index (ARI) values for clustering performance, we fixed the random seeds in MaskGraphene for all datasets. For tools that do not fix seeds, such as STAligner and DeepST, we selected the best ARI value from 10 runs for each experiment. Additionally, we evaluated the robustness of all tools by varying the random seed and performing 20 iterations for each integration experiment, with ARI box plots illustrating the results. As shown in Additional file 1: Fig. S38, MaskGraphene consistently outperformed other tools across nearly all integration scenarios, highlighting its robustness.

### Benchmark datasets

We employed seven ST datasets with a total of 53 slices for benchmarking, which had corresponding manual annotations shown in Additional file 1: Table S1.

Specifically, (1) the human DLPFC (dorsolateral prefrontal cortex) dataset, generated with 10x Visium, includes 12 human DLPFC sections with manual annotation, indicating cortical layers 1 to 6 and white matter (WM), taken from three individual samples [39]. Each sample contains four consecutive slices (for example, slice A, B, C, and D in order). In each sample, the initial pair of slices, AB, and the final pair, CD, are directly adjacent (10$\mu$m apart), whereas the intermediate pair, BC, is situated 300$\mu$m apart. The number of spots ranges from 3,431 to 4,788, with a total of 33,538 expressed genes across all slices.

(2) The MHypo (mouse hypothalamus) dataset by MERFISH contains five manually annotated consecutive slices [23] labeled Bregma -0.04mm (5,488 cells), Bregma -0.09mm (5,557 cells), Bregma -0.14mm (5,926 cells), Bregma -0.19mm (5,803 cells), and Bregma -0.24mm (5,543 cells). Expression measurements were taken for a common set of 155 genes. Each tissue slice includes a detailed cell annotation, identifying eight structures: third ventricle (V3), bed nuclei of the stria terminalis (BST), columns of the fornix (fx), medial preoptic area (MPA), medial preoptic nucleus (MPN), periventricular hypothalamic nucleus (PV), paraventricular hypothalamic nucleus (PVH), and paraventricular nucleus of the thalamus (PVT).

(3) The MB (mouse brain) dataset [33, 49] by MERFISH has a total of 33 consecutive mouse primary motor cortex tissue slices with similar shapes, which can be used for 3D reconstruction. We selected the first 10 consecutive slices for benchmarking in this study. Region annotations include the six layers (L1-L6) and white matter (WM). The number of spots ranges from 2,033 to 5,624, with a total of 254 expressed genes.

(4) The dataset of mouse brain sagittal sections (MB2SA&P), generated with 10x Visium, includes two slices of the anterior and posterior mouse brain. The number of spots is 2,695 and 3,353 for two slices, respectively, with both containing 32,285 expressed genes. Only the anterior section includes annotations [13, 50].

(5) The mouse embryo dataset by Stereo-seq has over 50 slices, and the slices at time points E11.5 to E16.5 were used in our experiments. The number of spots is 30,124 and 51,365 for slices E11.5 and E12.5, respectively, with 26,854 and 27,810 captured and expressed genes. In total, these six slices contain 496,494 spots. These data are from a large stereo-seq project called MOSTA [15]: Mouse Organogenesis Spatiotemporal Transcriptomic Atlas by BGI.

(6) The 10x Visium dataset for human breast cancer from the 10x Genomics database contains two slices with approximately 3,800 spots and 36,000 genes. Manual annotations are available for one slice, defining 20 regions, including DCIS/LCIS, healthy tissue, invasive ductal carcinoma (IDC), and low-grade tumor margins [68]. We applied our local alignment module to align spots across the two slices and transferred domain labels from the annotated slice to the second slice.

(7) The Drosophila embryo dataset by Stereo-seq comprises 16 slices [47]. In the original study, Stereo-seq was applied to spatially profile gene expression in Drosophila melanogaster across multiple developmental stages, including two late-stage embryos (14-16 hours and 16-18 hours after egg laying, denoted E14-16 and E16-18) and three larval stages (L1, L2, and L3). Each spatial slice contains approximately 1,000 spots, with a median of $\sim 2$,000 unique molecular identifiers (UMIs) per spot. Cell type annotations were obtained through unsupervised clustering of gene expression profiles, followed by manual labeling using known marker genes.

### Simulated datasets

Due to the limited availability of benchmark datasets for evaluating spot-to-spot alignment accuracy, we employed our recent ST simulation method [16] to generate five simulated 10x Visium datasets for this purpose. Specifically, we used the DLPFC 151673 slice as the reference and created new slices by altering the spatial coordinates of the reference through rotation. To further simulate real-world scenarios, we adjusted the number of spots in each slice by removing spots that no longer aligned with the grid coordinates after rotation. To preserve the fidelity of real 10x Visium data, we arranged the tissue spots in a hexagonal grid pattern rather than a rectangular one. By applying rotations with different degrees, we generated slices with varying overlap ratios (20%, 40%, 60%, 80%, and 100%) relative to the reference slice.

### Quantitative metrics

#### Adjusted Rand Index (ARI).

ARI measures the similarity between two data clusterings by assessing the concordance of pairwise data point groupings. It evaluates whether pairs of points are assigned to the same cluster or different clusters in the two clusterings. The ARI ranges from -1 to 1, where 1 represents perfect agreement, 0 indicates clustering that is no better than random, and negative values suggest disagreement between the clusterings.

#### Layer-wise alignment accuracy.

This metric [16] is based on the hypothesis that aligned spots from adjacent consecutive slices are likely to belong to the same spatial domain or cell type. Joint spot embeddings from each method are used to align (anchor) spots from the first slice to corresponding (aligned) spots on the second slice for each slice pair. Spot proximity for alignment is determined using Euclidean distance. Alignment accuracy is calculated as the proportion of anchor spots in the first slice that are aligned to spots in the second slice belonging to the same spatial domain or cell type. For the DLPFC slices, which exhibit a unique layered structure, this metric is further designed to assess whether anchor and aligned spots belong to the same layer (layer shift = 0) or they belong to different layers (layer shift = 1 to 6). This design ensures the metric accurately captures alignment performance within the unique context of layered structures.

#### Spot-to-spot matching ratio.

This metric [16] quantifies the accuracy of alignment between corresponding spots on adjacent tissue slices. It is defined as the ratio of anchor spots in the first slice to aligned spots in the second. An optimal integration method for two adjacent consecutive slices would ideally result in a 1:1 ratio, indicating perfect alignment fidelity.

#### Spot-to-spot alignment accuracy.

To assess spot alignment across slices, this metric [16] is applied to simulated datasets with known ground truth, measuring spot-wise alignment accuracy. It is defined as the percentage of anchor spots in the simulated slice that are correctly matched to their corresponding aligned spots in the reference slice.

#### Pearson correlation coefficient.

To assess the alignment of spots, we computed the Pearson correlation coefficient for matched spots identified using known ground truth in simulated datasets. This coefficient measures the strength and direction of the linear relationship between the embeddings for the matched spots, with values closer to 1 indicating a stronger linear alignment.

#### Integration Local Inverse Simpson’s Index (iLISI).

The iLISI metric [21] is used to assess the quality of batch mixing following data integration in spatial transcriptomics or single-cell RNA sequencing. It quantifies the extent to which the integrated data achieves a balance between preserving biological variability across cell types and promoting effective batch mixing. iLISI ranges from 0 to 1. A higher iLISI score indicates more even mixing of spots or cells from different batches, reflecting the effectiveness of the integration method in mitigating batch effects while preserving the underlying biological structure of the data.

#### Isometry correlation and Procrustes dissimilarity.

In this study, geometric information preservation refers to maintaining the spatial structure and relationships between spots or cells when projecting into latent embedding space after ST integration. This involves preserving both isometric relationships and the overall similarity in spatial arrangements across datasets. To assess the quality of preservation, we used two metrics, Isometry correlation and Procrustes dissimilarity.

Isometry correlation evaluates whether the relative distances between points in the original spatial data are preserved in the integrated or embedded space. Ideally, points that are close in the original space should remain close after integration, while points that are far apart should maintain proportional distances. Isometry correlation ranges from 0 to 1. A higher Isometry correlation indicates that the intrinsic spatial organization of the data is well-preserved, supporting accurate biological interpretation. Procrustes dissimilarity, on the other hand, compares two datasets by applying transformations such as scaling, rotation, and reflection to align one dataset as closely as possible with the other. This metric quantifies how well the original tissue geometry is preserved in the embedding space. Procrustes dissimilarity ranges from 0 to 1. A smaller Procrustes dissimilarity value indicates better alignment, reflecting that the spatial relationships between spots or cells have been well-preserved across datasets.

### Qualitative analysis

#### Visualization of aligned, misaligned, and unaligned spots from pairwise integration and alignment.

To visually evaluate the quality of joint spot embeddings, we aligned each spot on the first slice (the “anchor” spot) with a corresponding spot on the second slice (the “aligned” spot) based on their joint latent embeddings generated by each integration method, using Euclidean distance. If the aligned spot belonged to the same spatial domain or cell type as the anchor spot based on ground truth labels, both spots were classified as “aligned” and marked in orange (Fig. 3a-b). If the aligned spot differed in spatial domain or cell type from the anchor spot, both were classified as “mis-aligned” and marked in blue. Finally, spots on the second slice that were not aligned to any spot on the first slice were classified as “unaligned” and marked in green (Fig. 3a-b).

#### Spatial visualization of joint domain identification through clustering results after integration.

For all datasets we used, we compared the identified domains across slices after integration with the ground truth labels. For the dataset of mouse brain sagittal sections, we further compared the identified domains after integration with the Allen Brain atlas through visualization. Additionally, we assessed the consistency of regions across the fissure between the anterior and posterior sections. Greater similarity to the atlas and improved regional coherence indicate superior integration performance.

#### Visualization of UMAP plot for joint embeddings.

The majority of integration techniques focus on embedding spots into a low-dimensional latent space, which can often be difficult to interpret intuitively. To improve understanding of the distribution within the latent space, we applied UMAP for dimensionality reduction, projecting the spot embeddings into two dimensions. An effective UMAP visualization of latent embeddings should display patterns that reflect the characteristics of the original data while clearly delineating spatial domains or cell types.

#### Partition-based graph abstraction (PAGA) analysis.

PAGA is a computational framework primarily used in single-cell genomics to infer and visualize the connectivity structure of cell clusters or partitions, often along developmental trajectories [69]. It combines graph-based clustering with a simplified abstract representation of the relationships between cell groups, providing insights into the hierarchical or sequential organization of data. In this study, we used joint embeddings generated by the integration method as input for Scanpy to construct the PAGA graph, uncovering the spatial trajectory of cortical layers in both the DLPFC and MB datasets.

#### Biomarker identification analysis.

To evaluate whether integration enhances biomarker identification, we compared domain-specific marker genes identified from individual slices based on ground truth labels with biomarkers derived from each integrated domain across four slices. Initially, predicted domains from the integration tool were aligned with ground truth labels to annotate the domains, and spots corresponding to each domain were extracted from all integrated slices. Biomarkers for each integrated domain were then identified using Scanpy. To create ground truth domain marker genes for comparison, we applied Scanpy to identify biomarkers for each domain in individual slices based on ground truth labels, selecting the top 50 or 100 genes per domain per slice. The union of these top genes, denoted as $N$, across all slices was compiled to form the final set of ground truth domain marker genes for validation. Finally, we computed the overlap ratio between the top $N$ biomarkers identified for each integrated domain and the $N$ ground truth domain marker genes. We performed this analysis for both DLPFC and mouse embryo datasets.

#### Isodepth gradient analysis.

To validate the quality of joint embedding, we employed GASTON [3], an unsupervised and interpretable deep learning algorithm designed to generate a topographic map of brain tissue slices, and reveal gradients of neuronal differentiation and activity. GASTON constructs this topographic map by leveraging an innovative 1-dimensional coordinate system, termed isodepth, to define spatial organization within the tissue. Analogous to contour lines on a geographic map, isodepth contours delineate distinct spatial domains and quantify gradual expression changes across these regions.

We compared the joint embedding of MaskGraphene with STAligner and GraphST under two different scenarios when running GASTON. By default, GASTON uses the two-dimensional X,Y coordinates of all spots and the principal components (PCs) of the expression data from a single slice to fit the above models. In the first experimental scenario, we replaced the PCs with the joint embedding from each integration method while retaining the original X,Y coordinates as input to GASTON. In the second experimental scenario, we replaced the original X,Y coordinates with the two-dimensional UMAP coordinates, which represent a low-dimensional embedding that preserves the structure of the high-dimensional data, and replaced the PCs with the joint embedding from each integration method as input to GASTON.

### Computation platform

All experiments were conducted on a computer server equipped with Intel Xeon W-2195 CPUs, running at 2.3 GHz, featuring 25MB of L3 cache and 36 CPU cores. The server was configured with 256GB of DDR4 memory operating at 2,666MHz.

For GPU configurations, the same server was used, equipped with four Quadro RTX A6000 cards, each providing 48GB of memory and 4608 CUDA cores.

## Supplementary Information

Additional file 1: Supplementary Methods, Supplementary Notes, Supplementary Tables S1-S3, and Supplementary Figures S1-S38. Supplementary Notes include supplementary results.

## Data Availability

All code, tutorials, evaluation scripts, and related data files are freely available on GitHub https://github.com/maiziezhoulab/MaskGraphene [70] and on Zenodo with DOI: https://doi.org/10.5281/zenodo.16956499 under the MIT license [71]. All data and the corresponding annotation can be downloaded from https://benchmarkst-reproducibility.readthedocs.io/en/latest/Data%20availability.html and are described in Additional file 1: Table S1 with their sources. Dataset 1 consists of 12 human DLPFC sections, available at http://research.libd.org/spatialLIBD/ with manual annotation [72]. Dataset 2 includes five slices from the mouse hypothalamus available at https://datadryad.org/stash/dataset/doi:10.5061/dryad.8t8s248 with annotation [73]. Dataset 3 contains 33 consecutive mouse cerebral cortex tissue slices with similar shapes at https://zenodo.org/records/8167488 with annotation. Dataset 4 includes two slices of anterior and posterior mouse brain available at https://www.10xgenomics.com/ with annotation [74]. Dataset 5 is the mouse embryo dataset sequenced by Stereo-seq from the MOSTA project at https://db.cngb.org/stomics/mosta/resource/ with annotation. The simulation data is deposited in Zenodo https://zenodo.org/records/10800745.76. [75] Dataset 6 is the 10x Visium dataset for human breast cancer from the 10x Genomics database at https://support.10xgenomics.com/spatial-gene-expression/datasets/1.1.0/V1_Breast_Cancer_Block_A_Section_1 with annotation. Dataset 7 is the Drosophila embryo dataset by Stereo-seq at https://db.cngb.org/stomics/flysta3d/.
