# Supplementary material for: MaskGraphene: an advanced framework for interpretable joint representation for multi-slice, multi-condition spatial transcriptomics
Source: Genome Biol. 2025 Nov 5;26:380. doi: 10.1186/s13059-025-03850-w (PMC12590685; doi:10.1186/s13059-025-03850-w)
Supplement: Supplementary file 1 — Additional file 1: Supplementary Methods, Supplementary Notes, Supplementary Tables S1-S3, and Supplementary Figures S1-S38. Supplementary Notes include supplementary results. [file 13059_2025_3850_MOESM1_ESM.pdf]

MaskGraphene: an advanced framework for interpretable joint representation for multi-slice, multi-condition spatial transcriptomics

Hu et al.

Contents

1 Supplementary Methods **3**
S1 **Spot-to-spot mapping** . . . 3
S2 **Coordinate transformation via optimization for multi-slice integration** . . . 4

2 Supplementary Notes **5**
S1 **Comparison of STAligner embedding with MaskGraphene initial and final embeddings** . . . 5
S2 **Hyperparameter settings and sensitivity analysis for MaskGraphene** . . . 5

3 Supplementary Tables **7**
S1 Benchmark tools and real datasets . . . 8
S2 Proportional distribution of tissue structures at each embryo stage . . . 9
S3 Configurations of ablation study variants for MaskGraphene . . . 9

4 Supplementary Figures **10**
S1 Visualization plots for alignment-misalignment-unalignment and spot-to-spot mapping ratio on DLPFC 151671-151672 and 151675-151676 pairs . . . 11
S2 Visualization plots for alignment-misalignment-unalignment and spot-to-spot mapping ratio on MHypo Bregma -0.14 - -0.19 pair . . . 12
S3 Bar plots of Layer-wise alignment accuracy for ablation study . . . 13
S4 Spot-to-spot mapping ratio on DLPFC and MHypo datasets for ablation study . . . 14
S5 Bar plots of layer-wise alignment accuracy by MaskGraphene, MaskGraphene-PASTE, and PASTE . . . 15
S6 Visualization plots for alignment-misalignment-unalignment and spot-to-spot mapping ratio on DLPFC dataset by MaskGraphene, MaskGraphene-PASTE, and PASTE . . . 16
S7 Visualization plots for alignment-misalignment-unalignment and spot-to-spot mapping ratio on MHypo dataset by MaskGraphene, MaskGraphene-PASTE, and PASTE . . . 17
S8 UMAP plots of low dimensional joint embedding on DLPFC four-slice integration (151673-151674-151675-151676). . . . 18
S9 UMAP plots of low dimensional joint embedding on DLPFC pairwise integration. 19
S10 UMAP plots of low dimensional joint embedding on MHypo pairwise integration 20
S11 UMAP plots of low dimensional joint embedding and boxplots of evaluation metrics for the DLPFC dataset, comparing MaskGraphene and MaskGraphene-PASTE 21
S12 Boxplots of evaluation metrics from ablation studies on the DLPFC and MHypo datasets . . . 22
S13 Topography analysis based on STAligner embeddings after DLPFC 151673-151674 pairwise integration . . . 23

S14 Topography analysis based on GraphST embeddings after DLPFC 151673-151674 pairwise integration . . . 24
S15 Topography analysis based on PCA-derived embeddings from individual DLPFC slice 151673 . . . 25
S16 Topography analysis based on MaskGraphene embeddings after DLPFC four-slice integration (151673-151674-151675-151676) . . . 26
S17 Topography analysis based on MaskGraphene embeddings of DLPFC 151673 . . . 27
S18 Biomarker and topography analysis after brain slice integration . . . 28
S19 Biomarker analysis after breast cancer pairwise integration . . . 29
S20 UMAP visualizations of low-dimensional joint embeddings generated by STAligner and GraphST on the Drosophila dataset. . . . 30
S21 Spatial visualization of joint domain identification after DLPFC 151507-151508 pairwise integration . . . 31
S22 Spatial visualization of joint domain identification after DLPFC 151671-151672 pairwise integration . . . 32
S23 Spatial visualization of joint domain identification after DLPFC 151673-151674 pairwise integration . . . 33
S24 Spatial visualization of joint domain identification after four-slice integration (151507-151508-151509-151510) . . . 34
S25 Spatial visualization of joint domain identification after MHypo Bregma -0.19 - -0.24 pairwise integration . . . 35
S26 Spatial visualization of joint domain identification after MHypo five-slice integration 36
S27 UMAP, PAGA, and biomarker analysis of MaskGraphene (coordinate transformation) after multi-slice integration . . . 37
S28 Spatial visualization of joint domain identification and ARI boxplots by MaskGraphene (coordinate transformation) after multi-slice integration . . . 38
S29 UMAP plots of low dimensional joint embedding on the MB dataset . . . 39
S30 Spatial visualizations and biomarker analysis on the mouse embyro dataset . . . 40
S31 Visualization of marker genes from the integrated analysis . . . 41
S32 Runtime comparison of integration methods across datasets . . . 42
S33 Grid search analysis of graph model layer configurations in MaskGraphene on the DLPFC and MHypo datasets . . . 43
S34 Grid search analysis of masking and re-masking rates in MaskGraphene on the DLPFC and MHypo datasets . . . 44
S35 Grid search analysis of hyperparameters in MaskGraphene on the DLPFC and MHypo datasets . . . 45
S36 Boxplots of ARI for pairwise and multi-slice integration using MaskGraphene-initial, MaskGraphene-final, and STAligner on the DLPFC and MHypo datasets 46
S37 Boxplots of iLISI, Isometry correlation, and Procrustes dissimilarity for multi-slice integration on the DLPFC and MHypo datasets . . . 47
S38 ARI box plots of clustering results after integration with varying random seeds across different datasets and methods . . . 48

1 Supplementary Methods

S1 Spot-to-spot mapping

To enhance inter-slice connections, we developed a cluster-wise optimal transport (OT)-based local alignment method to establish reliable spot-to-spot (node-to-node) mapping (alignment) across slices, enabling the unification of all spots into a shared coordinate system for constructing the inter-slice k-NN graph in MaskGraphene's graph model during Steps 4-5 of the final integration. This cluster-wise alignment process begins with the previous initial integration step, which performs joint clustering to identify shared clusters across slices. Once shared clusters are identified across slices, cluster-wise OT-based local alignment is performed. Specifically, for each pair of shared clusters (representing the same domain across each pair of slices), Following prior work on spatial slice alignment (e.g., PASTE [31]), we adopt a standard Gromov-Wasserstein optimal transport (GW-OT) formulation to compute probabilistic couplings between spots and derive spot-to-spot mappings. Unlike PASTE, which solves a single global fused GW problem per adjacent slice pair, MaskGraphene solves multiple cluster-wise GW problems over matched domains across all slices. Aggregating these local alignments provides global context while improving robustness to partial overlap and localized deformations. Each cluster-wise GW problem is optimized with an iterative conditional gradient method [66].

Given H slices that have M shared clusters identified from the initial clustering. let $(X^{h_m}, D^{h_m}, g^{h_m})$ represent the spots in the cluster m of the slice h, where $X^{h_m} = [x_{ij}^{h_m}] \in \mathbb{N}^{p \times n^{h_m}}$ is a p genes by n^{h_m} spots transcript count matrix, $x_{ij}^{h_m}$ is the transcript count for gene i in spot j, $D^{h_m} = [d_{ij}^{h_m}] \in \mathbb{R}_+^{n^{h_m} \times n^{h_m}}$ is a n^{h_m} spots by n^{h_m} spots spatial distance matrix, $d_{ij}^{h_m} = ||z_{.i}^{h_m} - z_{.j}^{h_m}||$ is the spatial distance between spots i and j, $z_{.i}^{h_m}$ is the 2D coordinate of spot i, and $g^{h_m} = (g_1^{h_m}, \cdots, g_{n^{h_m}}^{h_m})$ is a weight vector that $g_i^{h_m} > 0$ is the weight of spot i.

For each shared cluster m across all the slices, the transport cost is minimized as follows to find a mapping between slices:

$$\begin{aligned}
\min_{\{\Pi^{h_m}\}} \quad & \sum_{h=1}^{H-1} F\Big(\Pi^{h_m};\, X^{h_m},\, D^{h_m},\, g^{h_m},\, X^{(h+1)_m},\, D^{(h+1)_m},\, g^{(h+1)_m},\, c,\, \alpha\Big) \\
\text{s.t.} \quad & \sum_{j=1}^{n^{(h+1)_m}} \pi_{ij}^{h_m} = g_i^{h_m} \quad {}_{\substack{i=1,\dots,n^{h_m} \\ h=1,\dots,H-1}}, \\
& \sum_{i=1}^{n^{h_m}} \pi_{ij}^{h_m} = g_j^{(h+1)_m} \quad {}_{\substack{j=1,\dots,n^{(h+1)_m} \\ h=1,\dots,H-1}}, \\
& \pi_{ij}^{h_m} \geq 0 \quad {}_{\substack{\forall\, i,j \\ h=1,\dots,H-1}}.
\end{aligned} \tag{1}$$

where

$$\begin{aligned}
& F\Big(\Pi^{h_m};\, X^{h_m},\, D^{h_m},\, g^{h_m},\, X^{(h+1)_m},\, D^{(h+1)_m},\, g^{(h+1)_m},\, c,\, \alpha\Big) \\
& \quad = (1-\alpha) \sum_{i,j} c(x_{.i}^{h_m}, x_{.j}^{(h+1)_m}) \pi_{ij} + \alpha \sum_{i,j,k,l} (d_{ik}^{h_m} - d_{jl}^{(h+1)_m})^2 \pi_{ij} \pi_{kl}.
\end{aligned}$$

If there is no prior information about the spots, uniform distributions $g_i^{h_m} = \frac{1}{n^{h_m}}$ and $g_j^{(h+1)_m} = \frac{1}{n^{(h+1)_m}}$ are assigned. $\Pi^{h_m} = [\pi_{ij}^{h_m}]$ is the transport plan between slices h and $h+1$ in cluster m, i, k refer to spots in slice h, j, l refer to spots in slice $h+1$, c is the expression cost function, and $\alpha \in [0,1]$ is a weight.

The complete mapping (alignment) Π between the two slices h and $h+1$ is thus obtained by combining the transport plans (weights) across all clusters, with spots ordered according to their cluster labels:

$$\Pi^h = [\pi_{ij}^h] = \begin{bmatrix} \Pi^{h_1} & 0 & 0 & \cdots & 0 & \cdots & 0 \\ 0 & \Pi^{h_2} & 0 & \cdots & 0 & \cdots & 0 \\ 0 & 0 & \cdots & \Pi^{h_m} & \cdots & 0 & 0 \\ \vdots & \vdots & \cdots & \vdots & \cdots & \vdots & \vdots \\ 0 & 0 & 0 & \cdots & 0 & \cdots & \Pi^{h_M} \end{bmatrix}. \tag{2}$$

where $\Pi^{h_m} = [\pi_{ij}^{h_m}]$ is the transport plan (weight) between slices h and $h+1$ in cluster m. By deriving shared clusters from an initial integration of all slices and assembling Π from multiple small, cluster-wise GW problems (across all adjacent slice pairs), our local mapping is globally informed by the entire set of slices.

S2 Coordinate transformation via optimization for multi-slice integration

Coordinate transformation via optimization: building on the local shared clusters (domains) identified during initial integration, we perform spatial coordinate transformation across multiple slices by predicting their coordinate systems using a mutual nearest neighbor (MNN) graph constructed from the unified spatial coordinates of the spots. The process is framed as an optimization problem guided by an objective function. To address partially overlapping slices during multi-slice integration, a penalty term is incorporated to account for the proportion of overlapping spots. Using the differential evolution optimization algorithm [68], we determine the optimal transformation parameters - including translation vectors and rotation angles - for each slice. These parameters are then used to transform the coordinates of each slice.

The transformation process of slice j is expressed mathematically as:

$$S'_j = RMS_j + T \tag{3}$$

where S_j represents the original slice's coordinate matrix, S'_j is the transformed coordinate matrix, R is the rotation matrix, M is the mirroring matrix, and T is the translation matrix.

Once the shared domain (cluster) information for all spots in the two adjacent slices $j-1$ and j is obtained from our initial integration and joint clustering, identify a rotation matrix R and a translation matrix T for slice j that maximize the following objective function [33]:

$$F(R, M, T; S_j, S_{j-1}) = \frac{1}{n_o} \sum_{l'_j \in O_j} \frac{1}{m_{l'_j}} \sum_{l_{j-1} \in \langle l'_j \rangle_k^{j-1}} \delta\left[\text{domain}(l_{j-1}), \text{domain}(l'_j)\right] + f\left(\frac{n_o}{n_j}\right) \tag{4}$$

The first term measures the normalized proportion of overlapping spots in the transformed slice j whose k-nearest neighbors in slice $j-1$ both satisfy the distance threshold and, together with the overlapping spots themselves, belong to the same domain (cluster), promoting biological consistency in the coordinate transformation. n_j denotes the number of spots in slice j, l'_j represents the overlapping spots in the transformed slice j whose Euclidean distance to their first mutual nearest neighbor spots in slice $j-1$ is less than a defined maximum distance. By default, the maximum distance is set to the median Euclidean distance between spots in slice $j-1$ and their $2k$th nearest neighbor of spots found in the same slice $j-1$. The set of those overlapping spots is denoted as O_j, and the total number of spots in O_j is n_o. The term $\langle l'_j \rangle_k^{j-1}$ represents the set of spots in slice $j-1$ that are k-nearest neighbors of spots l'_j, with Euclidean distance to l'_j less than the maximum distance. The number of spots in $\langle l'_j \rangle_k^{j-1}$ is $m_{l'_j}$. The Kronecker delta function, $\delta(x, y)$, checks whether each overlapping spot in the transformed slice j and its k-nearest neighboring spots within the distance threshold in slice $j-1$ belong to the same domain (cluster), returning 1 if they do and 0 otherwise. We set k to 15 by default, corresponding to approximately one to two hops in the k-nearest neighbor graph - a range commonly used in ST integration to balance local neighborhood resolution with noise robustness.

The parameter p controls how sensitive the penalty function $f(x) = -(1-x)^p$ is to the overlap ratio $f(\frac{n_o}{n_j})$. Large p values make the penalty more responsive to small decreases in overlap, while smaller values keep it smoother. We set $p = 2$ by default because quadratic penalties are commonly used in machine learning for their balance between stability and sensitivity. This choice provides smooth penalization without the abrupt effects seen at higher powers. Together, these terms encourage both domain label agreement and sufficient geometric overlap during coordinate transformation.

We employ the differential evolution algorithm [63] to optimize the transformation matrices $\{R, M, T\}$, maximizing the objective function $F(R, M, T; S_j, S_{j-1})$ for all adjacent slice pairs, thereby achieving coordinate transformation across slices.

2 Supplementary Notes

S1 Comparison of STAligner embedding with MaskGraphene initial and final embeddings

We systematically compared STAligner, MaskGraphene-initial, and MaskGraphene-final embeddings on the DLPFC and MHypo datasets under both pairwise and multi-slice configurations, evaluating the performance of STAligner relative to MaskGraphene's initial and final embeddings. As measured by Adjusted Rand Index (ARI), MaskGraphene-initial consistently outperformed STAligner (Additional file 1: Figs. S36-37), indicating improved clustering accuracy during the initial integration phase. This aligns with its intended role of enhancing shared cluster or domain identification across slices, which provides the foundation for subsequent cluster-wise local alignment. Specifically, in DLPFC four-slice and MHypo five-slice integrations, the average ARI improved from $\sim$0.54 to $\sim$0.55 and from $\sim$0.38 to $\sim$0.53, respectively, when using MaskGraphene-initial over STAligner. This performance was further enhanced in the final embeddings, which incorporate hard-links, reaching $\sim$0.63 for DLPFC and $\sim$0.54 for MHypo. Similar trends were observed in the pairwise integrations, with MaskGraphene-final achieving the highest ARI scores in all settings.

Beyond clustering accuracy, both MaskGraphene-initial and MaskGraphene-final embeddings demonstrated strong integration performance. As shown in Additional file 1: Figs. S36-37, MaskGraphene-final consistently exhibited superior batch mixing and geometry preservation across most settings, except in iLISI and isometry correlation for the DLPFC four-slice integration. In this setting, MaskGraphene-initial achieved the highest iLISI and isometry correlation, indicating improved batch mixing and geometric alignment. MaskGraphene-initial also outperformed STAligner across most settings, with the exception of Procrustes dissimilarity in the DLPFC four-slice integration and iLISI in the MHypo five-slice integration.

These results highlight the complementary benefits of the two stages of integration. The initial embeddings, learned without hard-links, are optimized for enhancing domain identification across slices. The final embeddings, refined with alignment-derived hard-links, improve geometric coherence and spatial interpretability. Together, they illustrate how progressive integration in MaskGraphene balances biological signal preservation with spatial alignment fidelity.

S2 Hyperparameter settings and sensitivity analysis for MaskGraphene

The performance and reliability of MaskGraphene can be influenced by key hyperparameter choices, including network dimensionalities, loss weight coefficients, scaling factors, and masking strategies. It is important to assess whether MaskGraphene's performance is stable across different parameter configurations. To address this, we documented the default hyperparameters used in our framework and systematically investigated the sensitivity of model outputs to variations in these settings, ensuring that the observed performance gains are not dependent on finely tuned parameters but instead reflect a robust and generalizable integration approach.

MaskGraphene's default configuration employs a structural feature dimension of 512 and an embedding dimension of 32, with both the scaling factor (γ) and balancing coefficient (λ) set to 1 (i.e., $\gamma = 1$, $\lambda = 1$). To adapt to differences in data quality across spatial transcriptomics platforms, we adopt adaptive masking and remasking strategies: high-quality datasets such as MHypo and MB (MERFISH) use more aggressive rates of 0.5/0.5, while lower-coverage datasets (e.g., 10x Visium and Stereo-seq) use more conservative settings of 0.1/0.1. These values are empirically chosen to enhance generalization on high-quality datasets, where modest improvements in performance metrics are observed, while avoiding excessive over-optimization that could overshadow the core methodological contributions of MaskGraphene.

We further conducted a comprehensive grid search to evaluate the robustness of the model to hyperparameter variations, testing a wide range of configurations:

- Embedding / Structural dimensions: 256/16, 256/32, 512/32, 1024/16, 1024/32, 256/64, 512/64, 1024/64
- Loss weights: $\lambda \in \{0.5, 1, 2\}$, $\gamma \in \{1, 2, 3\}$
- Masking / Remasking rates:

– For DLPFC: {5%, 10%, 20%}
– For MHypo: {40%, 50%, 60%}

Experiments conducted on representative datasets (DLPFC pairwise and four-slice, MHypo pairwise and five-slice) demonstrated that MaskGraphene's performance remained stable across these hyperparameter ranges (Additional file 1: Figs. S33-35). While certain combinations yielded slight improvements in metrics such as layer-wise alignment accuracy, spot-to-spot matching ratio, iLISI, Isometry correlation, and Procrustes dissimilarity, overall performance trends were consistent and robust. These results highlight the stability of MaskGraphene without the need for extensive hyperparameter tuning.

3 Supplementary Tables

Algorithm	Language	Resource	Output	Method	Link
MaskGraphene	Python	Hu et al. 2025	Clustering Labels<br>Embedding	Graph Autoencoder<br>Self-supervised Learning<br>Cluster-wise Alignment	https://github.com/maiziezhoulab/MaskGraphene
SpaMask	Python	Min et al. 2025 [29]	Clustering Labels<br>Embedding	Graph Neural Network<br>Self-supervised Learning	https://github.com/wenwenmin/SpaMask
SpaDo	R	Duan et al. 2024 [28]	Clustering Labels<br>Embedding	kNN<br>Multi-slice Clustering	https://github.com/bm2-lab/SpaDo
SPIRAL	Python	Guo et al. 2023 [26]	Clustering Labels<br>Refined Coordinates<br>Embedding	GraphSAGE<br>Optimal Transport	https://github.com/guott15/SPIRAL
STAligner	Python	Zhou et al. 2023 [27]	Clustering Labels<br>Embedding	Graph Autoencoder<br>Attention Mechanism<br>Triplet Loss	https://github.com/zhanglabtools/STAligner
PRECAST	R	Liu et al. 2023 [24]	Clustering Labels<br>Embedding	Gaussian Mixture Model<br>Hidden Markov Field	https://github.com/cran/PRECAST
BASS	R	Li et al. 2022 [23]	Clustering Labels	Bayesian Analysis<br>Multi-sample Analysis	https://github.com/zhengli09/BASS
DeepST	Python	Xu et al. 2022 [20]	Clustering Labels<br>Embedding	Data Augmentation<br>Variational Autoencoder	https://github.com/JiangBioLab/DeepST
GraphST	Python	Long et al. 2022 [25]	Clustering Labels<br>Embedding	Graph Neural Network<br>Contrastive Learning	https://github.com/JinmiaoChenLab/GraphST

ST Dataset	Abbrev.	Protocol	Spots/Genes	Slices	Source
Human DLPFC [73]	DLPFC	10x Visium	3431–4788 / 33538	12	http://spatial.libd.org/spatialLIBD/
Mouse Hypothalamus [74]	MHypo	MERFISH	5488–5926 / 155	5	https://datadryad.org/stash/dataset/doi:10.5061/dryad.8t8s248
Mouse Brain [49]	MB	MERFISH	2033–5624 / 254	10	https://zenodo.org/records/8167488
Mouse Brain Section 2 [75]	MB2SA&P	10x Visium	2695, 3353 / 32285	2	https://www.10xgenomics.com/resources/datasets/mouse-brain-serial-section-2-sagittal-anterior-1-standard
MOSTA Embryo [51]	mouse embryo	Stereo-seq	6 slices	2	https://db.cngb.org/stomics/mosta/resource/
Breast Cancer [69]	BC	10x Visium	2 slices	2	https://support.10xgenomics.com/spatial-gene-expression/datasets/1.1.0/V1_Breast_Cancer_Block_A_Section_1
Drosophila Embryo [47]	Drosophila embryo	Stereo-seq	16 slices	2	https://en.stomics.tech/applications/devenlopment-research/index.html

Table S1: **Benchmark tools and real datasets. Top panel**: Summary of methods benchmarked in this work including programming language, core method, and links. **Bottom panel**: Summary of spatial transcriptomics datasets used, including protocol, spot/gene counts, and sources.

Tissue type	Ground Truth E11.5	Ground Truth E12.5	MaskGraphene E11.5	MaskGraphene E12.5	STAligner E11.5	STAligner E12.5	Primary germ layers
**Brain**	19.20%	22.40%	14.20% (-26.04%)	13.20% (-41.07%)	6.00% (-68.75%)	7.50% (-66.52%)	Ectoderm
**Dorsal root ganglion**	2.90%	2.60%	2.60% (-10.34%)	2.30% (-11.54%)	2.00% (-31.03%)	2.00% (-23.08%)	
**Jaw and tooth**	3.60%	5.20%	2.80% (-22.22%)	5.00% (-3.85%)	1.70% (-52.78%)	3.10% (-40.38%)	
**Meninges**	3.20%	10.40%	5.90% (+84.37%)	7.20% (-30.77%)	5.40% (+68.75%)	7.20% (-30.77%)	
**Heart**	5.70%	3.00%	3.70% (-35.09%)	3.80% (+26.67%)	3.90% (-31.58%)	3.00% (0.00%)	Mesoderm
**Urogenital ridge**	5.90%	2.00%	0.00% (-100.00%)	0.00% (-100.00%)	3.50% (-40.68%)	2.70% (+35.00%)	
**Blood vessel**	2.60%	2.60%	4.20% (+61.54%)	3.00% (+15.38%)	5.50% (+111.54%)	2.00% (-23.08%)	Endoderm
**Connective tissue**	4.90%	5.30%	3.40% (-30.61%)	3.20% (-39.62%)	5.70% (+16.33%)	5.90% (+11.32%)	
**GI tract**	5.00%	2.90%	9.70% (+94.00%)	4.50% (+55.17%)	12.60% (+152.00%)	12.60% (+334.48%)	
**Liver**	2.50%	2.80%	2.20% (-12.00%)	2.90% (+3.57%)	2.30% (-8.00%)	3.30% (+17.86%)	
**Lung primordium**	0.70%	1.10%	4.50% (+542.86%)	3.10% (+181.82%)	0.00% (-100.00%)	0.00% (-100.00%)	
**Cavity**	7.70%	12.50%	8.30% (+7.79%)	8.00% (-36.00%)	2.20% (-71.43%)	4.60% (-63.20%)	NA

Table S2: **Proportional distribution of tissue structures at each embryo stage.** Proportion of spots for each structure relative to the total spots at time points E11.5 and E12.5, based on the ground truth and the integration results from MaskGraphene and STAligner. The ratios in parentheses represent the proportional differences of each tool compared to the ground truth.

**Variant**	**Hard Link**	**Masked Loss**	**Graph**	**Triplet Loss**	**Latent Loss**	**Notes**
MaskGraphene-Full	✓	✓	✓	✓	✓	Full model
MaskGraphene-MLP	–	–	–	✓	–	MLP-only baseline
MaskGraphene-GAE	✓	–	✓	✓	–	Standard GAE baseline
MaskGraphene-HardlinkOnly	✓	✓	✓	–	✓	Impact of removing triplet
MaskGraphene-MaskOnly	✓	✓	✓	–	–	Masking without auxiliary loss
MaskGraphene-SoftlinkOnly	–	✓	✓	✓	✓	Graph with soft links only

Table S3: **Configurations of ablation study variants for MaskGraphene.** Each variant selectively disables specific components - such as hard link, masked reconstruction loss, graph structure, triplet loss, or latent consistency loss - to evaluate their individual contributions to the full model.

4 Supplementary Figures

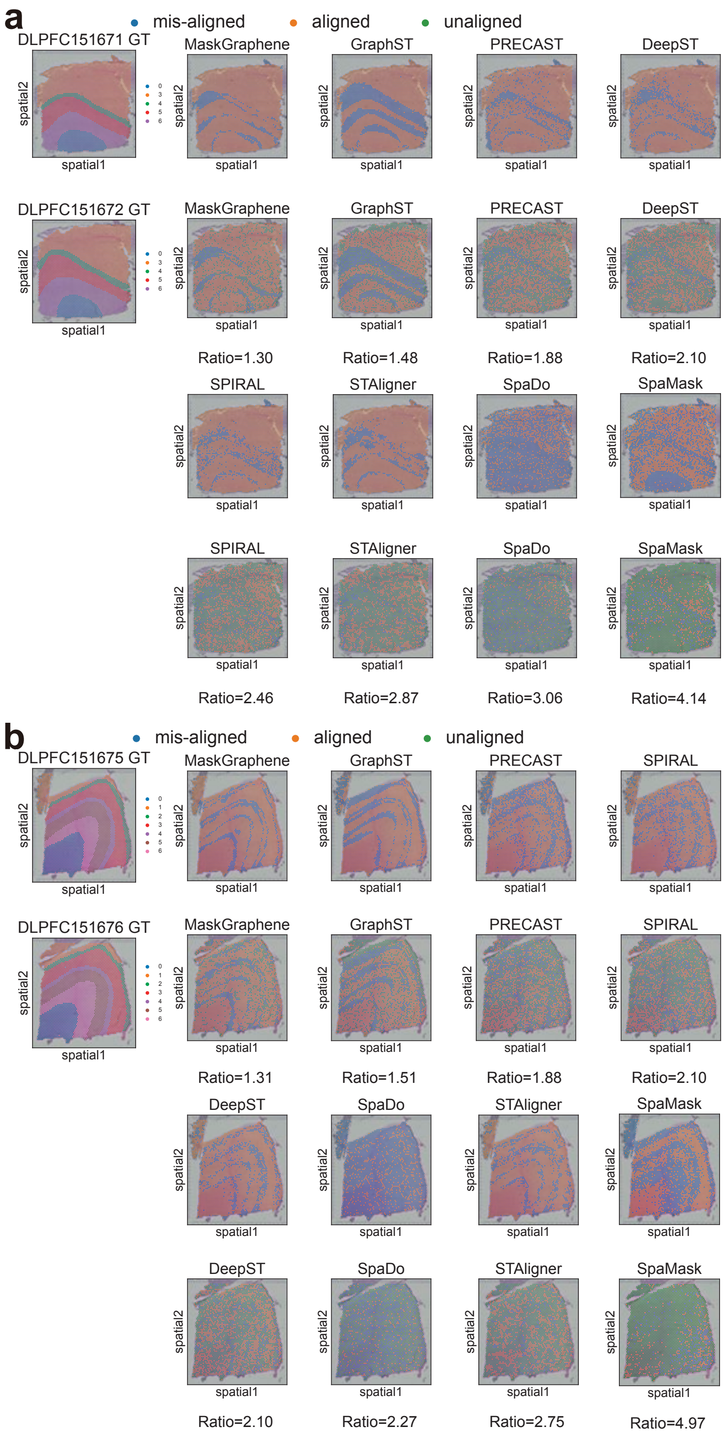

Fig. S1: **Visualization plots for alignment-misalignment-unalignment and spot-to-spot mapping ratio on DLPFC 151671-151672 and 151675-151676 pairs.** **(a-b)** Visualization plots displaying aligned spots, misaligned spots, and unaligned spots during the alignment process. The anchor spot from the first (top) slice is aligned to the corresponding spots on the second (bottom) slice for DLPFC 151671-151672 pair **(a)** and DLPFC 151675-151676 pair **(b)**. The first slice pair illustrates ground truth (GT) annotations. The values below each plot indicate the spot-to-spot matching ratio.

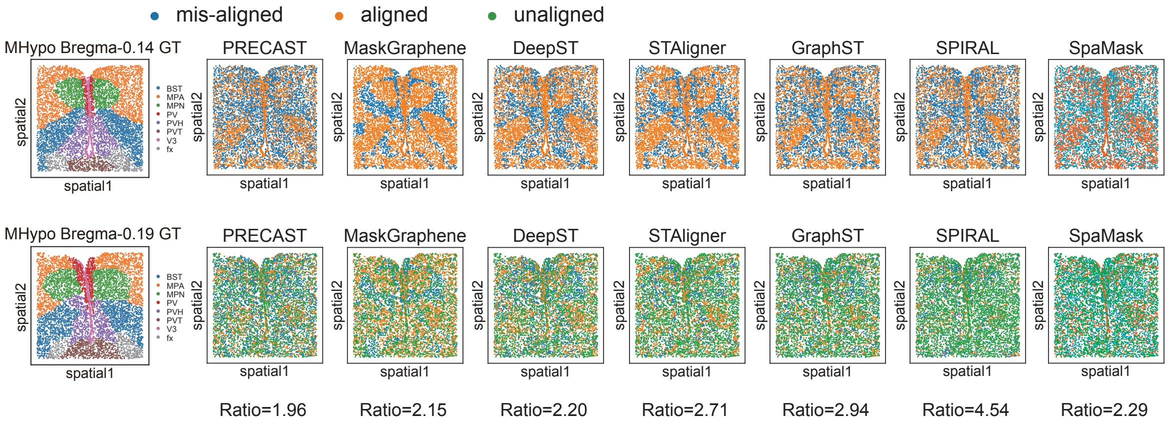

Fig. S2: **Visualization plots for alignment-misalignment-unalignment and spot-to-spot mapping ratio on MHypo Bregma -0.14 - -0.19 pair.** Visualization plots displaying aligned spots, misaligned spots, and unaligned spots during the alignment process. The anchor spot from the first (top) slice is aligned to the corresponding spots on the second (bottom) slice for MHypo Bregma -0.14 - -0.19 pair. The first slice pair illustrating ground truth (GT) annotations. The values below each plot indicate the spot-to-spot matching ratio.

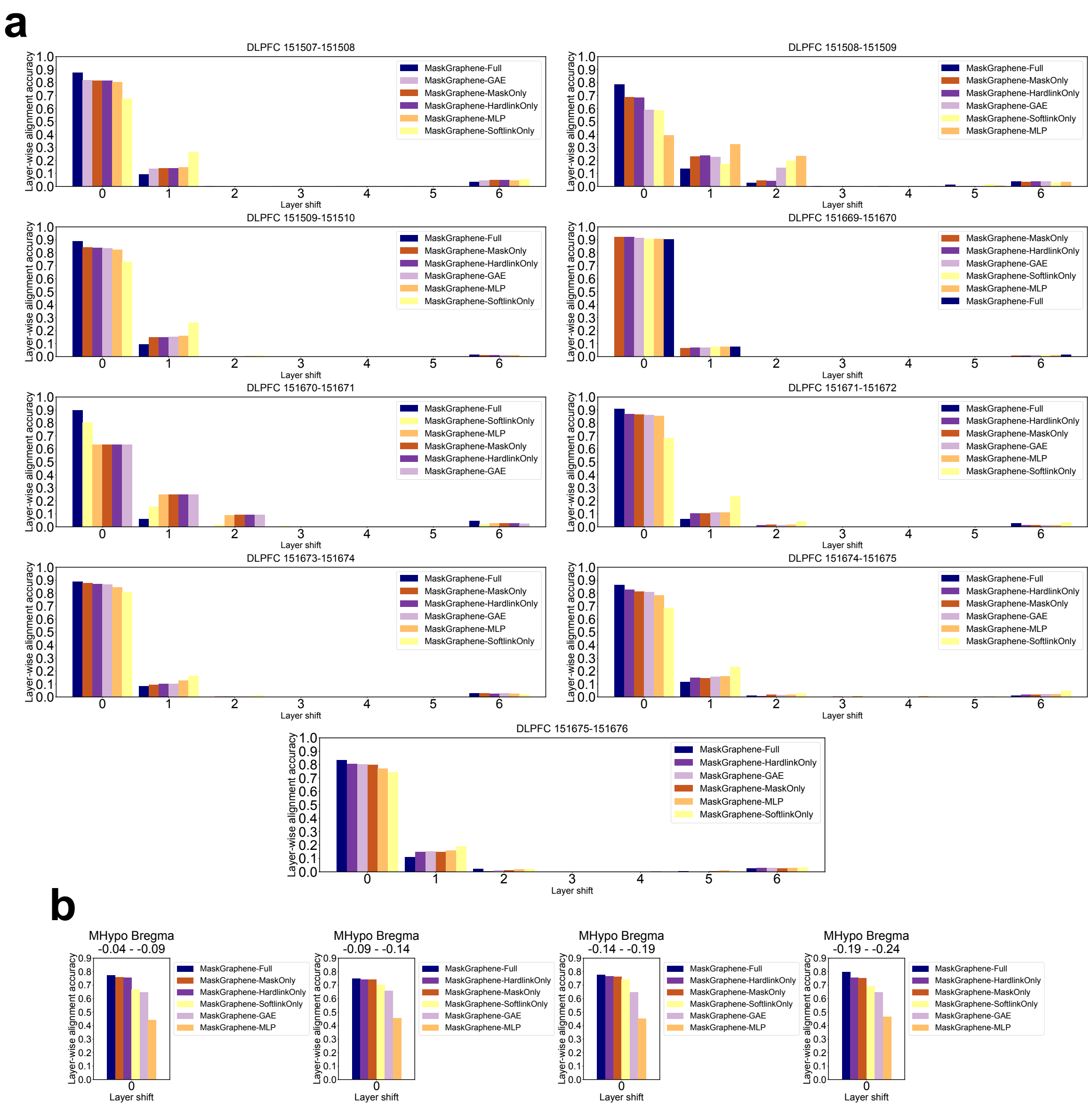

Fig. S3: **Bar plots for layer-wise alignment accuracy on DLPFC and MHypo datasets for ablation study.** **(a)** Bar plots illustrating the layer-wise alignment accuracy across layer shifts ranging from 0 to 6, comparing seven various methods on nine DLPFC slice pairs. **(b)** Bar plots illustrating the layer-wise alignment accuracy for a layer shift of 0, comparing seven various methods on four MHypo slice pairs. Tools are sorted in descending order based on the accuracy for layer shift of 0 in **(a, b)**.

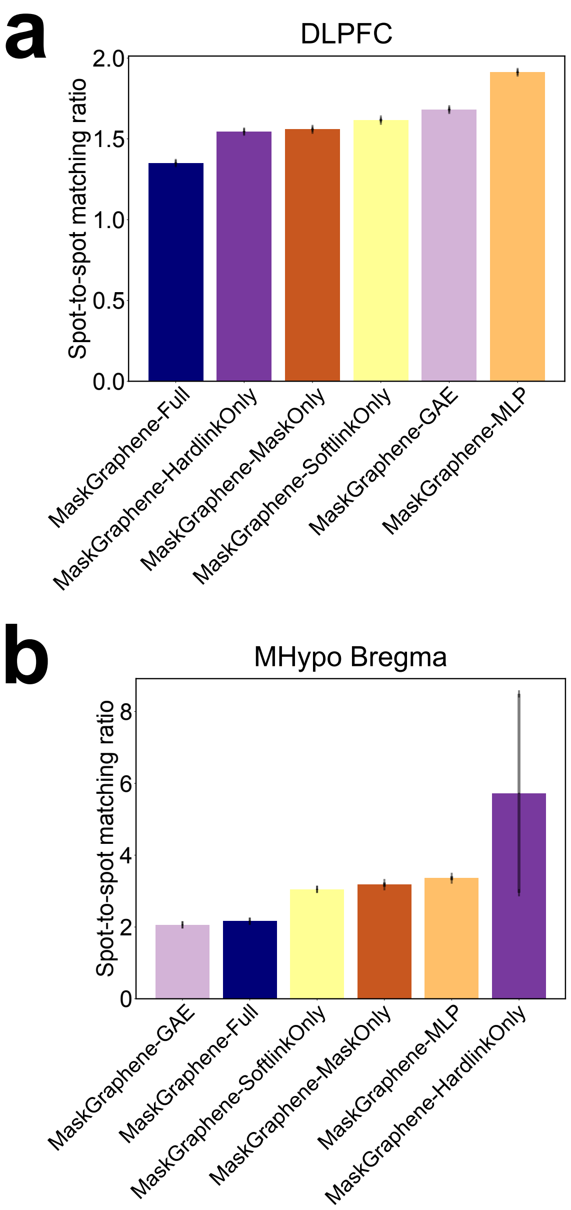

Fig. S4: **Spot-to-spot mapping ratio on DLPFC and MHypo datasets for ablation study. (a-b) Bar plots representing the average spot-to-spot mapping ratio of each model variant on two datasets: DLPFC (a) and MHypo (b).**

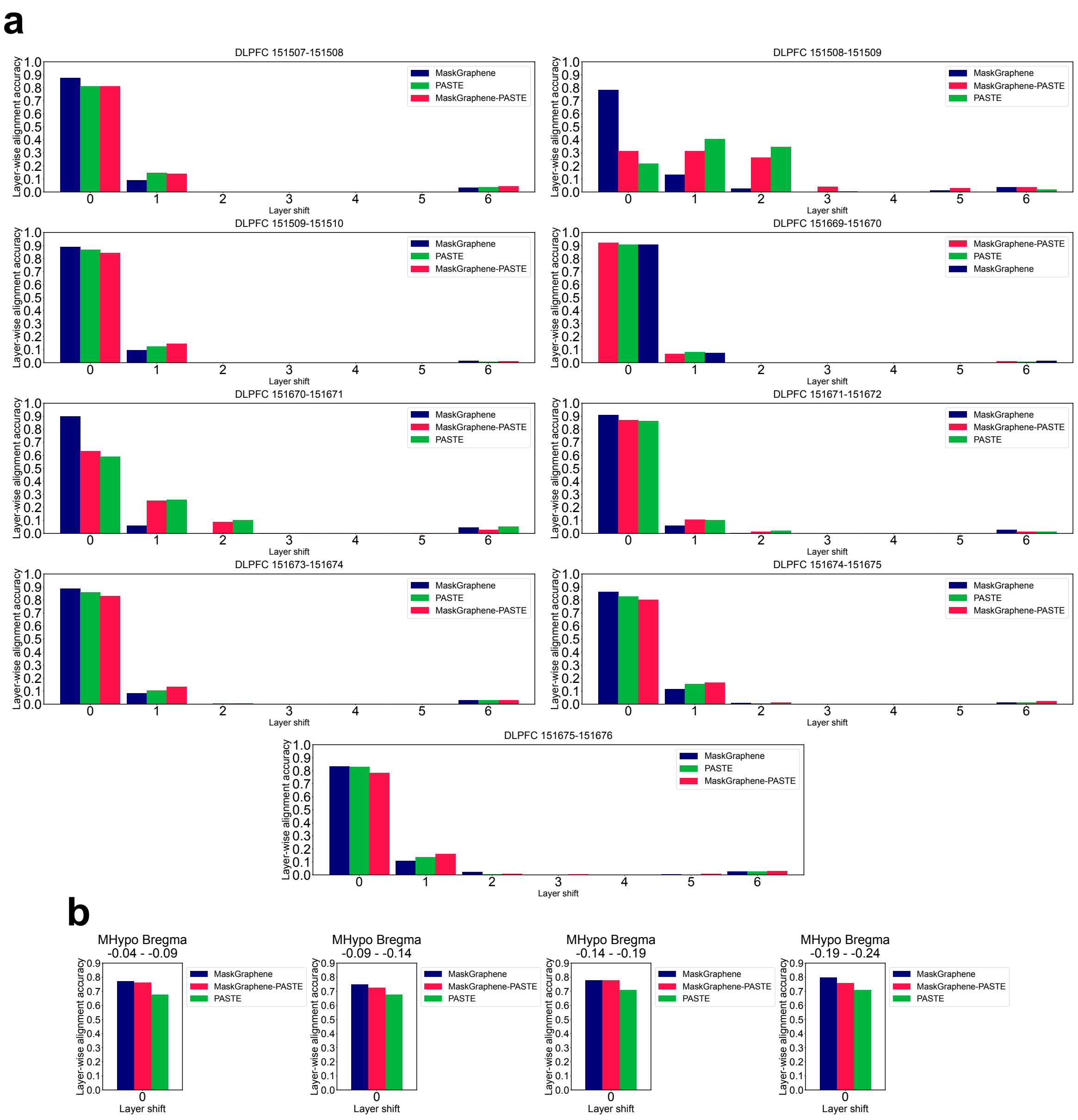

Fig. S5: **Bar plots for layer-wise alignment accuracy on DLPFC and MHypo datasets by MaskGraphene, MaskGraphene-PASTE, and PASTE. (a)** Bar plots illustrating the layer-wise alignment accuracy across layer shifts ranging from 0 to 6, comparing three various methods on nine DLPFC slice pairs. **(b)** Bar plots illustrating the layer-wise alignment accuracy for a layer shift of 0, comparing three various methods on four MHypo slice pairs. Tools are sorted in descending order based on the accuracy for layer shift of 0 in **(a, b)**.

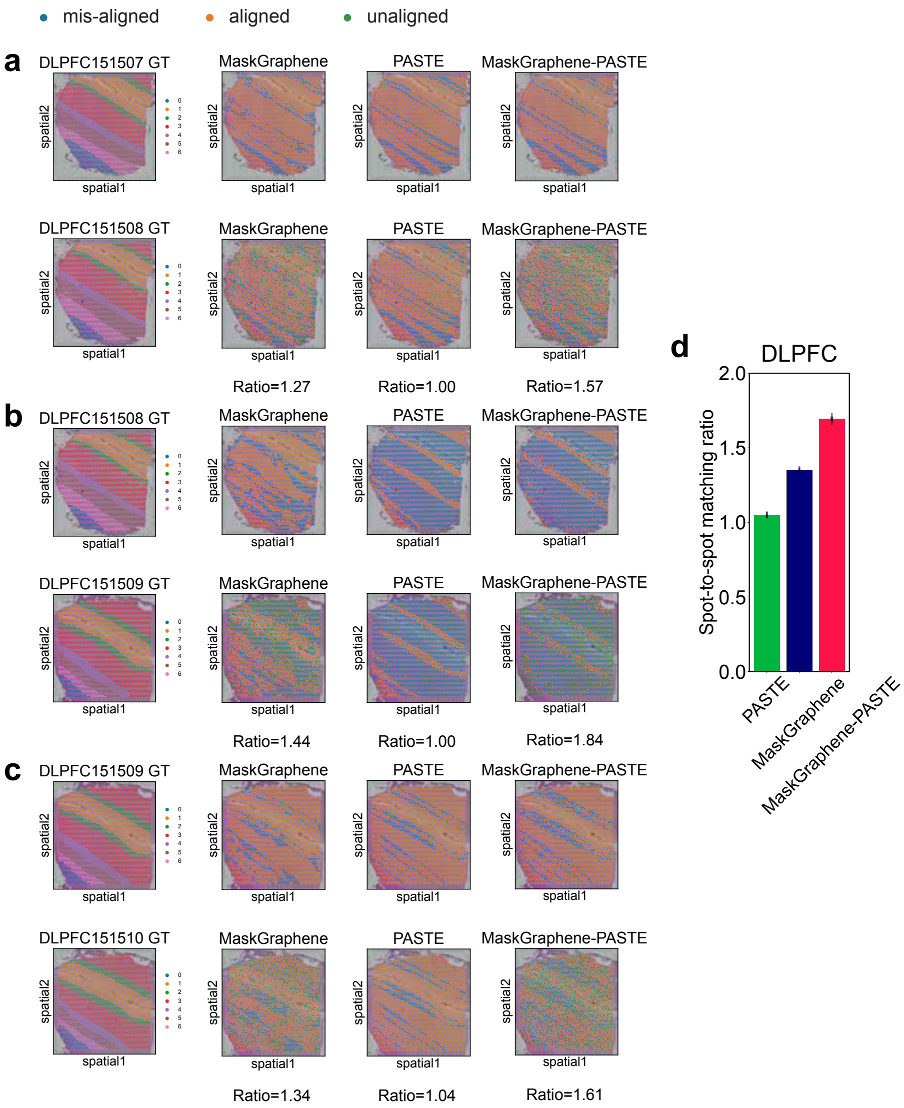

Fig. S6: **Visualization plots for alignment-misalignment-unalignment and spot-to-spot mapping ratio on DLPFC dataset by MaskGraphene, MaskGraphene-PASTE, and PASTE. (a-c)** Visualization plots displaying aligned spots, misaligned spots, and unaligned spots during the alignment process. The anchor spot from the first (top) slice is aligned to the corresponding spots on the second (bottom) slice for DLPFC 151507-151508 pair **(a)**, DLPFC 151508-151509 pair **(b)**, and DLPFC 151509-151510 pair **(c)**. The first slice pair illustrating ground truth (GT) annotations. The values below each plot indicate the spot-to-spot matching ratio. **(d)** Bar plots representing the average spot-to-spot mapping ratio of each tool on DLPFC dataset.

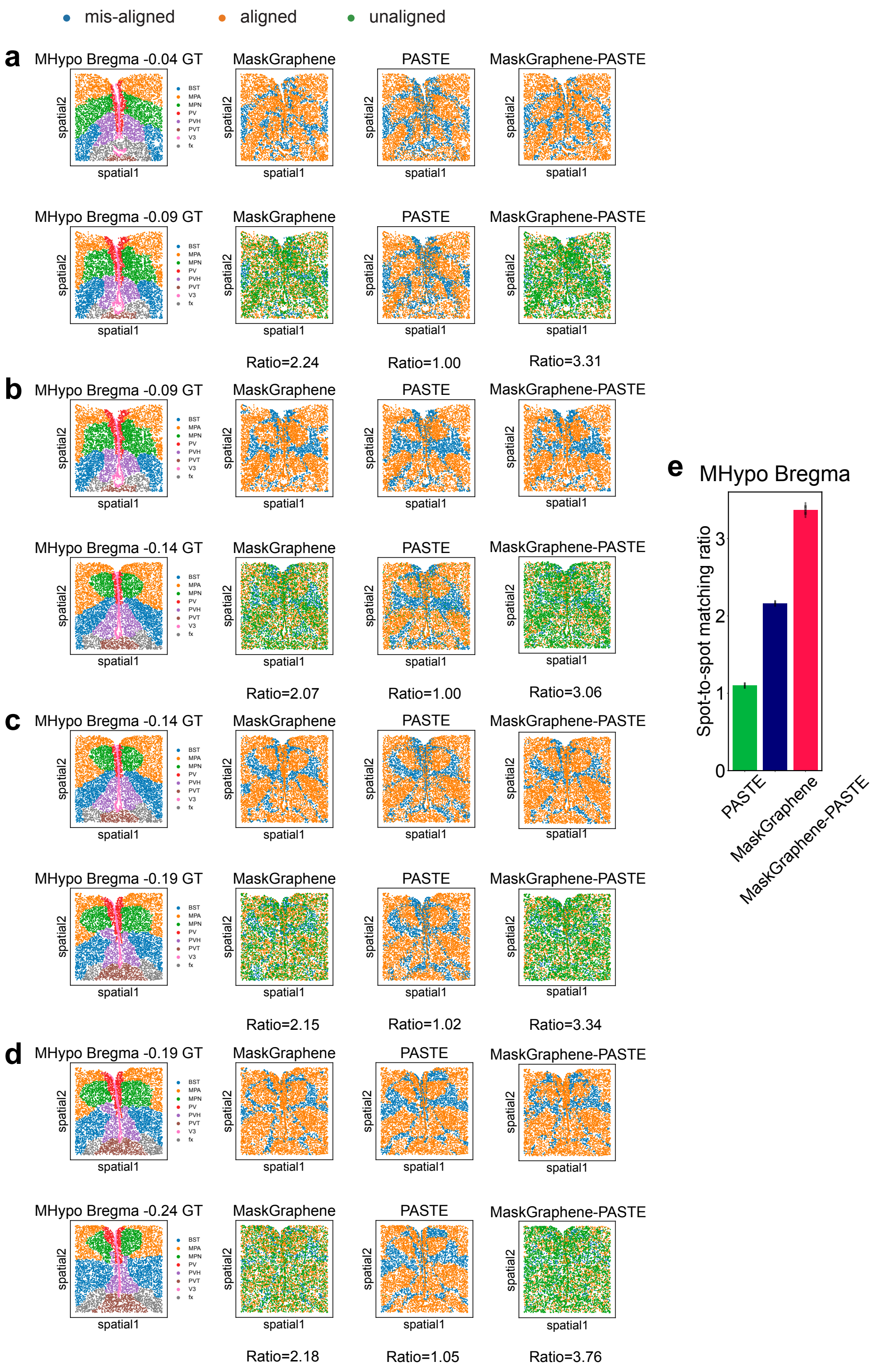

Fig. S7: **Visualization plots for alignment-misalignment-unalignment and spot-to-spot mapping ratio on MHypo dataset by MaskGraphene, MaskGraphene-PASTE, and PASTE. (a-d)** Visualization plots displaying aligned spots, misaligned spots, and unaligned spots during the alignment process. The anchor spot from the first (top) slice is aligned to the corresponding spots on the second (bottom) slice for MHypo Bregma -0.04 - -0.09 pair **(a)**, MHypo Bregma -0.09 - -0.14 pair **(b)**, MHypo Bregma -0.14 - -0.19 pair **(c)**, and MHypo Bregma -0.19 - -0.24 pair **(d)**. The first slice pair illustrating ground truth (GT) annotations. The values below each plot indicate the spot-to-spot matching ratio. **(e)** Bar plots representing the average spot-to-spot mapping ratio of each tool on MHypo dataset.

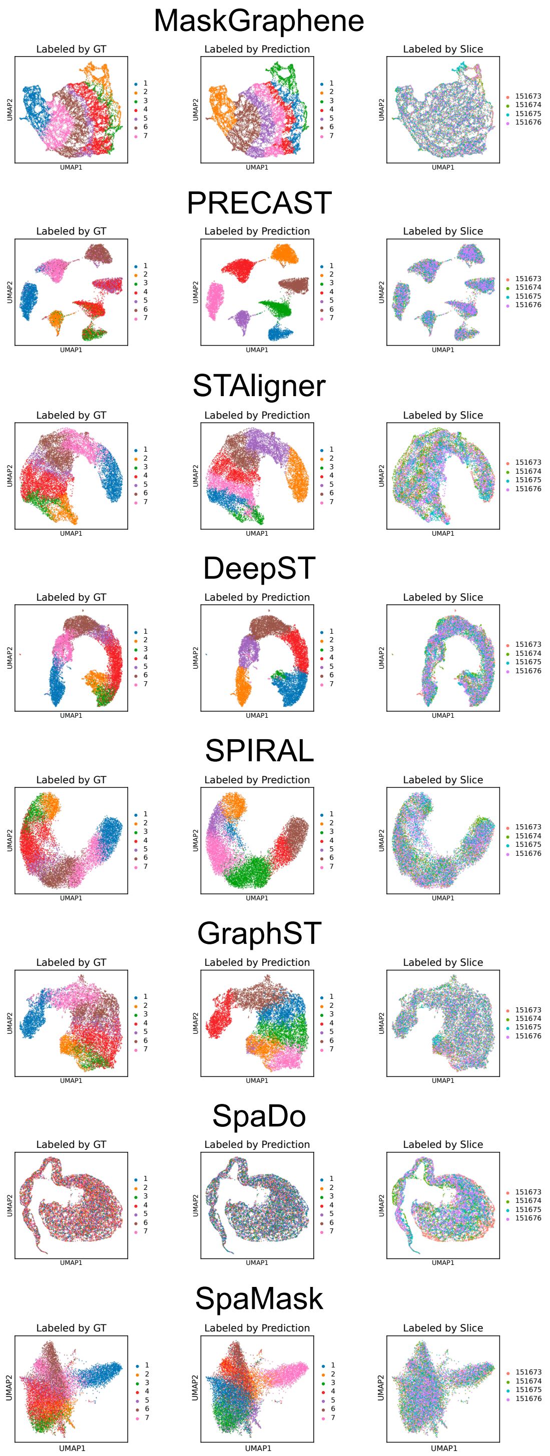

Fig. S8: **UMAP plots of low dimensional joint embedding on DLPFC four-slice integration (151673-151674-151675-151676).** UMAP visualizations of joint embeddings generated by different methods for the DLPFC four-slice integration. Spots are colored by ground truth (GT) labels, predicted domains, and slice identity. Each row corresponds to a method: MaskGraphene, PRECAST, STAligner, DeepST, SPIRAL, GraphST, SpaDo, and SpaMask.

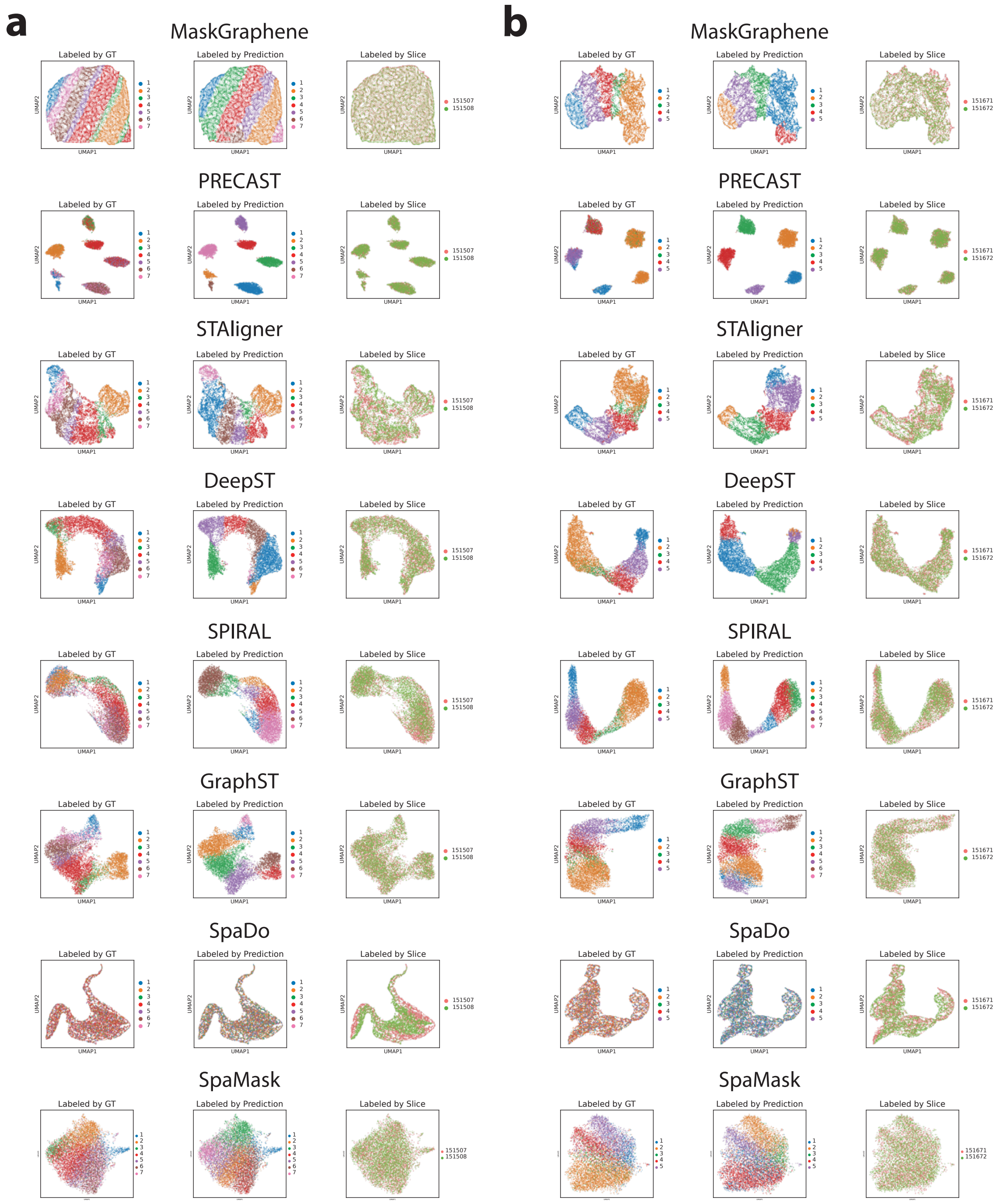

Fig. S9: **UMAP plots of low dimensional joint embedding on DLPFC pairwise integration.** **(a)** UMAP visualizations of joint embeddings generated by different methods for the DLPFC 151507-151508 pairwise integration. Spots are colored by ground truth (GT) labels, predicted domains, and slice identity. Each row corresponds to a method: MaskGraphene, PRECAST, STAligner, DeepST, SPIRAL, GraphST, SpaDo, and SpaMask. **(b)** UMAP visualizations of joint embeddings generated by different methods for the DLPFC 151671-151672 pairwise integration. Spots are colored by ground truth (GT) labels, predicted domains, and slice identity. Each row corresponds to a method: MaskGraphene, PRECAST, STAligner, DeepST, SPIRAL, GraphST, SpaDo, and SpaMask.

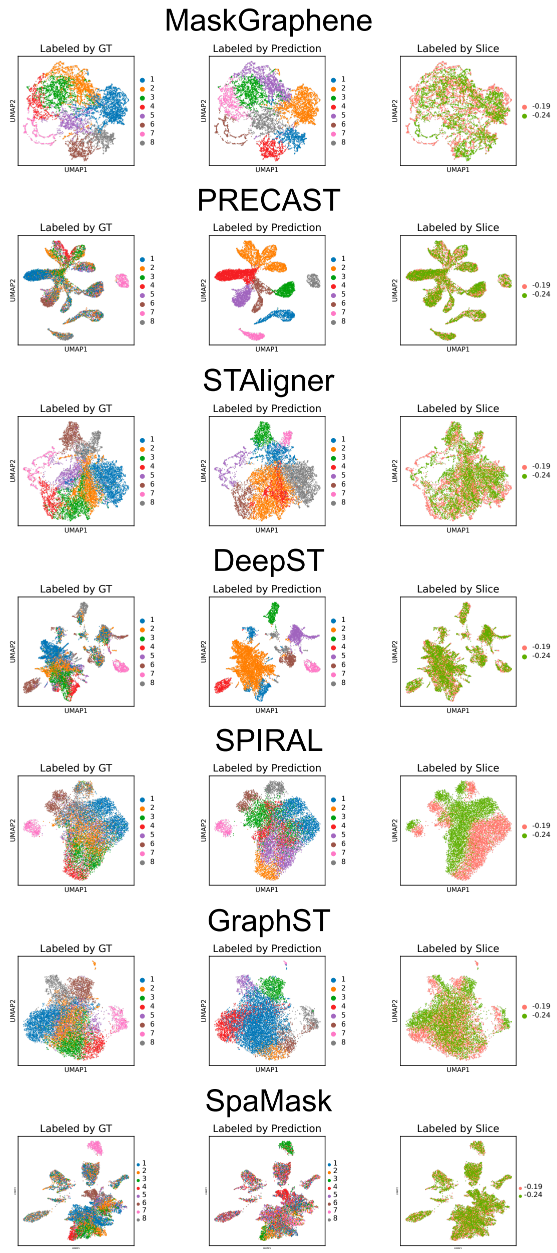

Fig. S10: **UMAP plots of low dimensional joint embedding on MHypo pairwise integration.** UMAP visualizations of joint embeddings generated by different methods for the MHypo -0.19 - -0.24 pairwise integration. Spots are colored by ground truth (GT) labels, predicted domains, and slice identity. Each row corresponds to a method: MaskGraphene, PRECAST, STAligner, DeepST, SPIRAL, GraphST, and SpaMask.

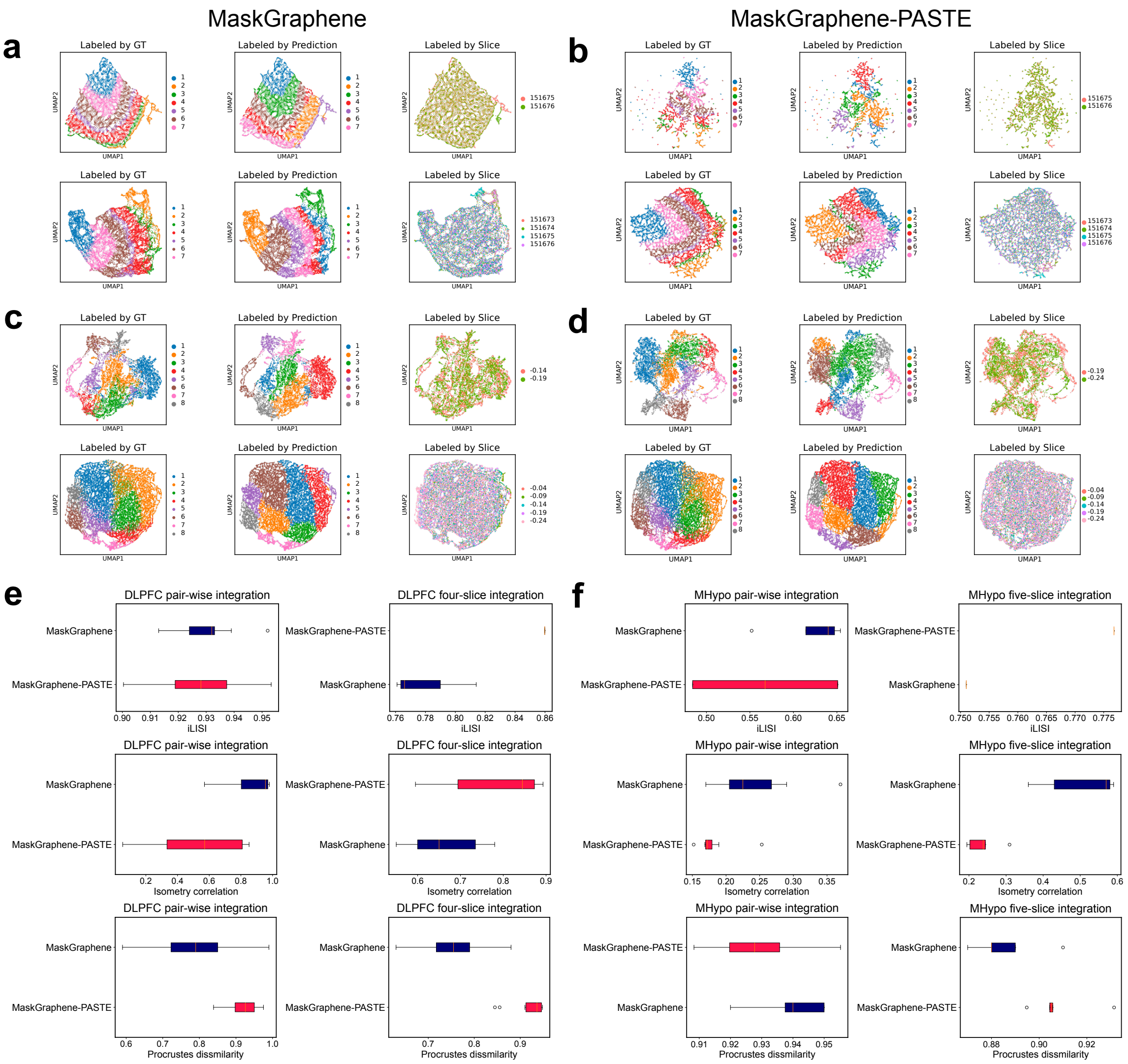

Fig. S11: **UMAP plots of low dimensional joint embedding and boxplots of evaluation metrics for the DLPFC dataset, comparing MaskGraphene and MaskGraphene-PASTE. (a-d)** UMAP visualizations of joint embeddings generated by two methods for the DLPFC and MHypo datasets under pairwise and multi-slice integration. Spots are colored by ground truth (GT) labels, predicted domains, and slice identity. **(e-f)** Boxplots illustrating performance comparison of iLISI, Isometry correlation, and Procrustes dissimilarity on DLPFC and MHypo datasets.

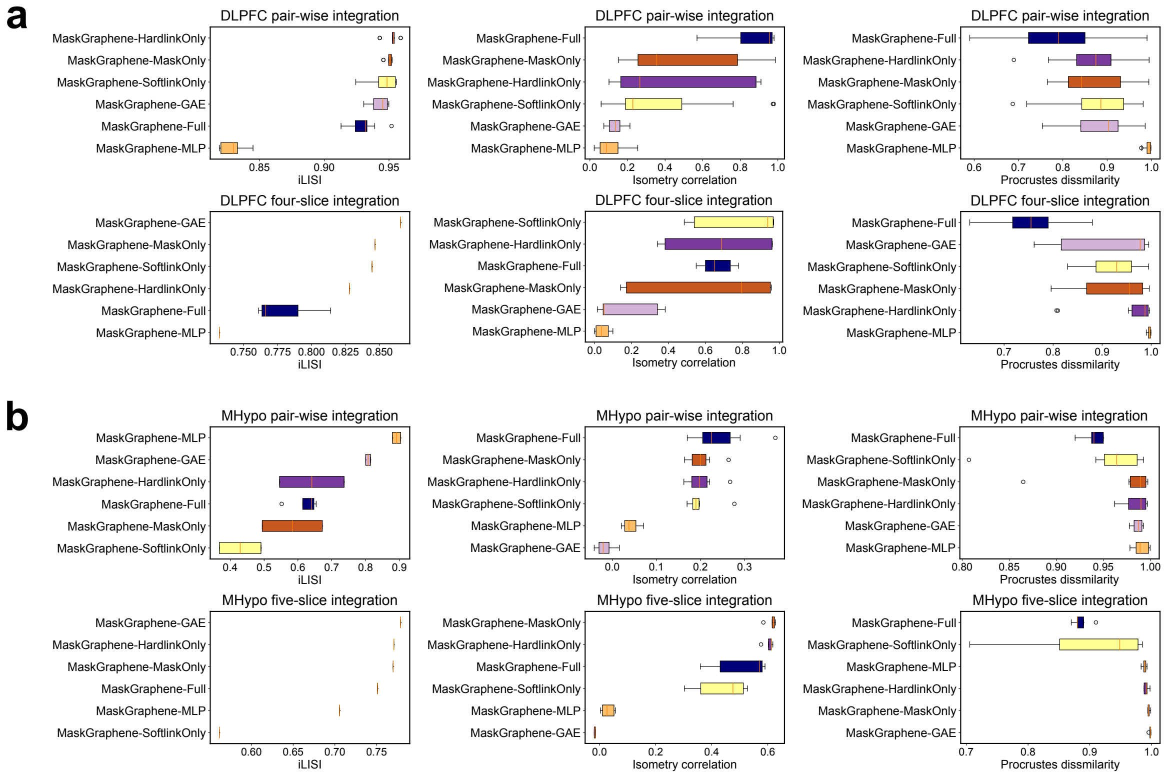

Fig. S12: **Boxplots of evaluation metrics from ablation studies on the DLPFC and MHypo datasets. (a-b)** Boxplots comparing iLISI, Isometry correlation, and Procrustes dissimilarity under pairwise and multi-slice integration scenarios on the DLPFC and MHypo datasets for different model variant.

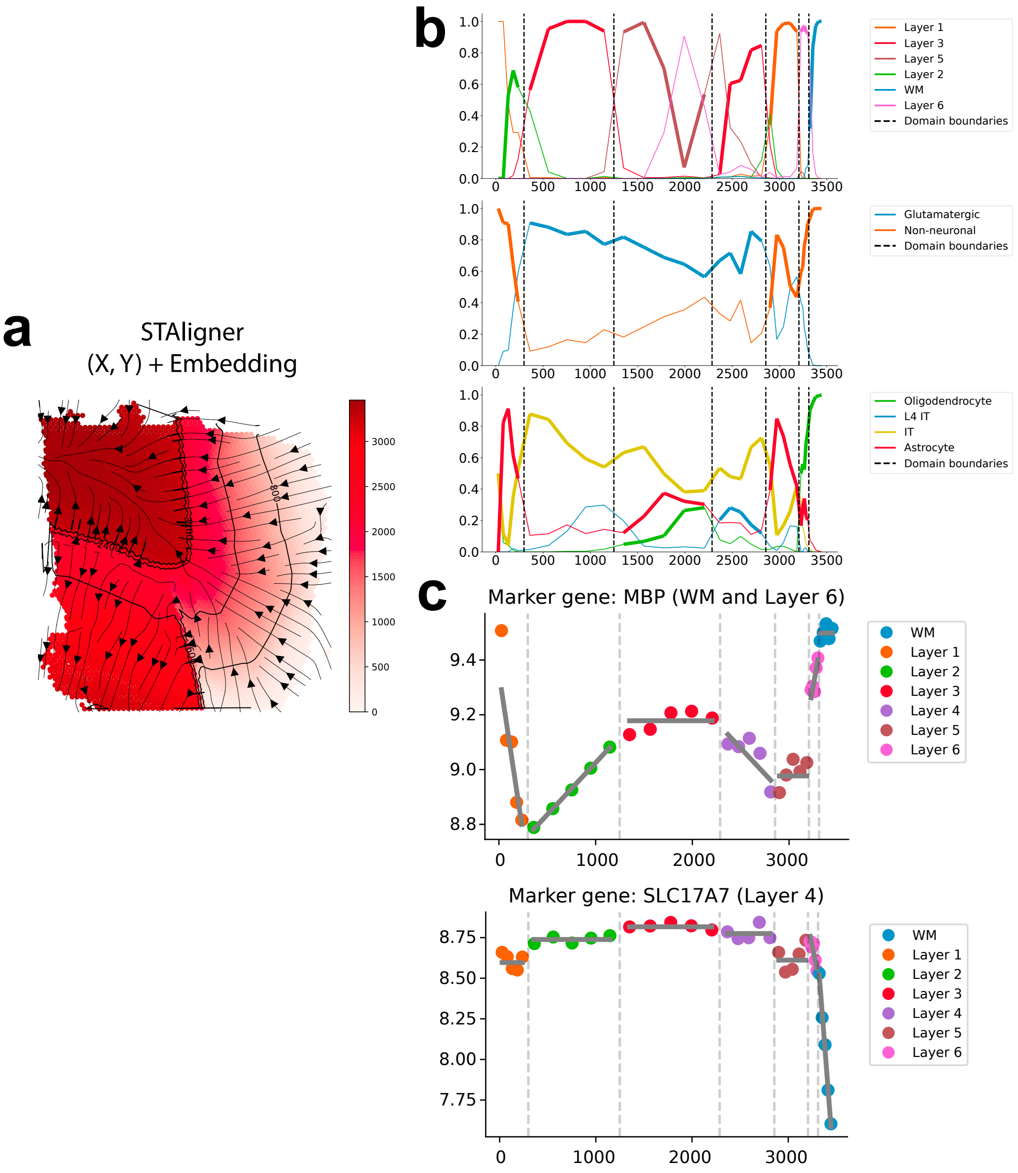

Fig. S13: **Topography analysis based on STAligner embeddings after DLPFC 151673-151674 pairwise integration.** **(a)** Topographical maps generated using GASTON with original X,Y coordinates combined with joint embeddings of STAligner. **(b)** Plots showing the proportions of cell types as a function of the isodepth, using three different types of annotations: layer-specific cell types (top panel), neuronal types (middle panel), and cell types (bottom panel). **(c)** Plots showing the marker gene (MBP and SLC17A7) expression versus the isodepth.

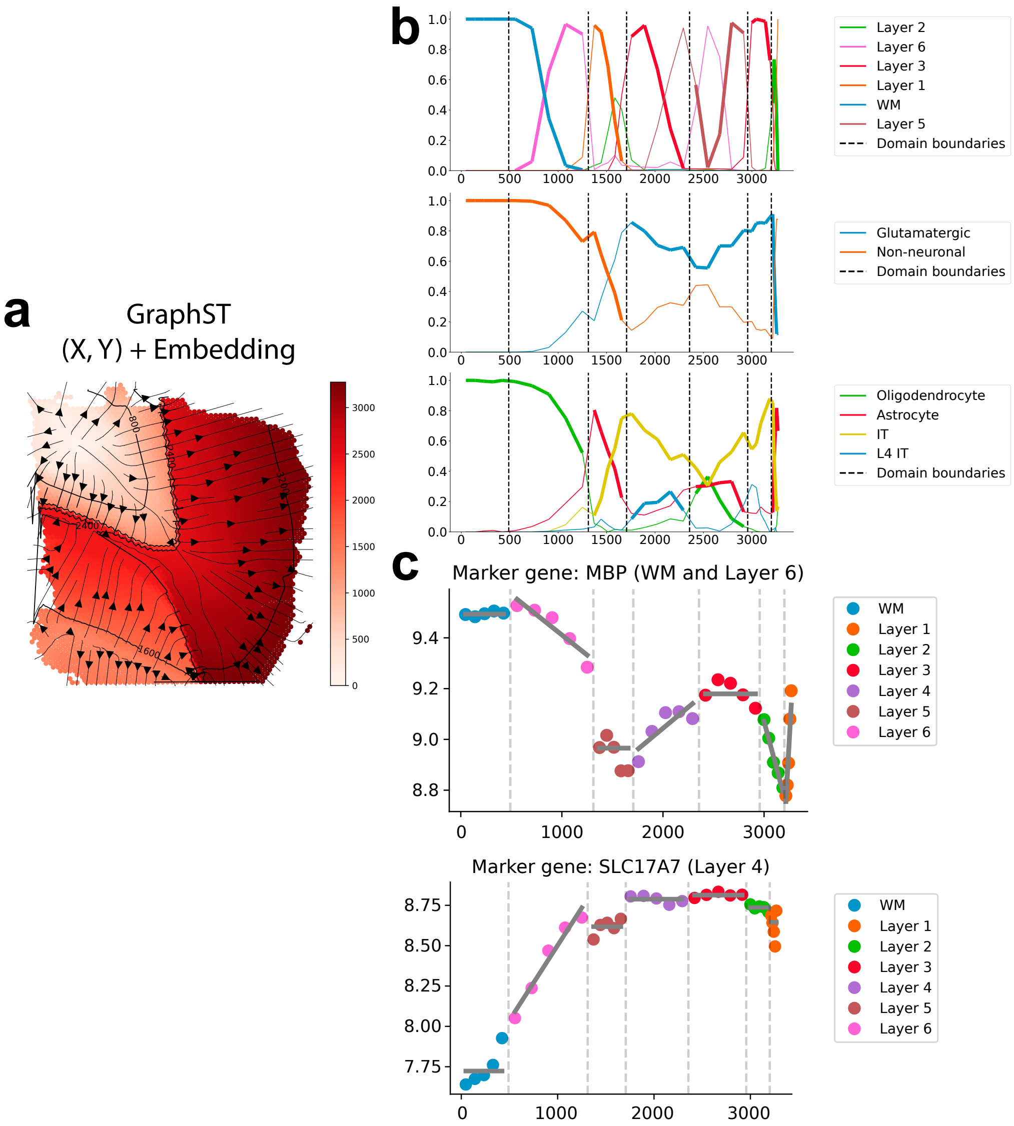

Fig. S14: **Topography analysis based on GraphST embeddings after DLPFC 151673-151674 pairwise integration.** **(a)** Topographical maps generated using GASTON with original X,Y coordinates combined with joint embeddings of GraphST. **(b)** Plots showing the proportions of cell types as a function of the isodepth, using three different types of annotations: layer-specific cell types (top panel), neuronal types (middle panel), and cell types (bottom panel). **(c)** Plots showing the marker gene (MBP and SLC17A7) expression versus the isodepth.

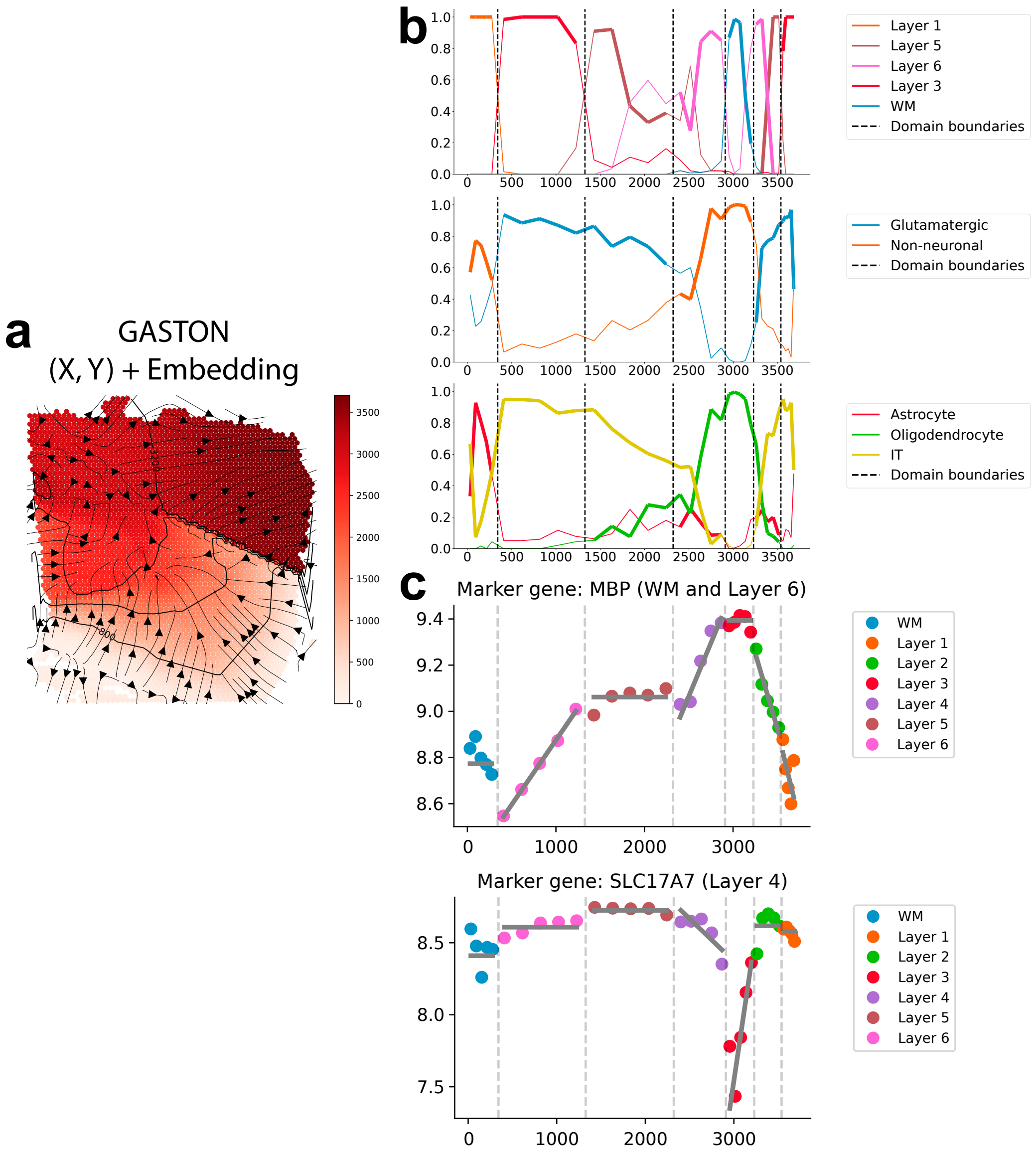

Fig. S15: **Topography analysis based on PCA-derived embeddings from individual DLPFC slice 151673.** **(a)** Topographical maps generated using GASTON with original X,Y coordinates combined with PCA-derived embeddings from DLPFC slice 151673 (GASTON's default setting). **(b)** Plots showing the proportions of cell types as a function of the isodepth, using three different types of annotations: layer-specific cell types (top panel), neuronal types (middle panel), and cell types (bottom panel). **(c)** Plots showing the marker gene (MBP and SLC17A7) expression versus the isodepth.

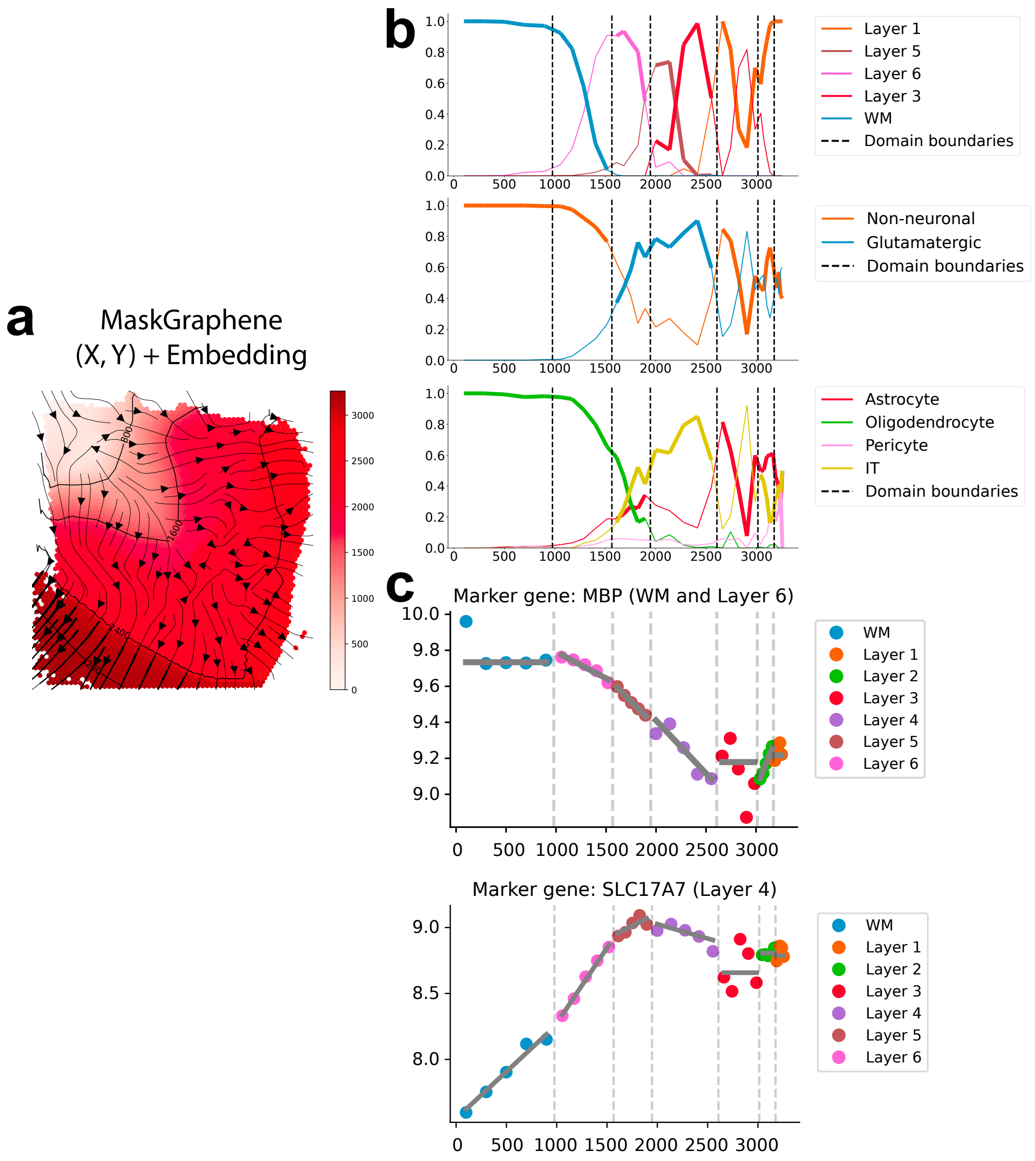

Fig. S16: **Topography analysis based on MaskGraphene embeddings after DLPFC four-slice integration (151673-151674-151675-151676). (a)** Topographical maps generated using GASTON with original X,Y coordinates combined with joint embeddings of MaskGraphene. **(b)** Plots showing the proportions of cell types as a function of the isodepth, using three different types of annotations: layer-specific cell types (top panel), neuronal types (middle panel), and cell types (bottom panel). **(c)** Plots showing the marker gene (MBP and SLC17A7) expression versus the isodepth.

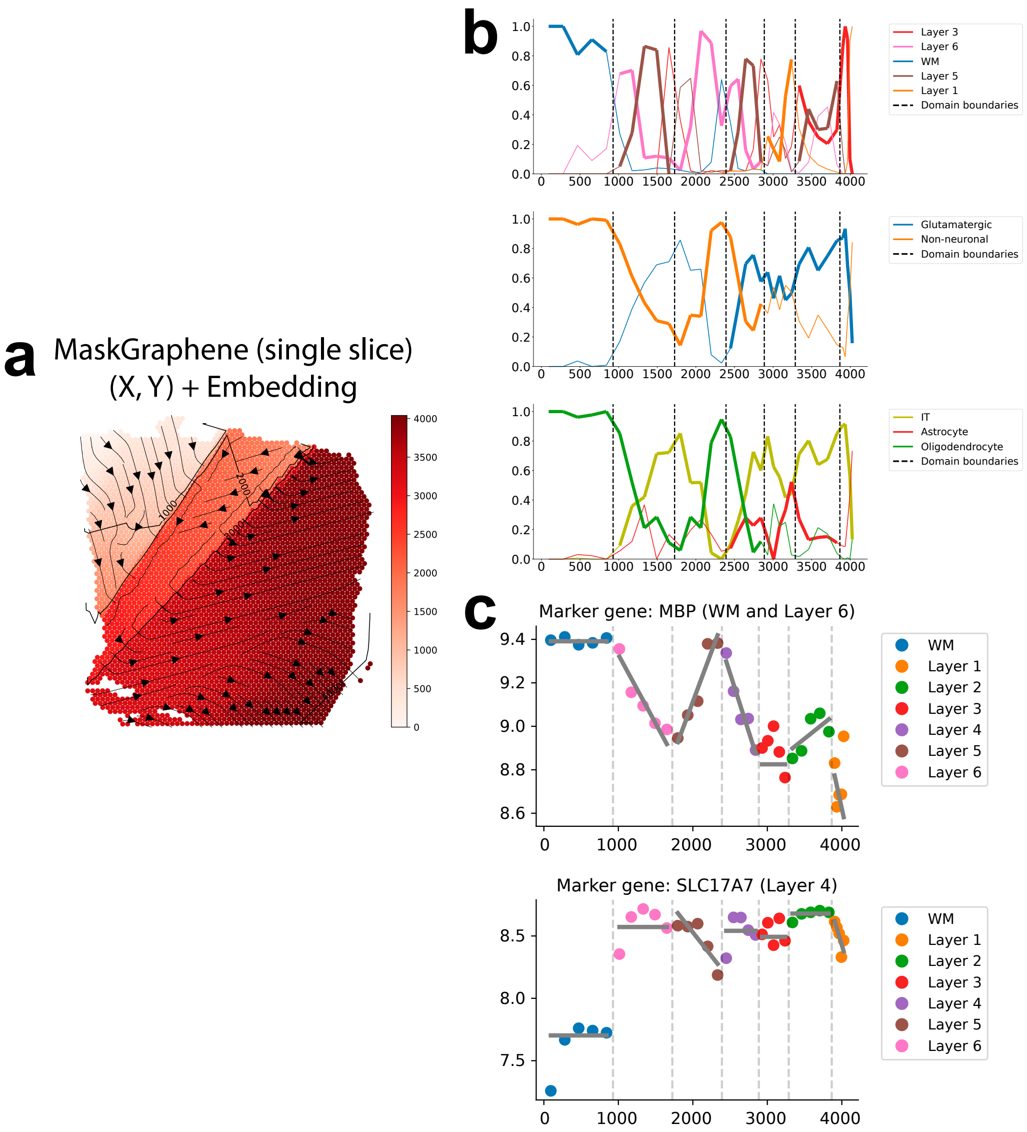

Fig. S17: **Topography analysis based on MaskGraphene embeddings of DLPFC 151673.** **(a)** Topographical maps generated using GASTON with original X,Y coordinates combined with single slice embeddings of MaskGraphene. **(b)** Plots showing the proportions of cell types as a function of the isodepth, using three different types of annotations: layer-specific cell types (top panel), neuronal types (middle panel), and cell types (bottom panel). **(c)** Plots showing the marker gene (MBP and SLC17A7) expression versus the isodepth.

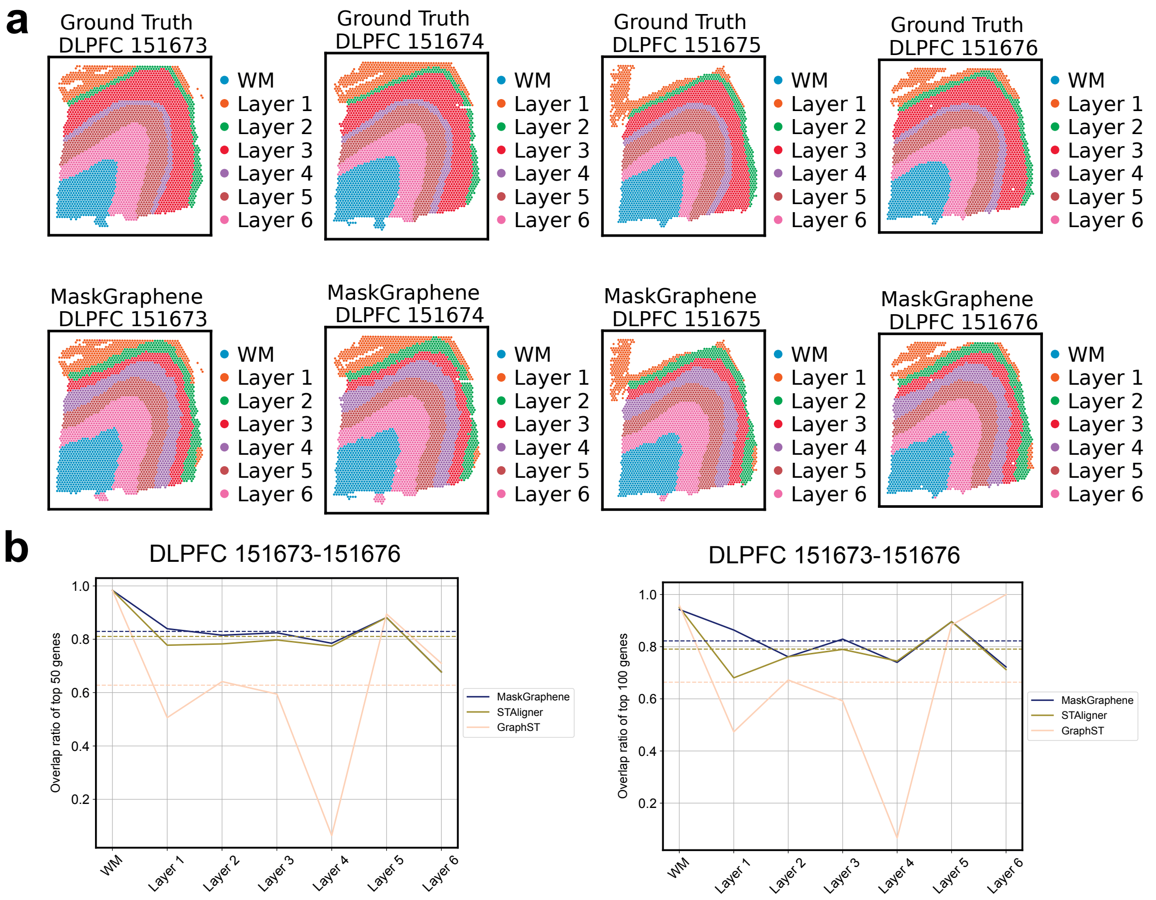

Fig. S18: **Biomarker and topography analysis after brain slice integration.** **(a)** Spatial visualizations of joint domain identification for four DLPFC slices (151673, 151674, 151675, and 151676) based on ground truth (top panels) and MaskGraphene predictions after integration (bottom panels). Colored annotations indicate white matter (WM) and six cortical layers (1–6) across two conditions. **(b)** Biomarker overlap ratio curves across WM and cortical layers for three different tools, comparing the top N biomarkers identified for each integrated layer after four-slice integration with the N ground truth marker genes. Ground truth markers are defined as the union of the top 50 (left panel) or top 100 (right panel) layer marker genes across four slices.

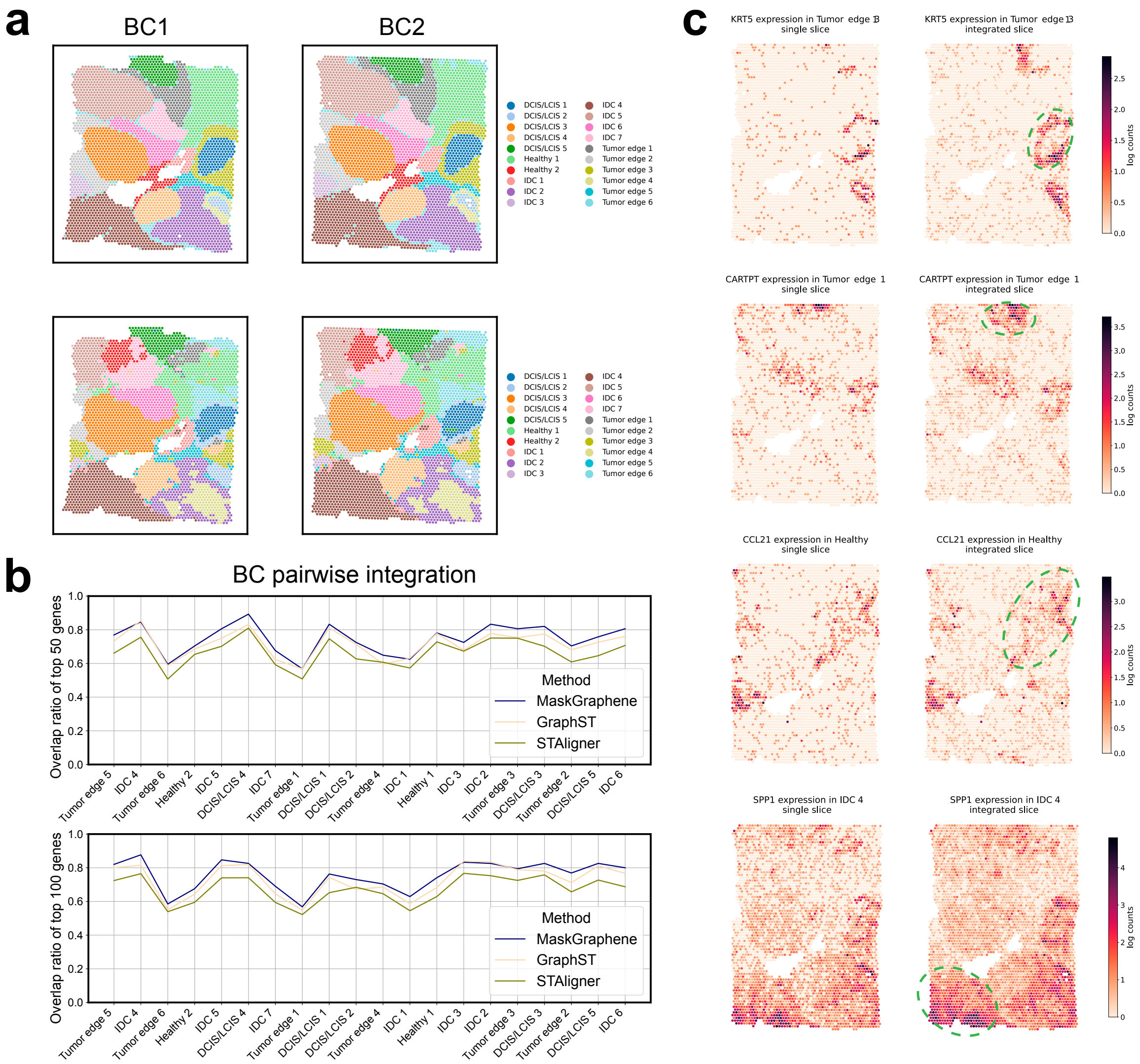

Fig. S19: **Biomarker analysis after breast cancer pairwise integration.** **(a)** Spatial visualizations of joint domain identification for two breast cancer slices (BC1 and BC2) based on ground truth (top panels) and MaskGraphene predictions after integration (bottom panels). Colored annotations indicate DCIS/LCIS, healthy tissue, invasive ductal carcinoma (IDC), and low-grade tumor margins across two conditions. **(b)** Biomarker overlap ratio curves across all 20 regions for three different tools, comparing the top N biomarkers identified for each integrated layer after four-slice integration with the N ground truth marker genes. Ground truth markers are defined as the union of the top 50 (top panel) or top 100 (bottom panel) domain marker genes across four slices. **(c)** Spatial expression patterns and domain-specific distributions of KRT5, CARTPT, CCL21, and SPP1 in single-slice (left) and integrated-slice (right) analyses. Green lines highlight the boundaries of the specific domain associated with each gene.

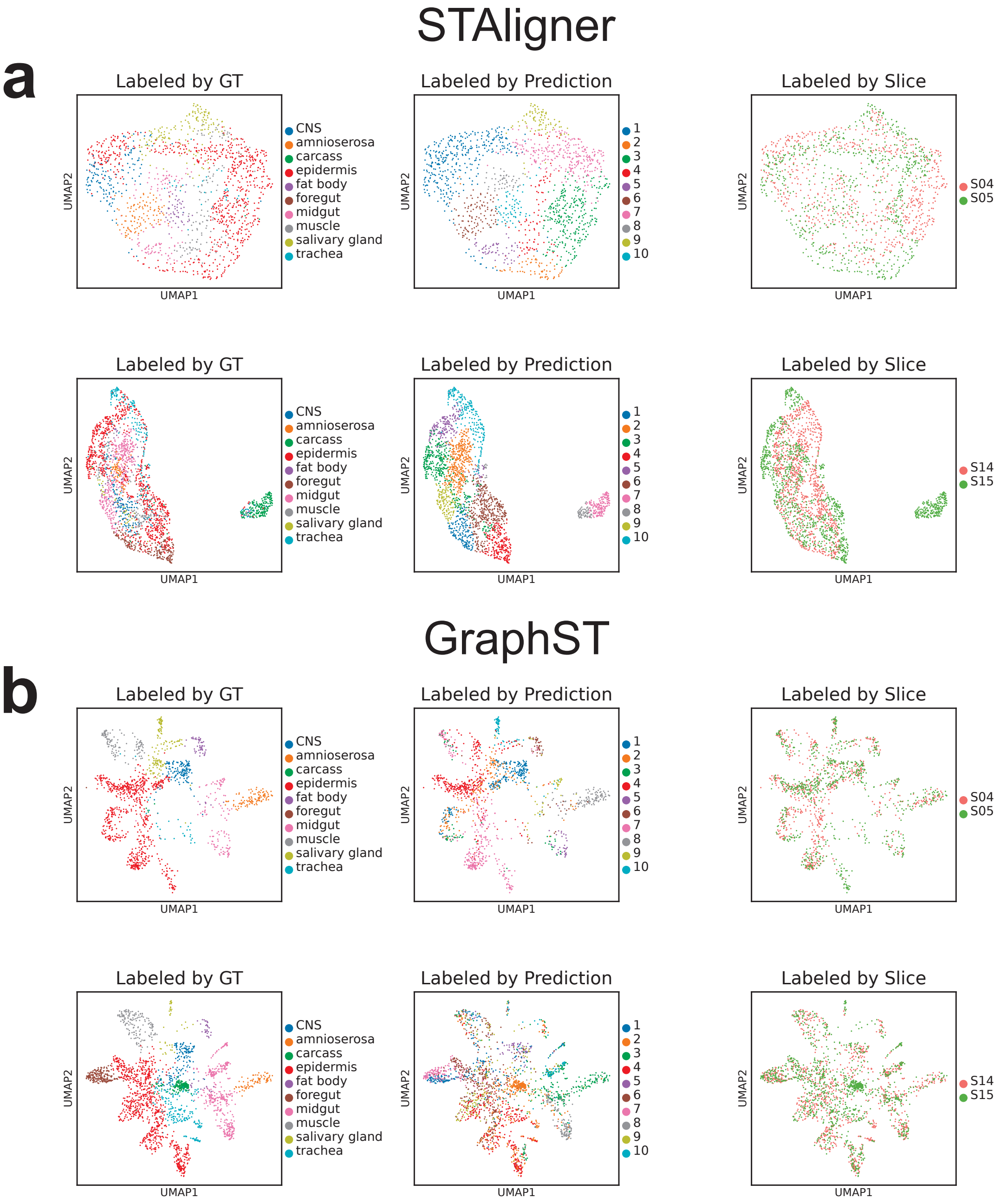

Fig. S20: **UMAP visualizations of low-dimensional joint embeddings generated by STAligner and GraphST on the Drosophila dataset. (a-b)** UMAP plots showing STAligner and GraphST joint embeddings for Drosophila pairwise integrations of slices S04-S05 and S14-S15.

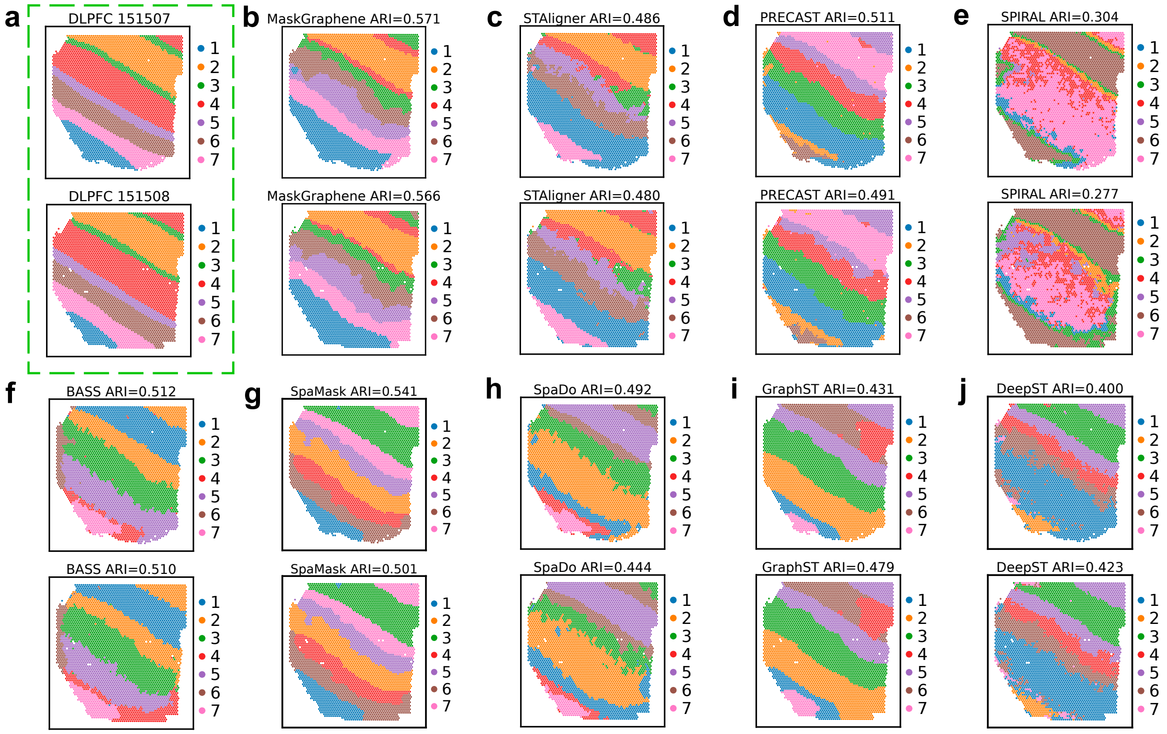

Fig. S21: **Spatial visualization of joint domain identification after DLPFC 151507-151508 pairwise integration.** **(a)** Spatial domain identification visualization for two DLPFC slices (151507 and 151508) by ground truth. **(b-i)** Spatial domain identification visualization with ARI value for two DLPFC slices (151507 and 151508) after pairwise integration using eight integration methods, including MaskGraphene (b), STAligner (c), PRECAST (d), SPIRAL (e), BASS (f), SpaMask (g), SpaDo (h), GraphST (i), and DeepST (j).

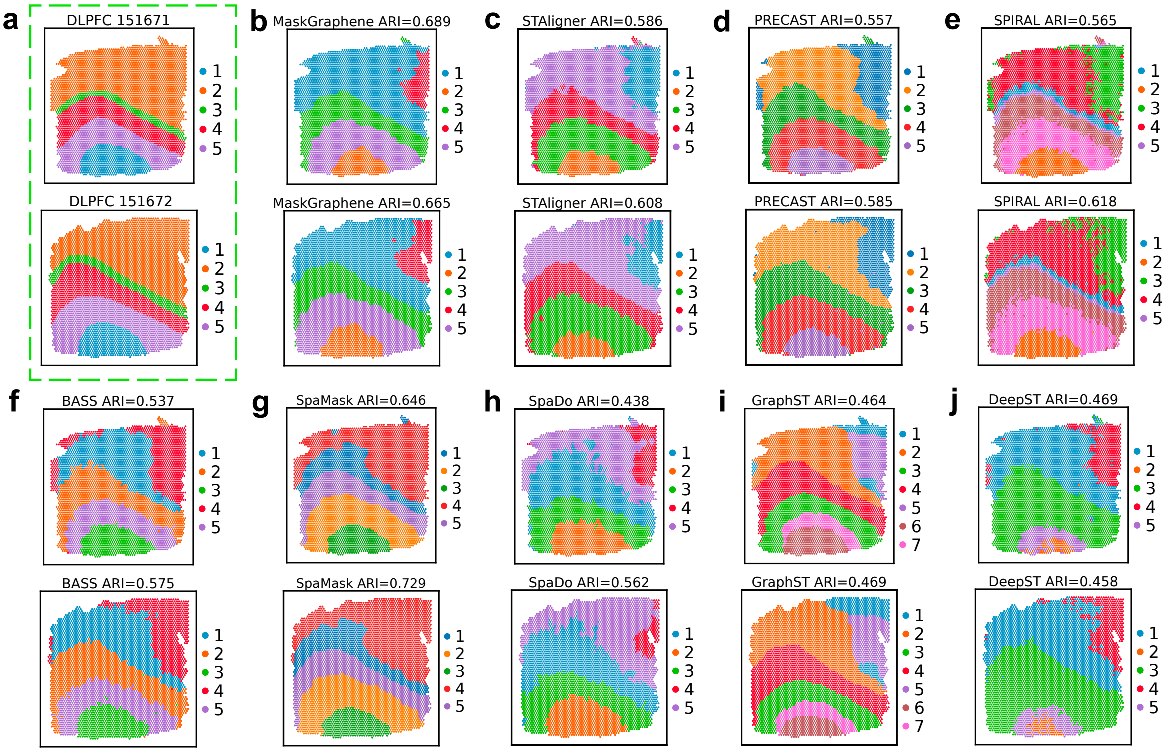

Fig. S22: **Spatial visualization of joint domain identification after DLPFC 151671-151672 pairwise integration.** **(a)** Spatial domain identification visualization for two DLPFC slices (151671 and 151672) by ground truth. **(b-i)** Spatial domain identification visualization with ARI value for two DLPFC slices (151671 and 151672) after pairwise integration using eight integration methods, including ground truth (a), MaskGraphene (b), STAligner (c), PRECAST (d), SPIRAL (e), BASS (f), SpaMask (g), SpaDo (h), GraphST (i), and DeepST (j).

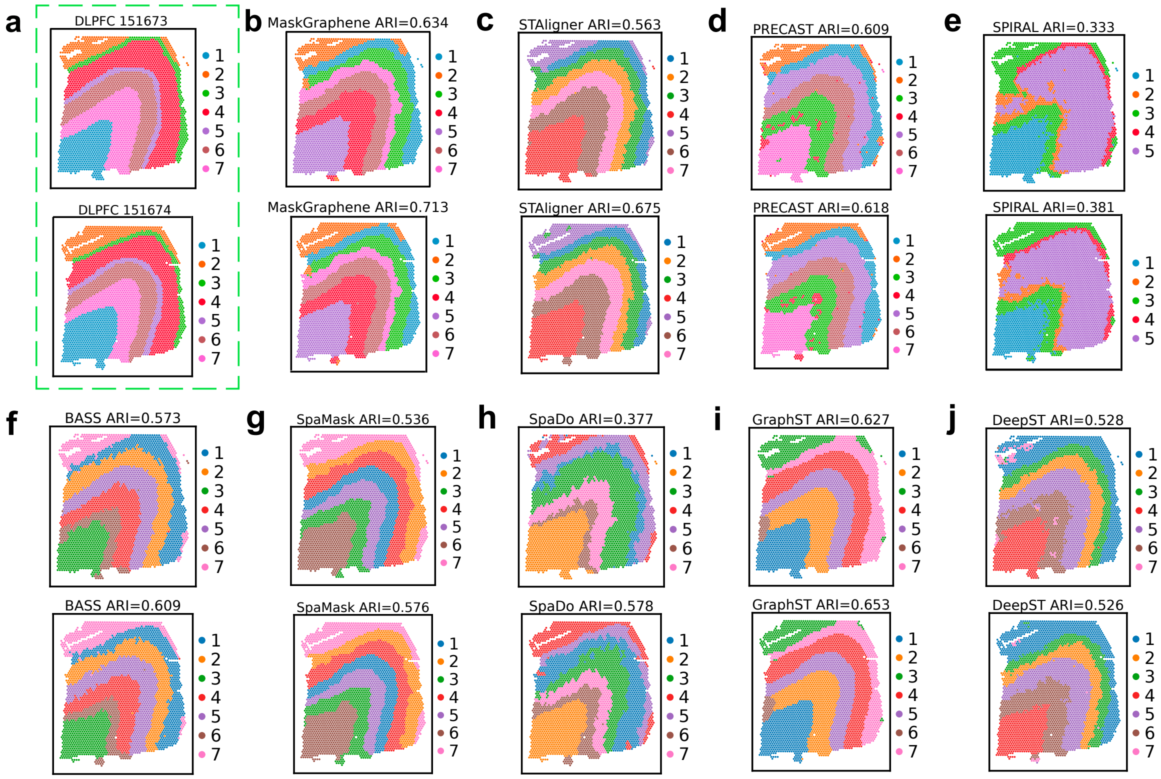

Fig. S23: **Spatial visualization of joint domain identification after DLPFC 151673-151674 pairwise integration.** **(a)** Spatial domain identification visualization for two DLPFC slices (151673 and 151674) by ground truth. **(b-i)** Spatial domain identification visualization with ARI value for two DLPFC slices (151673 and 151674) after pairwise integration using eight integration methods, including ground truth (a), MaskGraphene (b), STAligner (c), PRECAST (d), SPIRAL (e), BASS (f), SpaMask (g), SpaDo (h), GraphST (i), and DeepST (j).

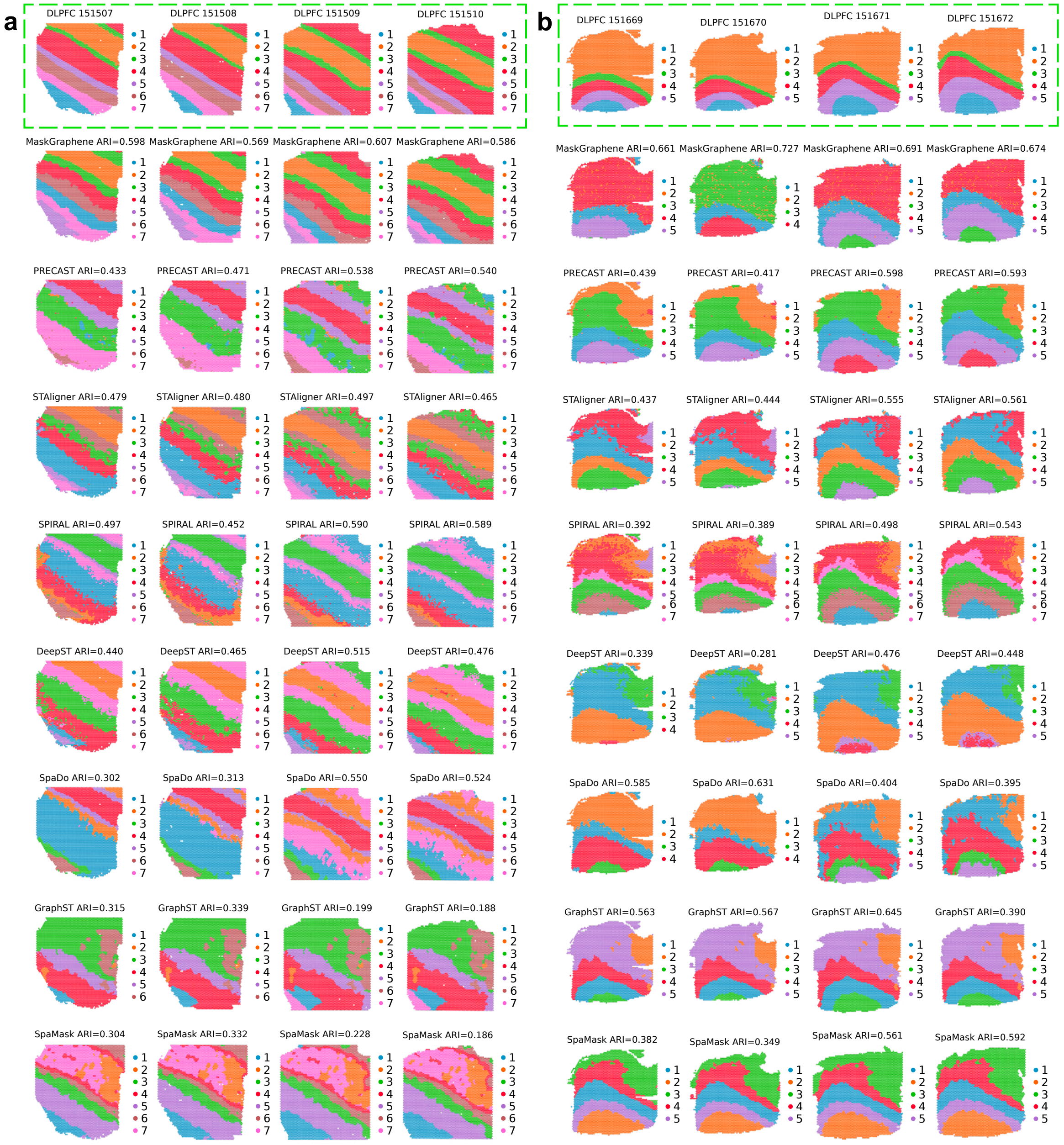

Fig. S24: **Spatial visualization of joint domain identification after four-slice integration (151507-151508-151509-151510).** **(a)** Spatial domain identification visualization with ARI value for four DLPFC slices (151507, 151508, 151509 and 151510) after four-slice integration using seven integration methods, including MaskGraphene, PRECAST, STAligner, SPIRAL, DeepST, SpaDo, GraphST, and SpaMask. Each row corresponds to a different method and ground truth. **(b)** Spatial domain identification visualization for four DLPFC slices (151669, 151670, 151671 and 151672) after integration using seven integration methods, including MaskGraphene, PRECAST, STAligner, SPIRAL, DeepST, SpaDo, GraphST, and SpaMask. Each row corresponds to a different method and ground truth.

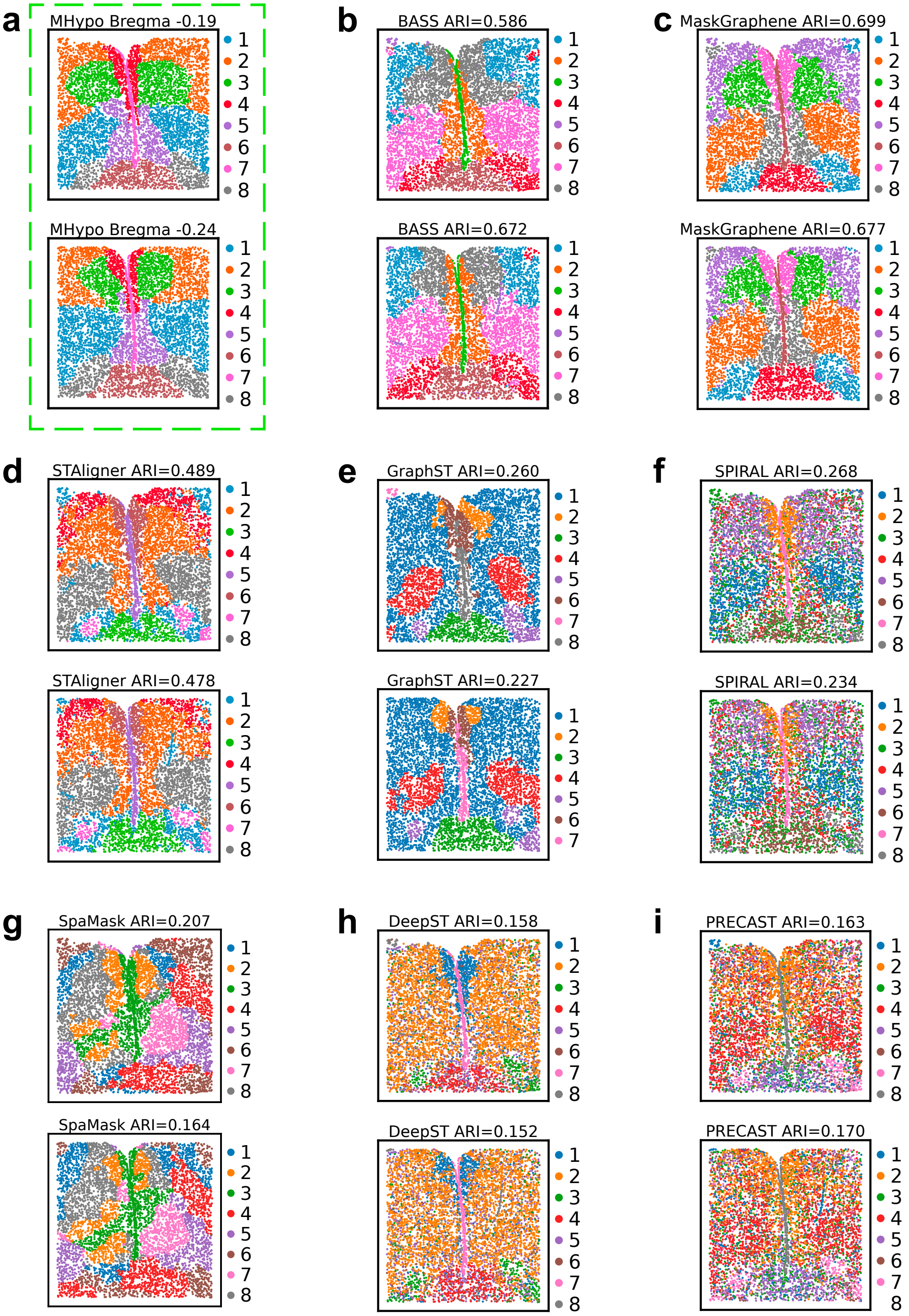

Fig. S25: **Spatial visualization of joint domain identification after MHypo Bregma -0.19 - -0.24 pairwise integration.** **(a)** Spatial domain identification visualization for two MHypo slices (Bregma -0.19 and Bregma -0.24) by ground truth. **(b-h)** Spatial domain identification visualization with ARI value for two MHypo slices (Bregma -0.19 and Bregma -0.24) after pairwise integration using seven integration methods, including BASS (b), MaskGraphene (c), STAligner (d), GraphST (e), SPIRAL (f), SpaMask (g), DeepST (h), and PRECAST (i).

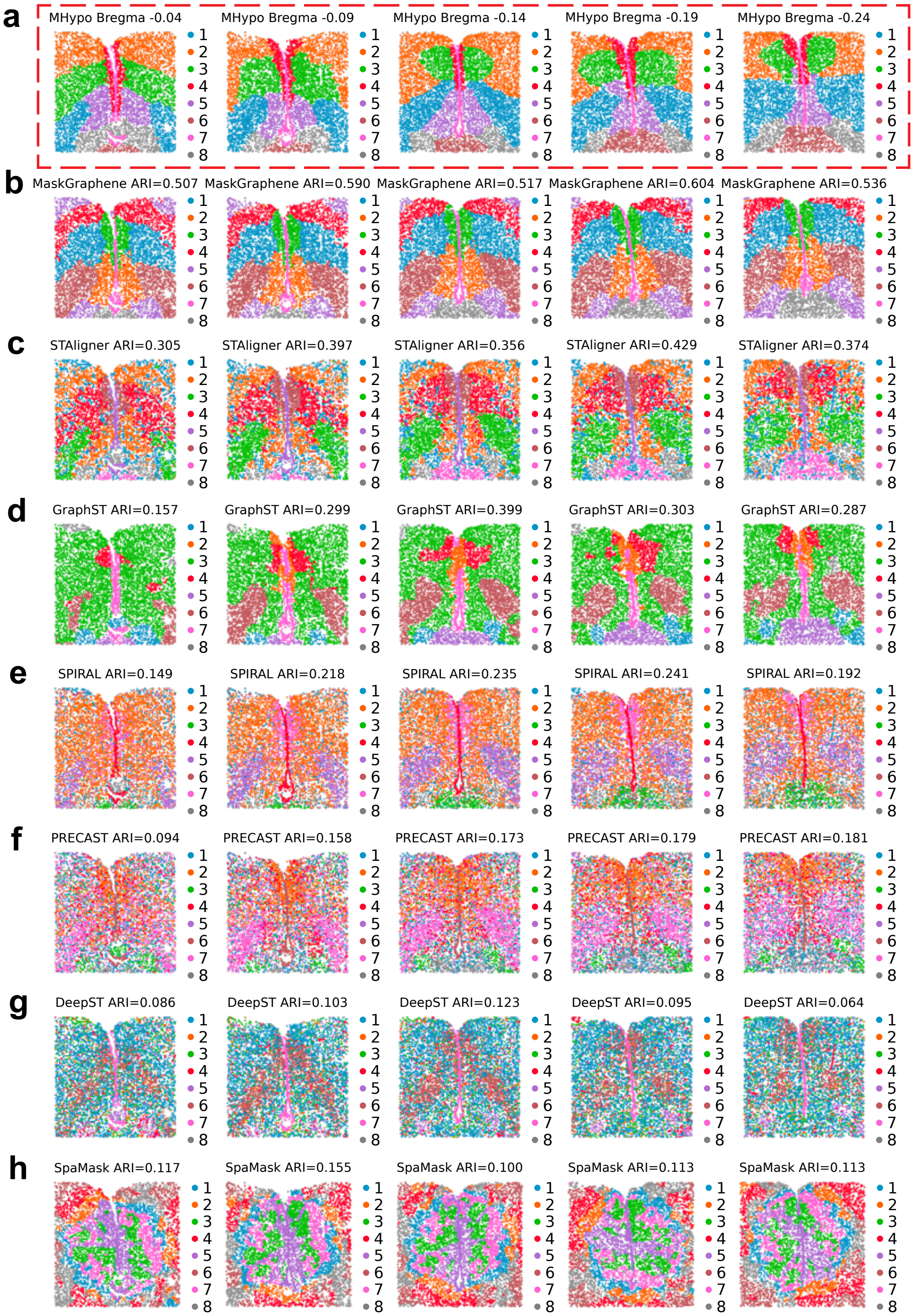

Fig. S26: **Spatial visualization of joint domain identification after MHypo five-slice integration.** **(a-g)** Spatial domain identification visualization for five MHypo slices (Bregma -0.04, Bregma -0.09, Bregma -0.14, Bregma -0.19 and Bregma -0.24) after five-slice integration using seven integration methods, including MaskGraphene, STAligner, GraphST, SPIRAL, PRECAST, DeepST, and SpaMask. Each row corresponds to a different method and ground truth.

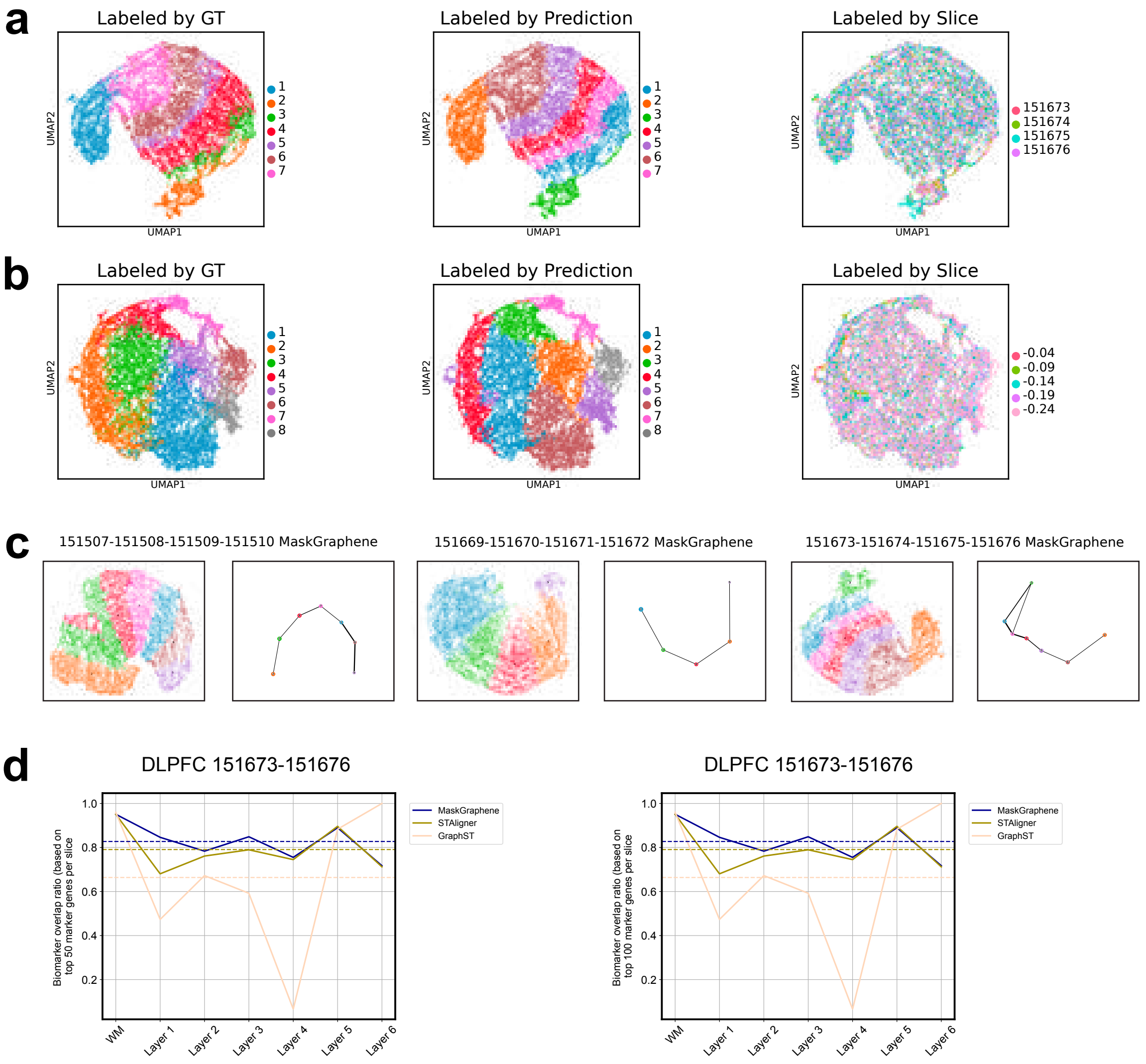

Fig. S27: **UMAP, PAGA, and biomarker analysis of MaskGraphene (coordinate transformation) after multi-slice integration.** **(a)** UMAP visualizations of joint embeddings generated by MaskGraphene (coordinate transformation) for the DLPFC four-slice integration (151673-151674-151675-151676). Spots are colored by ground truth (GT) labels, predicted domains, and slice identity. This subfigure corresponds to Figure 4b using MaskGraphene (coordinate replacement). **(b)** UMAP visualizations of joint embeddings generated by MaskGraphene (coordinate transformation) for the MHypo five-slice integration (Brega -0.04 - -0.09 - -0.14 - -0.19 - -0.24). Spots are colored by ground truth (GT) labels, predicted domains, and slice identity. This subfigure corresponds to Figure 5b using MaskGraphene (coordinate replacement). **(c)** Each two panels shows UMAP visualizations paired with PAGA graphs by MaskGraphene (coordinate transformation), illustrating spatial trajectory results for three distinct DLPFC four-slice integration: (151507-151508-151509-151510), (151669-151670-151671-151672), and (151673-151674-151675-151676). Spots are colored according to predicted domains. This subfigure corresponds to Figure 6 using MaskGraphene (coordinate replacement). **(d)** Biomarker overlap ratio curves across WM and cortical layers for MaskGraphene (coordinate transformation), comparing the top N biomarkers identified for each integrated layer after four-slice integration with the N ground truth marker genes. Ground truth markers are defined as the union of the top 50 (left panel) or top 100 (right panel) layer marker genes across four slices. This subfigure corresponds to Figure 7b using MaskGraphene (coordinate replacement).

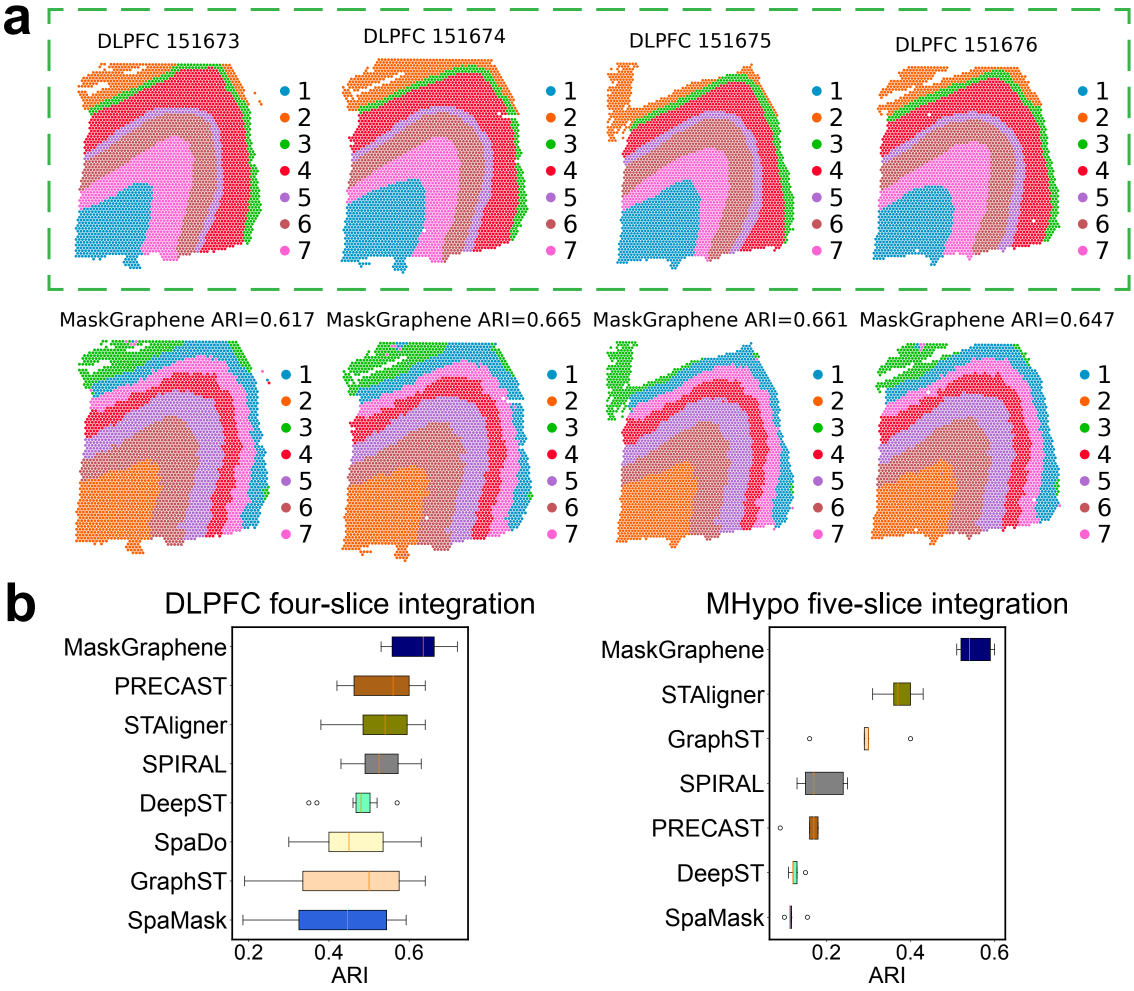

Fig. S28: **Spatial visualization of joint domain identification and ARI boxplots by MaskGraphene (coordinate transformation) after multi-slice integration.** **(a)** Visualization of spatial domain identification for four DLPFC slices (151673, 151674, 151675, and 151676), showing ground truth (top panels enclosed in a green dashed box) and results after integration using MaskGraphene (coordinate transformation) (bottom panels) **(b)** Box plots showing ARI scores for all DLPFC four-slice integration and all MHypo five-slice integration by MaskGraphene (coordinate transformation) and all other integration methods. This Figure corresponds to Figure 9 using MaskGraphene (coordinate replacement).

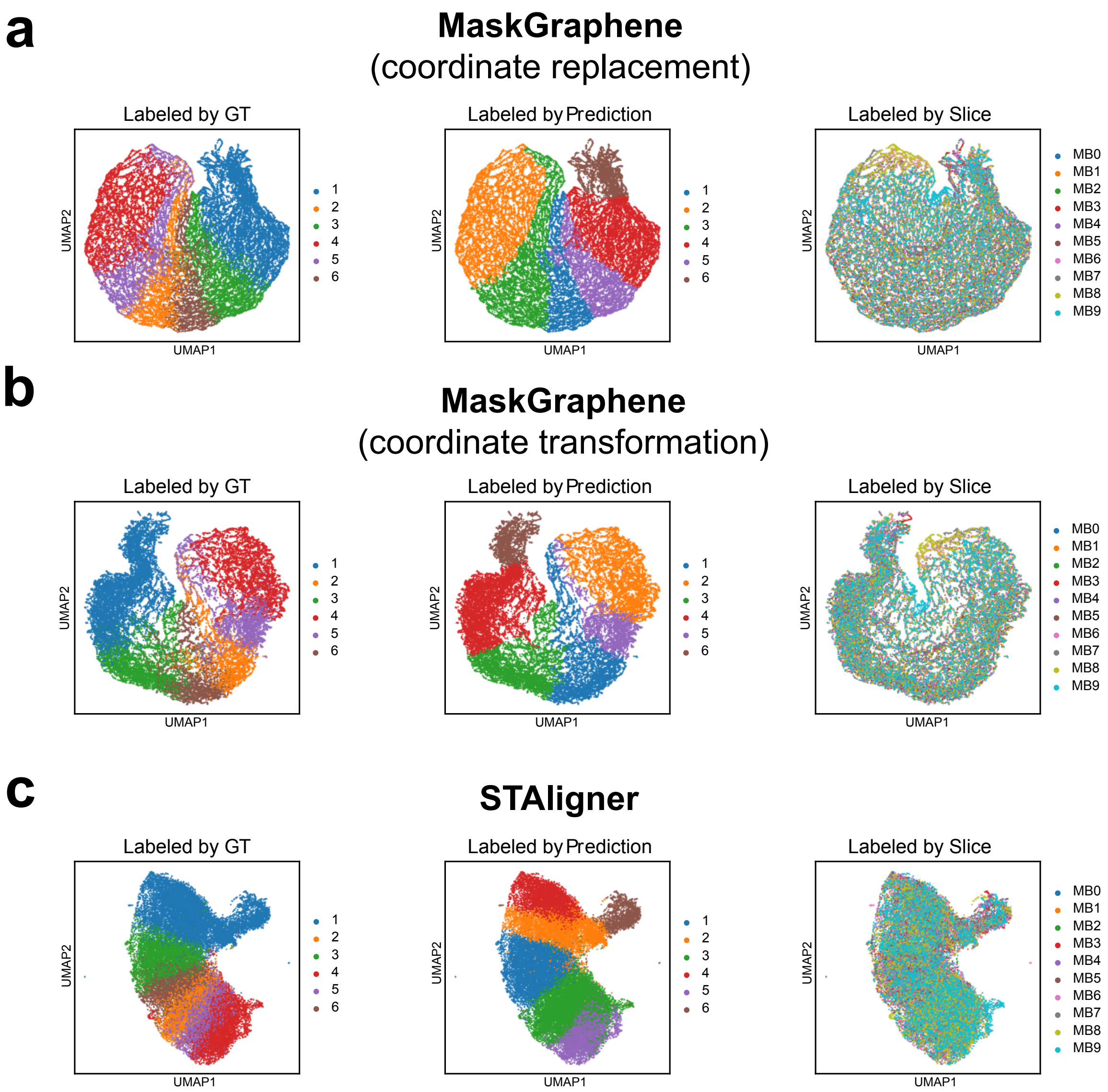

Fig. S29: **UMAP plots of low dimensional joint embedding on the MB dataset.** **(a)** UMAP visualizations of joint embeddings generated by MaskGraphene (coordinate replacement). Spots are colored by ground truth (GT) labels, predicted domains, and slice identity. **(b)** UMAP visualizations of joint embeddings generated by MaskGraphene (coordinate transformation). Spots are colored by ground truth (GT) labels, predicted domains, and slice identity. **(c)** UMAP visualizations of joint embeddings generated by STAligner. Spots are colored by ground truth (GT) labels, predicted domains, and slice identity.

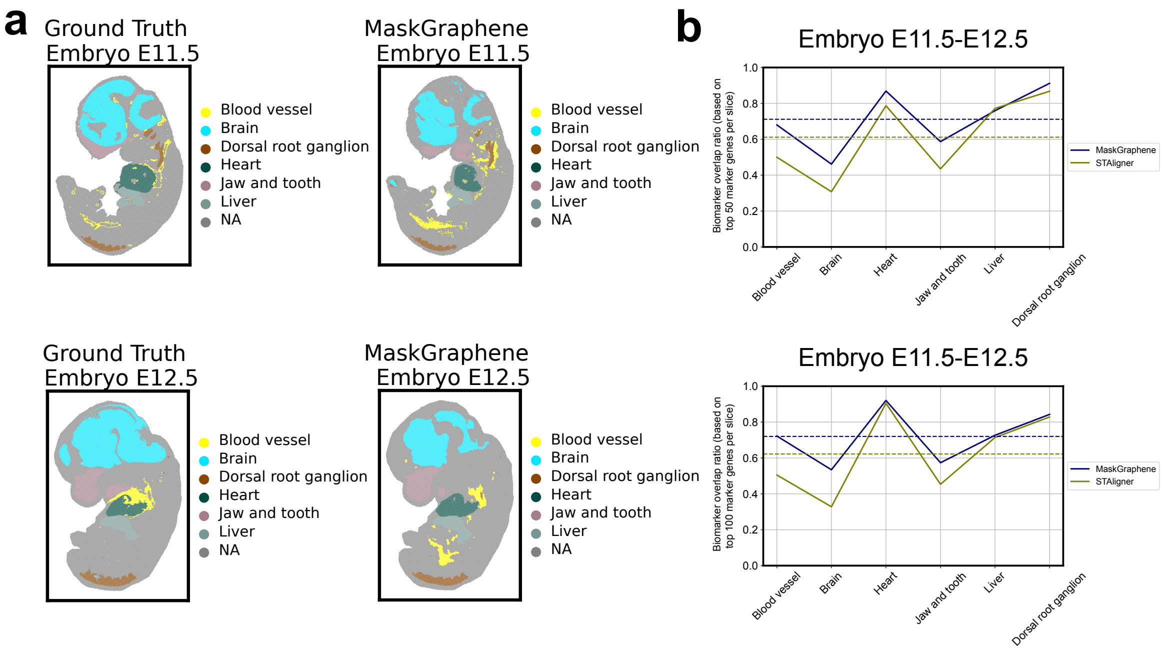

Fig. S30: **Spatial visualizations and biomarker analysis on the mouse embryo dataset. (a)** Spatial visualizations of joint domain identification for two embryo slices based on ground truth and MaskGraphene predictions after integration. Matched colored annotations indicate several shared key tissue structures across the two conditions. **(b)** Biomarker overlap ratio curves across shared tissue structures for MaskGraphene and STAligner, comparing the top N biomarkers identified for each integrated tissue structure after integration with the N ground truth marker genes. Ground truth markers are defined as the union of the top 50 (left panel) or top 100 (right panel) tissue marker genes across the two slices.

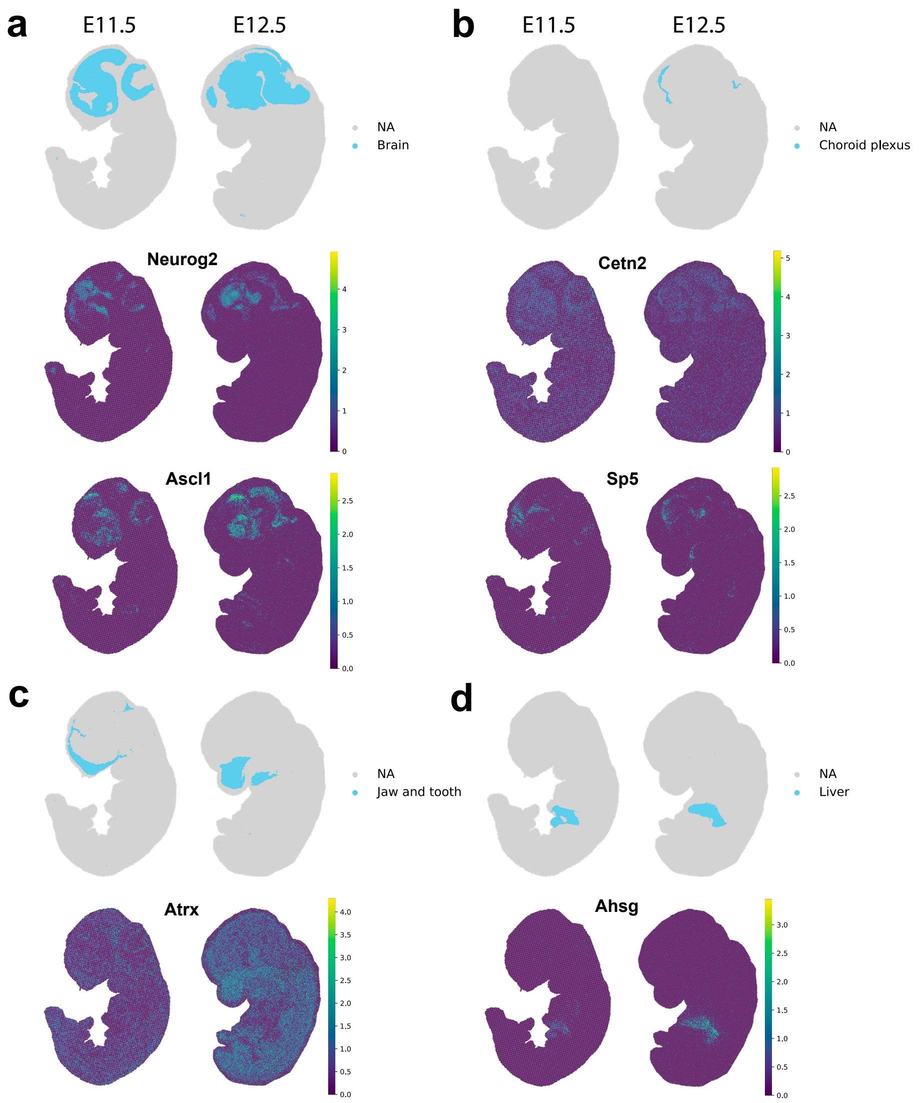

Fig. S31: **Visualization of marker genes from the integrated analysis.** Spatial mapping of representative tissue predictions (ground truth) alongside expression patterns of four example top marker genes (Neurog2, Ascl1, Cetn2, Sp5, Atrx, and Ahsg) from the integrated analysis, illustrating the correspondence between predicted domains and gene expression enrichment in individual developmental slices (E11.5 and E12.5).

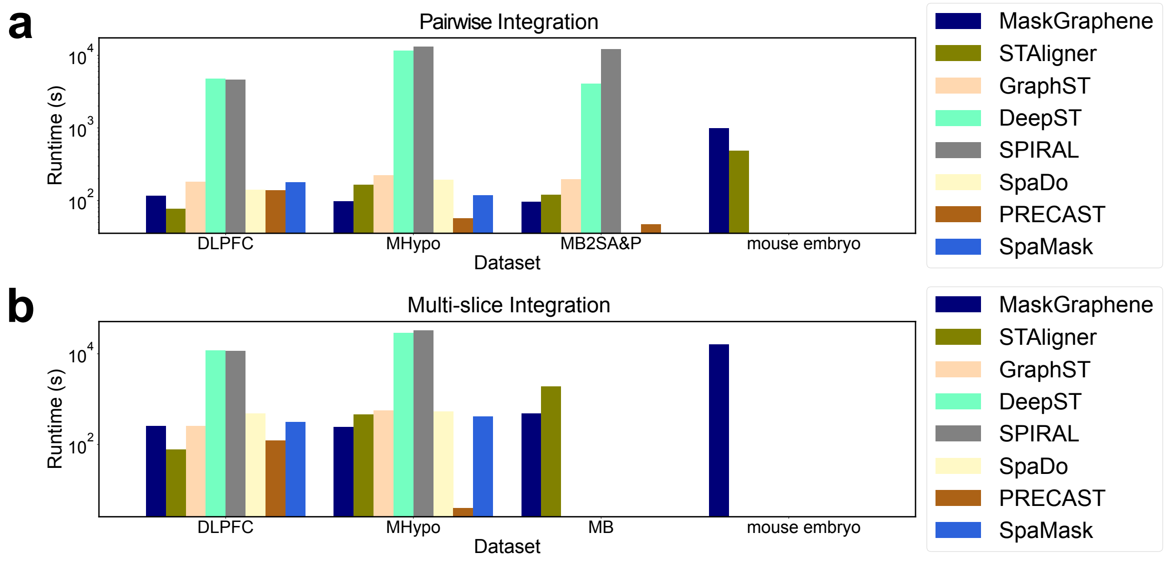

Fig. S32: **Runtime comparison of integration methods across datasets. (a)** Pairwise integration runtime (seconds, log scale) for the DLPFC, MHypo, MB2SA&P, and mouse embryo datasets. **(b)** Multi-slice integration runtime for the DLPFC, MHypo, MB, and mouse embryo (500k spots) datasets. Bar plots compare MaskGraphene, STAligner, GraphST, DeepST, SPIRAL, SpaDo, PRECAST, and SpaMask, with lower runtime indicating greater computational efficiency.

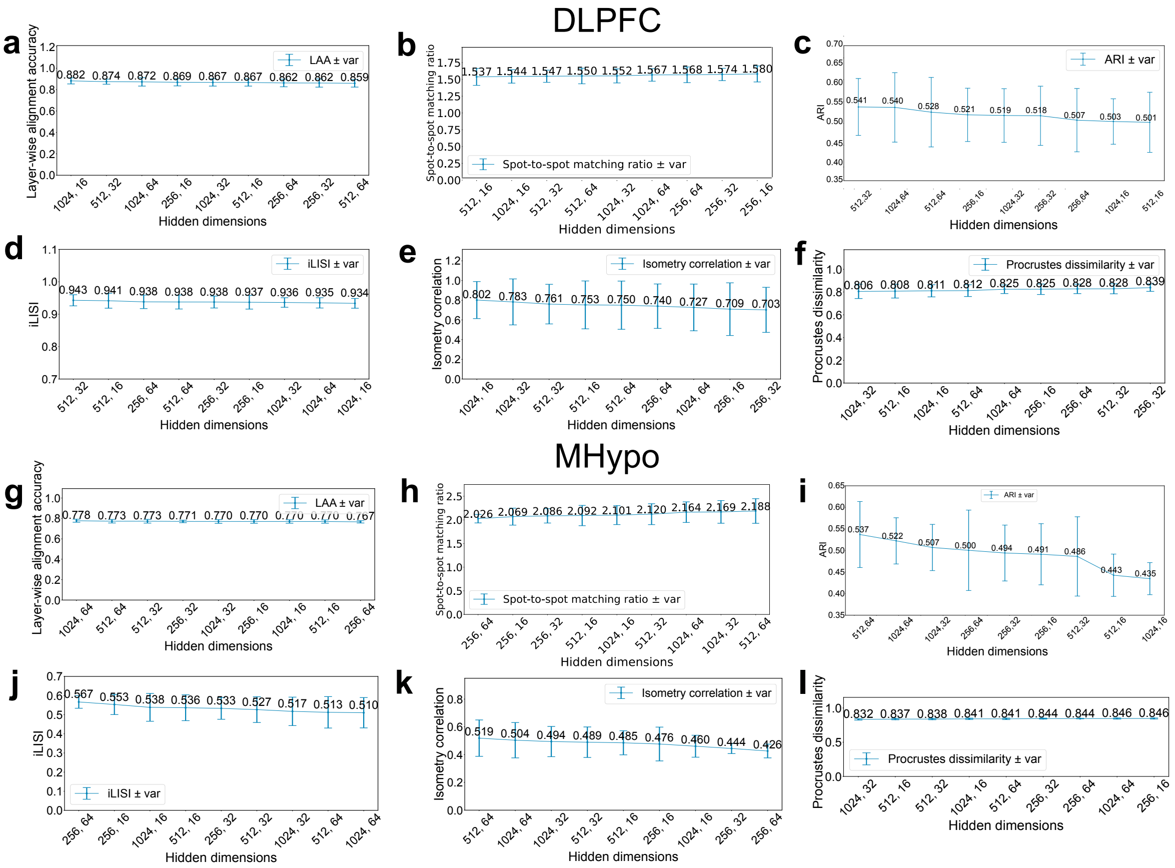

Fig. S33: **Grid search analysis of graph model layer configurations in MaskGraphene on the DLPFC and MHypo datasets.** **(a-f)** Metrics for the DLPFC dataset: **(a)** Layer-wise alignment accuracy, **(b)** Spot-to-spot matching ratio, **(c)** Adjusted Rand Index (ARI), **(d)** inverse local inverse Simpson's index (iLISI), **(e)** Isometry correlation, and **(f)** Procrustes dissimilarity. Hidden dimensions for each layer configuration are shown along the x-axis. Points indicate mean performance over replicates, and error bars represent variance. **(g-l)** Same metrics as above, reported for the MHypo dataset.

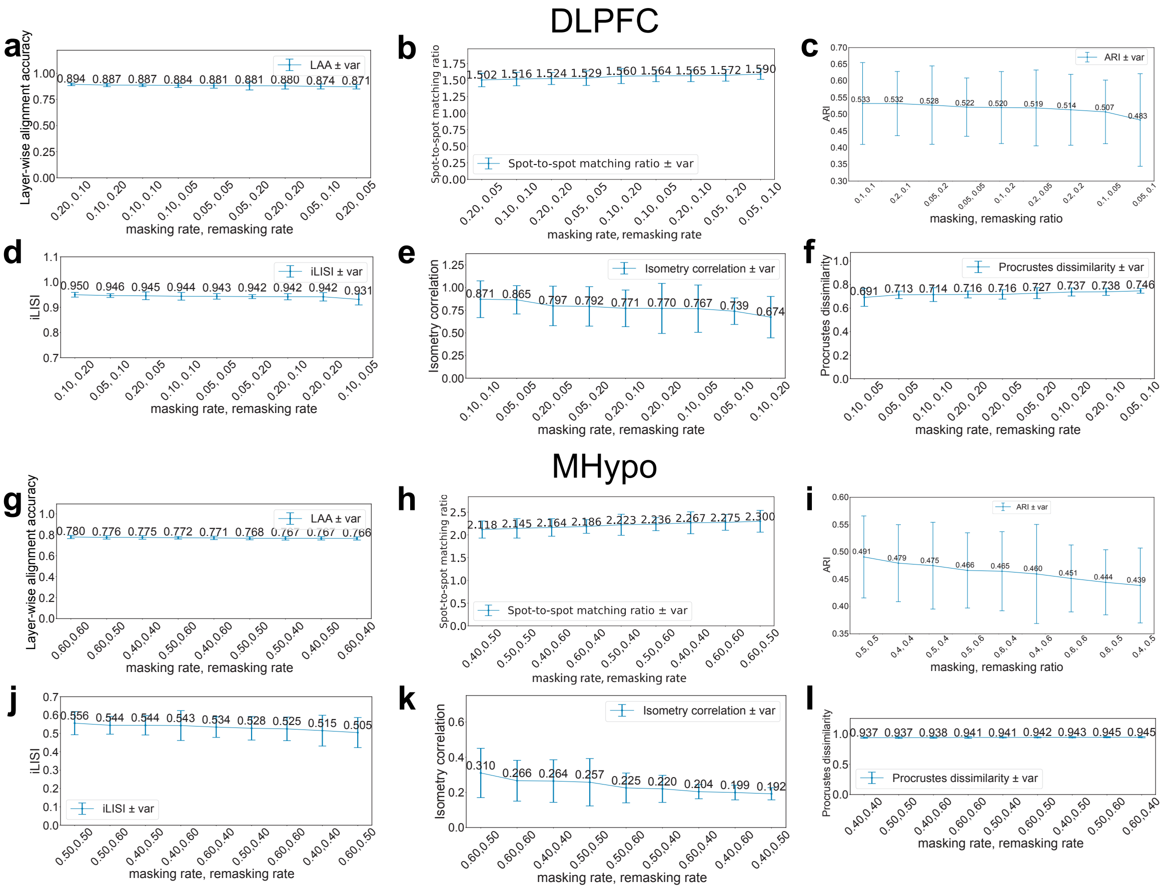

Fig. S34: **Grid search analysis of masking and ra-masking rates in MaskGraphene on the DLPFC and MHypo datasets.** **(a-f)** Metrics for the DLPFC dataset: **(a)** Layer-wise alignment accuracy, **(b)** Spot-to-spot matching ratio, **(c)** Adjusted Rand Index (ARI), **(d)** inverse local inverse Simpson's index (iLISI), **(e)** Isometry correlation, and **(f)** Procrustes dissimilarity. Masking and re-masking rates are shown along the x-axis. Points indicate mean performance over replicates, and error bars represent variance. **(g-l)** Same metrics as above, reported for the MHypo dataset.

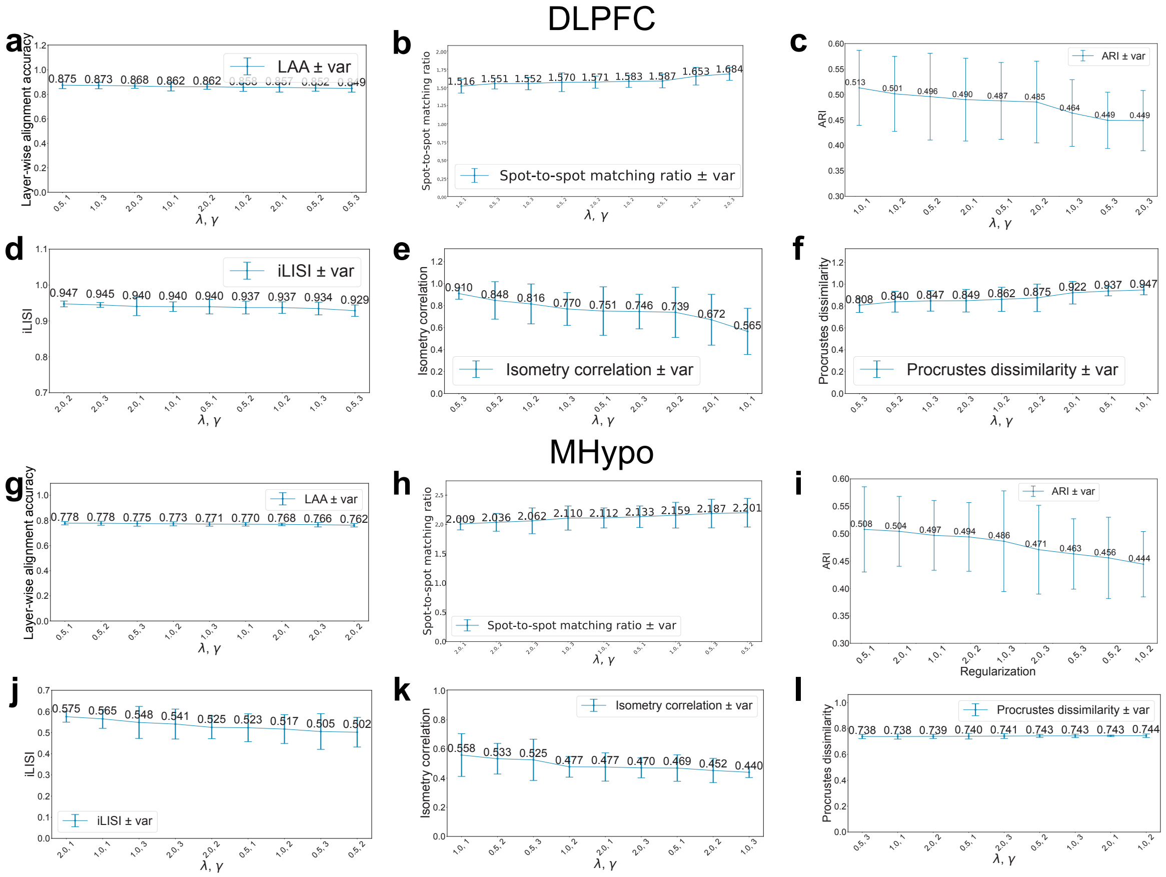

Fig. S35: **Grid search analysis of hyperparameters (λ, γ) in MaskGraphene on the DLPFC and MHypo datasets. (a-f)** Metrics for the DLPFC dataset: **(a)** Layer-wise alignment accuracy, **(b)** Spot-to-spot matching ratio, **(c)** Adjusted Rand Index (ARI), **(d)** inverse local inverse Simpson's index (iLISI), **(e)** Isometry correlation, and **(f)** Procrustes dissimilarity. Hyperparameters (λ, γ) are shown along the x-axis. Points indicate mean performance over replicates, and error bars represent variance. **(g-l)** Same metrics as above, reported for the MHypo dataset.

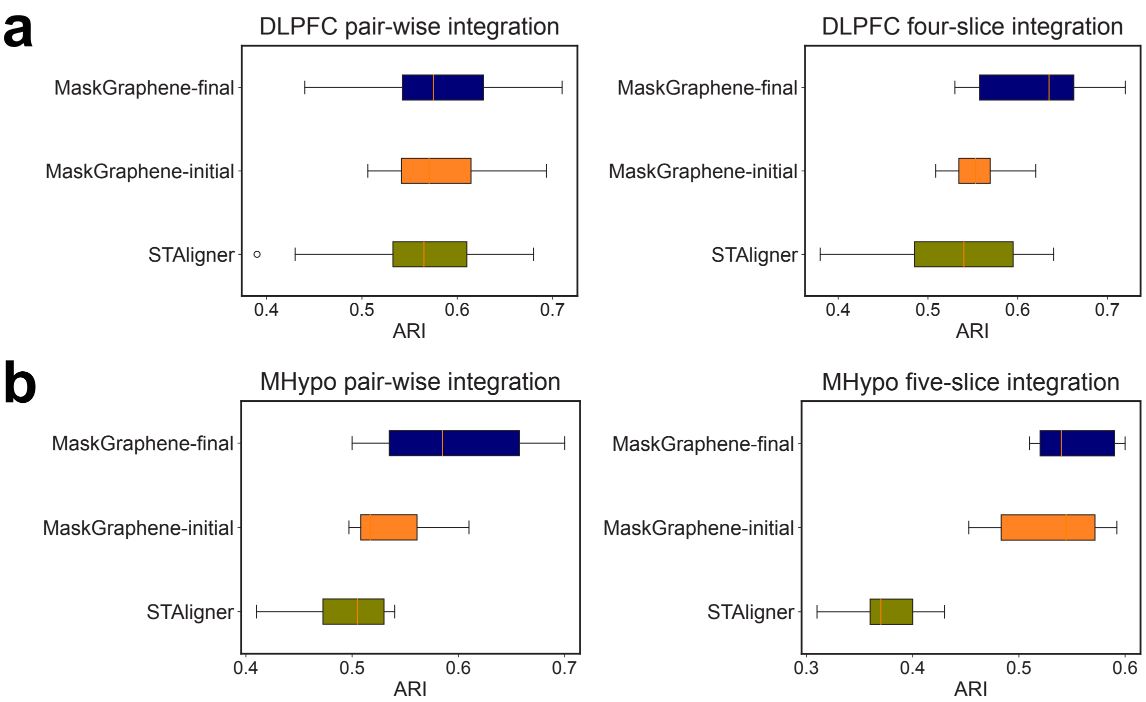

Fig. S36: **Boxplots of Adjusted Rand Index (ARI) for pairwise and multi-slice integration using MaskGraphene-initial, MaskGraphene-final, and STAligner on the DLPFC and MHypo datasets.** **(a)** ARI distributions for pairwise and four-slice integration on the DLPFC dataset. **(b)** ARI distributions for pairwise and five-slice integration on the MHypo dataset.

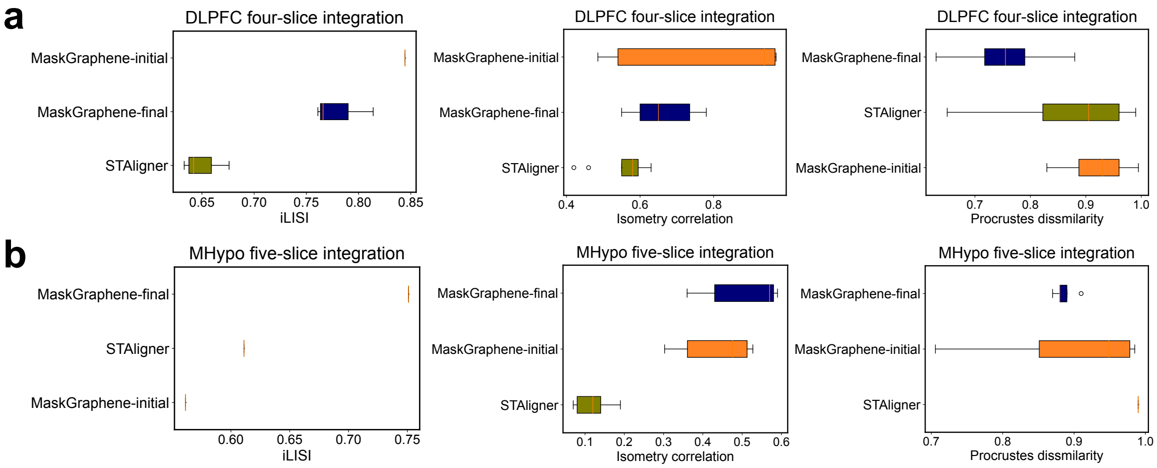

Fig. S37: **Boxplots of iLISI, Isometry correlation, and Procrustes dissimilarity for multi-slice integration on the DLPFC and MHypo datasets.** **(a)** DLPFC four-slice integration results for MaskGraphene (initial and final embeddings) and STAligner, evaluated using iLISI, Isometry correlation, and Procrustes dissimilarity. **(b)** MHypo five-slice integration results for the same methods and metrics. Higher iLISI and Isometry correlation, and lower Procrustes dissimilarity, indicate better integration quality.

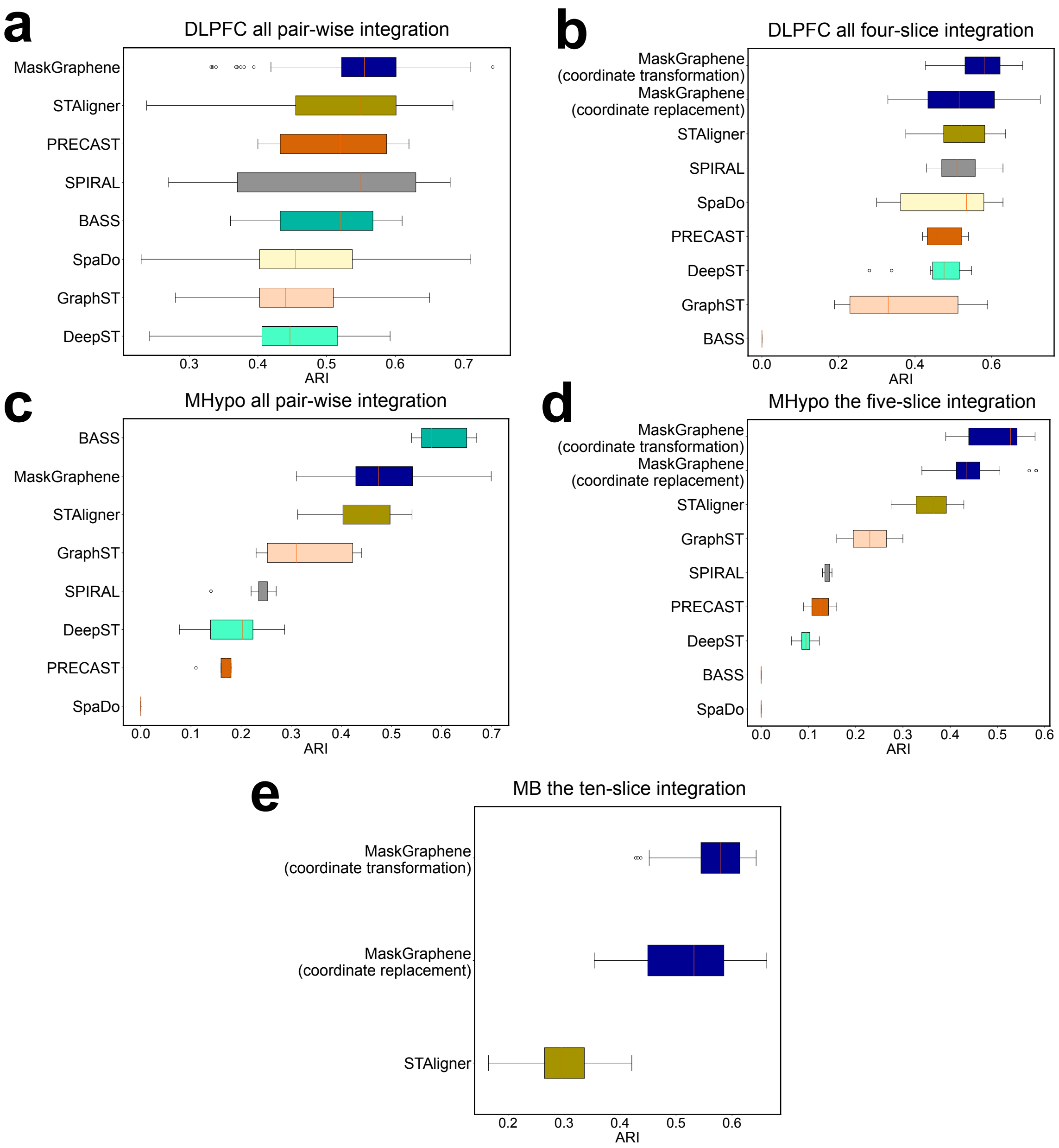

Fig. S38: **ARI box plots of clustering results after integration with varying random seeds across different datasets and methods.** **(a)** ARI box plots of clustering results after all DLPFC pairwise integration with varying random seeds. **(b)** ARI box plots of clustering results after all DLPFC four-slice integration with varying random seeds. **(c)** ARI box plots of clustering results after all MHypo pairwise integration with varying random seeds. **(d)** ARI box plots of clustering results after the MHypo five-slice integration with varying random seeds. **(e)** ARI box plots of clustering results after the MB ten-slice integration with varying random seeds.
